# Dyedauxiliary Group Strategy for the α-Functionalization of Ketones and Esters

**DOI:** 10.1021/acsorginorgau.1c00020

**Published:** 2021-08-26

**Authors:** Lorenzo Di Terlizzi, Ivana Cola, Carlotta Raviola, Maurizio Fagnoni, Stefano Protti

**Affiliations:** PhotoGreen Lab, Department of Chemistry, University of Pavia, Viale Taramelli 12, Pavia 27100, Italy

**Keywords:** visible light, arylazo sulfones, dyedauxiliary group, α-aryl ketones, metal-free arylation

## Abstract

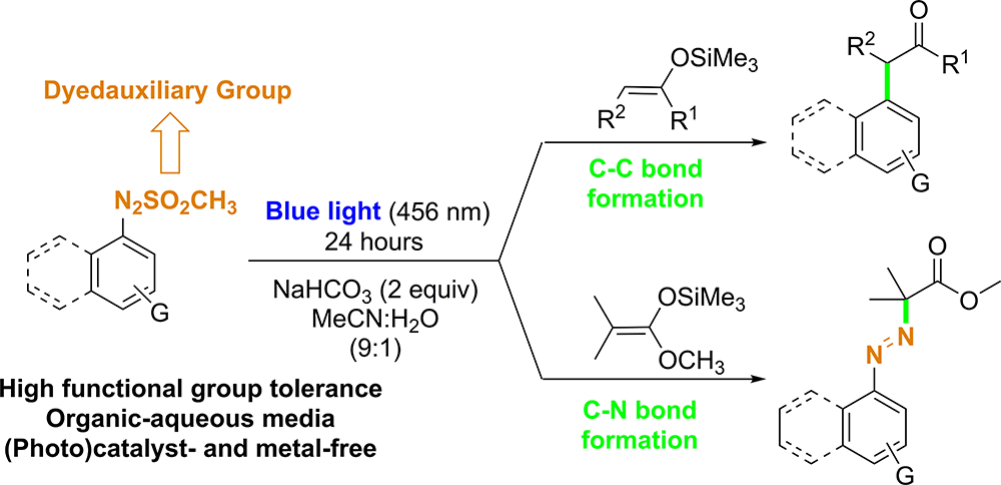

The synthesis of α-arylketones and α-arylazo esters has been achieved in mixed organic–aqueous media under photocatalyst- and metal-free conditions via visible light activation of arylazo sulfones in the presence of enol silyl ethers and ketene silyl acetals, respectively. The process took place efficiently and exhibits an excellent tolerance for a broad variety of functional groups.

## Introduction

**Scheme 1:**
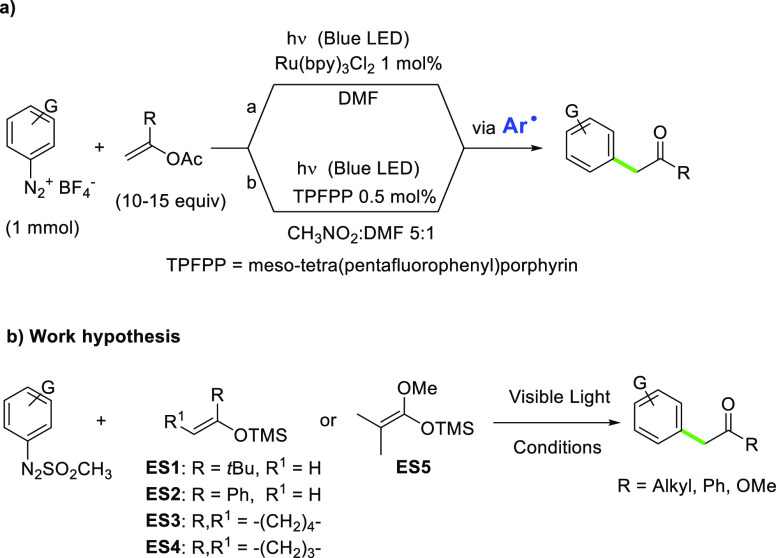
(a) Photoredox-Catalyzed Synthesis of α-Arylketones via Arenediazonium Salts and (b) Proposed Arylation of Enol Silyl Ethers and Ketene Silyl Acetals Starting from Arylazo Sulfones

α-Aryl carbonyls are structural motifs present in both natural and synthetic bioactive products (e.g., the antiplatelet agent Prasugrel),^1−3^ whose synthesis has been, in the 20th century, an exclusive domain of transition-metal-catalyzed processes,^4−8^ with the only exception represented by the (thermal- or photoinitiated) S_RN_1 coupling of aryl halides with aggressive enolate anions^9,10^ and the arylation of enol silyl ethers via photogenerated triplet aryl cations (in turn obtained only from 4-chloro and 4-fluoroanilines).^11,12^ However, the recent bursting of visible light photoredox-catalyzed processes has changed dramatically the way-of-thinking of organic chemistry practitioners.^13,14^ In such an approach, a thermally stable substrate is activated under mild conditions, by a photoexcited catalyst via a monoelectronic oxidation/reduction step, to generate a high-energy intermediate (e.g., a radical or a radical ion) able to react with a coupling partner.^13−17^ In this regard, the trapping of a photogenerated aryl radical by an enolate derivative (mainly enol acetates) has emerged as one of the most proposed strategies to achieve α-arylated carbonyls.^18−20^ Among the suitable precursors of aryl radicals made active under photoredox catalysis conditions, the class of aryl diazonium salts is the most preferred, and the reaction was carried out in the presence of Ru(II)-based complexes (Scheme 1, path a),^21^ porphyrins (path b),^22^ and organic dyes^23^ in the role of photocatalyst. It should be noticed, however, that the same reaction was found to occur in discrete to good yields also under thermal conditions, by using either salicylic acid^24^ or a UiO-66 metal organic framework^25^ as the promoters.

More recently, the use of dyedauxiliary groups, moieties that are able to impart both color and photoreactivity to a substrate, emerged as a promising and sustainable strategy to have access to reactive intermediates^26,27^ upon simple exposition of the starting materials to visible (solar) light, without the need of (photo)catalysts and/or aggressive reactants. In particular, our group focused on bench-stable, yellow-to-orange arylazo sulfones (Ar–N_2_SO_2_R), which are in turn smoothly prepared from the corresponding anilines. Such derivatives exhibited a wavelength-dependent photoreactivity.^28^ Thus, different intermediates (aryl diazenyl, aryl radicals, sulfonyl radicals, and aryl cations) can be generated selectively, by tuning the reaction conditions (light sources, reaction media, coupling partner). Such behavior was exploited in the optimization of synthetic protocols for aryl-carbon^29−31^ and aryl-heteroatom bond formation^32−35^ as well as aryldiazenylation^36^ and sulfonylation of alkenes.^37,38^

## Results and Discussion

Fascinated by the versatile applications of arylazo sulfones in organic synthesis, we now focused on the opportunity to arylate electron-rich silylated alkenes (Scheme 1b). Preliminary experiments allowed an investigation of the feasibility of the proposal (see Table S1 in the Supporting Information). Enol silyl ethers are the preferred coupling partners, and the optimized conditions for the synthesis of arylated products foresee the irradiation of a 0.05 M solution of arylazo sulfones **1a**–**1r** in a MeCN:H_2_O 9:1 mixture, in the presence of **ES1**–**ES4** (0.5 M, 10 equiv) and NaHCO_3_ (1 equiv) as the buffering agent.

**Scheme 2:**
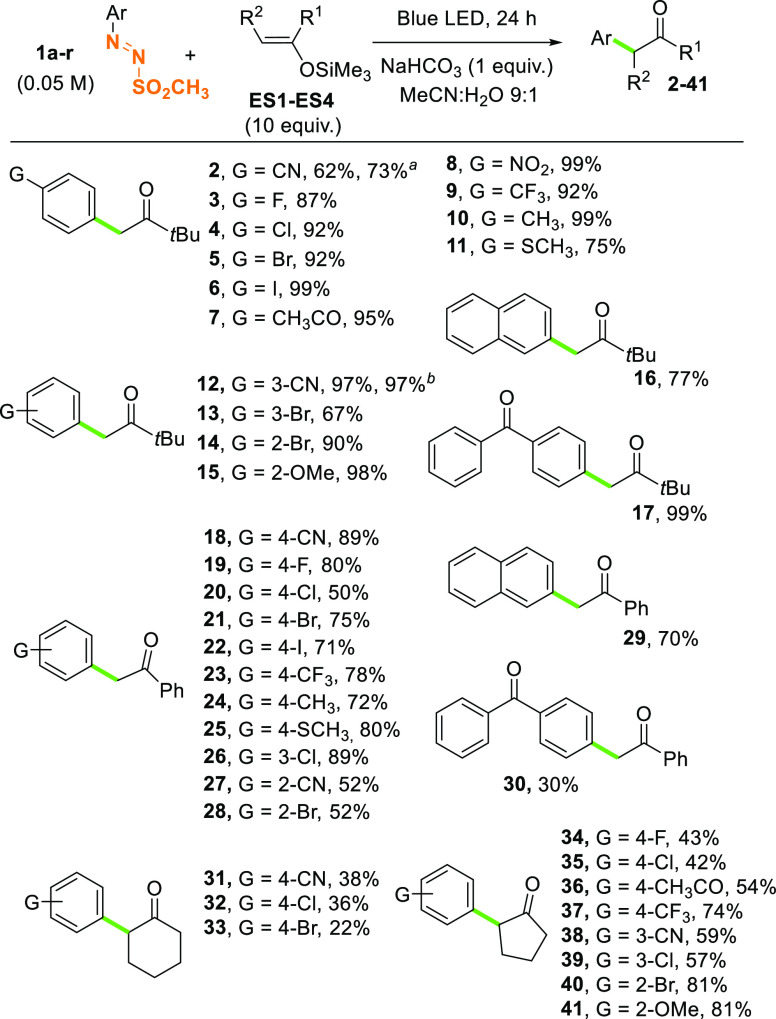
Synthesis of α-Arylketones **2**–**41** via Arylazo Sulfones Reaction carried out on a 0.1 M solution of **1a** in the presence of 5 equiv of **ES1**. Reaction carried out upon natural sunlight exposition (3 days, 9 h exposition/day).

As depicted in Scheme 2, *t*-butyl aryl ketones **2**–**17** (some of them important building blocks in the preparation of bioactive molecules, including TRPV1 antagonists)^39^ have been isolated in satisfactory to quantitative (see compounds **6**, **10**, and **17**) yields. The presence of either electron donating or electron withdrawing substituents on the aromatic ring does not affect the efficiency of the process (compare the yields observed for 4-acetyl and 4-thiomethoxy-derivatives **7** and **11**, respectively). α-(4-Cyanophenyl)-ketone **2** was isolated in 73% yield also when doubling the concentration of the starting arylazo sulfone **1a**, whereas the arylation resulted as satisfactory under both artificial visible light and natural sunlight (see the results obtained for compound **12**).

This visible light driven protocol was thus extended with success to 1-phenyl-1-trimethylsilyloxyethylene (**ES2**, in turn obtained from acetophenone). In this case, derivatives **18**–**30** were all isolated in a high amount, with the only exception of benzophenone **30**, which was obtained in a low yield (30%). When using cyclic enol silyl ethers **ES3** and **ES4**, the reaction generally had a lower performance, the best result obtained for 2-arylcyclopentanones **40** and **41** (81% yield).

**Scheme 3:**
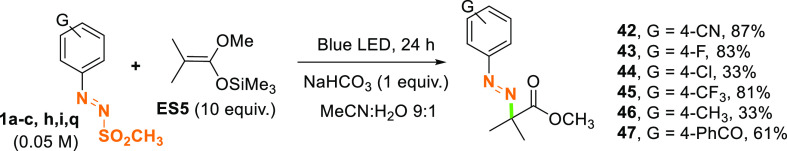
Irradiation of Arylazo Sulfones **1** in the Presence of Ketene Silyl Acetal **ES5**

With these results in hand, we focused on the use of ketene silyl acetals, with the aim of further exploring the versatility of the arylation protocol. However, with **ES5**, α-arylazo derivatives **42**–**47** were isolated in discrete (see compounds **44** and **46** in Scheme 3) to satisfactory (for **42** and **43**) yields instead of the expected α-aryl-esters.

**Scheme 4:**
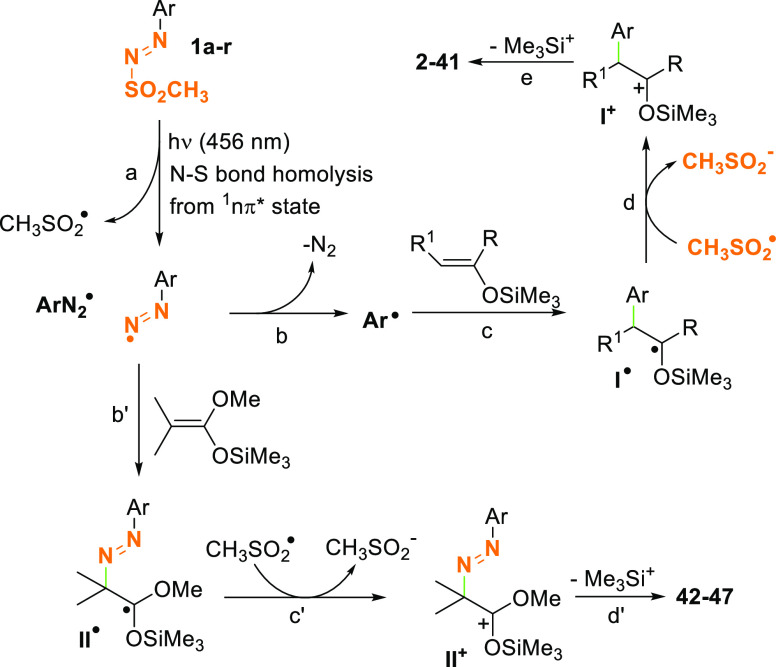
Suggested Mechanism for the Formation of Compounds **2**–**47**

The behavior of arylazo sulfones **1** can be explained both on the available literature and on the nature of the products isolated. Indeed, visible light irradiation of sulfones **1a**–**1s** causes the homolytic cleavage of the N–S bond from the ^1^(nπ*) excited state (Scheme 4, path a).^26^ Nitrogen loss from the thusly generated aryldiazenyl radical (Ar–N_2_^•^, path b) and efficient trapping of the resulting aryl radical (Ar^•^) by enol silyl ethers **ES1**–**ES4** (path c) afforded α-oxyradical **I**^**•**^. Oxidation of **I**^**•**^ (path d) and loss of the electrofugal Me_3_Si^+^ group (path e, that presumably undergoes hydrolysis to Me_3_SiOH) resulted in the formation of α-aryl ketones **2**–**41**.

The fate of the reaction follows the different nucleophilicity existing between silyl ethers **ES1**–**ES4** and ketene silyl acetal **ES5**. The reactivity of enol ether derivatives toward electrophilic radicals was sparsely explored in the past.^40−43^ Despite a comparable nucleophilicity,^44^ the ethers derived from cycloalkanones gave consistently worst results toward aryl^21^ and trifluoromethyl^42^ radical addition compared to those derived from acetophenones. The performance became similar only in the reactions with aggressive methoxycarbonyldifluoromethyl radicals.^43^ In the present work, however, arylation likewise took place efficiently even with the cyclopentanone derived **ES4**. On the other hand, in the presence of highly nucleophilic ketene silyl acetal **ES5**,^45,46^ trapping of Ar–N_2_^•^ takes place before dediazoniation (path b′), and derivatives **42**–**47** were obtained via consecutive oxidation and Me_3_Si^+^ elimination of the α-oxy radical intermediate **II**^**•**^ (paths c′ and d′). The methanesulfonyl radical (CH_3_SO_2_^•^) arising from the N–S homolytic cleavage presumably acted as an electron acceptor in both oxidation paths d and c′.^26,27^ As already stated in the literature, water plays a key role in the stabilization of the cationic intermediates **I**^**+**^ and **II**^**+**^ and in favoring the desilylation step.^47^ Finally, the presence of NaHCO_3_ prevents any acid-catalyzed decomposition of the silyl derivatives employed.

## Conclusion

The developed strategy thus further evidences the versatility of arylazo sulfones as (visible light) precursors of reactive intermediates, whose reactivity can be tuned by the employed reaction partners. Indeed, irradiation of **1** in the presence of enol silyl ethers results in the formation or α-aryl ketones, under metal- and photocatalyst-free conditions. The reaction occurs in satisfactory yields and high functional group tolerance. On the other hand, as already observed in the past with captodative olefins,^36^ the photochemical activation of arylazo sulfones may lead to nitrogen incorporated derivatives preventing any nitrogen loss. Thus, the reaction with ketene silyl acetals gives access (in good yield) to α-arylazo esters, important building blocks in the preparation of azo prodrugs (e.g., for the colonic delivery of agents for the treatment of *Clostridium difficile* infection)^48^ and in the synthesis of valuable compounds including, among the others, pyrazolidinones,^49^ β-amino alcohols, and α-amino acids.^50^

## Supporting Information Available

Detailed procedures and characterization of all products; and copies of ^1^H and ^13^C NMR (PDF)
