# Supplementary material for: Dyedauxiliary Group Strategy for the α-Functionalization of Ketones and Esters
Source: ACS Org Inorg Au. 2021 Aug 26;1(2):68–71. doi: 10.1021/acsorginorgau.1c00020 (PMC9954345; doi:10.1021/acsorginorgau.1c00020)
Supplement: Supplementary file 1 — gg1c00020_si_001.pdf [file gg1c00020_si_001.pdf]

Supporting Information

A Dyedauxiliary Group Strategy for the α-Functionalization of Ketones and Esters.

*Lorenzo Di Terlizzi, Ivana Cola, Carlotta Raviola, Maurizio Fagnoni and Stefano Protti**

PhotoGreen Lab, Department of Chemistry, University of Pavia, Viale Taramelli 12, Pavia 27100.

Email: stefano.protti@unipv.it

1. **Experimental details** **S2**
2. **Copy of the ^{1}H and ^{13}C NMR of compounds 2-47** **S15**
3. **References** **S107**

1. Experimental section

General: ^{1}H and ^{13}C NMR spectra were recorded on a 300 e 75 MHz spectrometer, respectively. The attributions were made on the basis of ^{1}H and ^{13}C NMR experiments; chemical shifts are reported in ppm downfield from TMS. GC analysis were performed using a HP SERIES 5890 II equipped with a fire ion detector (FID, temperature 350 °C). Analytes were separated using a Restek Rtx-5MS (30 m×0.25 mm×0.25 μm) capillary column with nitrogen as a carrier gas at 1 ml min^{-1}. The injector temperature was 250 °C. The GC oven temperature was held at 80 °C for 2 min, increased to 250 °C by a temperature ramp of 10 °C min^{-1}, and held for 10 min. Silyl ethers **ES1-ES5** are commercially available and used as received.

General Procedure for the Synthesis of Arylazo Sulfones. Arylazo sulfones **1a-r**, were previously synthesized[S1] and fully characterized[S1,S2] by our research group by the following procedure. Photophysical data (UV-Vis absorption spectra, λ_{max}, ε) for selected arylazo sulfones are available in the previous works of our research groups.[S1, S2a]

Diazonium salts were freshly prepared prior to use from the corresponding anilines and purified by dissolving in acetonitrile and precipitation by adding cold diethyl ether. To a cooled (0 °C) suspension of the chosen diazonium salt (1 equiv., 0.3 M) in CH_2Cl_2 was added sodium methanesulfinate (1.2 equiv.) in one portion. The temperature was allowed to rise to room temperature, and the solution stirred overnight. The resulting mixture was then filtered, and the obtained solution was evaporated affording the desired arylazo sulfone. The crude product was finally dissolved in CH_2Cl_2 and precipitated by adding cold *n*-hexane.

Table S1. Optimization of the photochemical procedure.

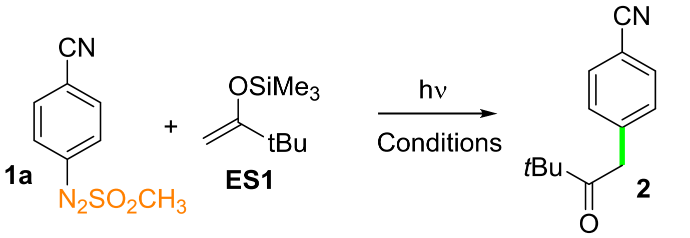

Entry	Conditions	2 (% yield)
1	**1a** (0.05 M), **ES1** (0.5 M), hν (456 nm), MeCN	0%
2	**1a** (0.05 M), **ES1** (0.5 M), hν (456 nm), $NaHCO_3$ (0.05 M) MeCN	8%
3	**1a** (0.05 M), **ES1** (0.5 M), hν (456 nm), $NaHCO_3$ (0.05 M) MeOH	8%
4	**1a** (0.05 M), **ES1** (0.5 M), hν (456 nm), $NaHCO_3$ (0.05 M), MeCN-H_2O 9:1	63%
5	**1a** (0.05 M), **ES1** (0.5 M), hν (390 nm), $NaHCO_3$ (0.05 M), MeCN-H_2O 9:1	60%
6	**1a** (0.1 M), **ES1** (0.5 M), hν (456 nm), $NaHCO_3$ (0.05 M), MeCN-H_2O 9:1	39%
7	**1a** (0.05 M), **ES1** (0.5 M), $NaHCO_3$ (0.05 M), MeCN-H_2O 9:1	0%
8	**1a** (0.05 M), **ES1** (0.5 M), T = 60°C $NaHCO_3$ (0.05 M), MeCN-H_2O 9:1	0%

Reaction optimization was carried out to find the best reaction conditions. Performing the reaction in MeCN without $NaHCO_3$ gave no product (entry **1**). Shifting to acetonitrile and methanol and adding the base did not improve significantly the yield (ca. 8%, entries **2** and **3**). However, switching from pure acetonitrile to a mixture of acetonitrile and water (9:1) increased the arylation yield to 63% (entry **4**). Moving the irradiation wavelength from 456 nm to 390 nm results in a lowering of the reaction efficiency (60% yield, entry **5**). When doubling the concentration of the arylazo sulfone **1a** (from 0.05 M to 0.1 M) the reaction yield decreased to 39% (entry **6**). Finally, in the absence light both by covering the vessel with an aluminum foil at room temperature (entry 7) and by heating the vessel at 60°C for 24 h resulted in no **2** formation (entries **7** and **8**).

General Procedure for the photochemical arylation of enol silyl ethers. A pyrex glass vessel was charged with the chosen arylazo sulfone (**1a-r**, 0.4 mmol, 1.0 equiv., 0.05 M) and 64 mg of $NaHCO_3$ (0.1 mmol, 0.1 M) was flushed with argon and the solid was dissolved in degassed acetonitrile:water 9:1 mixture (8.0 mL). Then, the desired enol silyl ether was added (0.5 mmol, 10 equiv., 0.5 M). The reaction was irradiated for 24 h using an EvoluChem apparatus equipped with a 40 W Kessil lamp (λ_{em}= 456 nm, see Figure S1; the lamp emission spectrum is available at the link https://www.kessil.com/science/PR160L.php). The photolyzed solution was concentrated under reduced pressure and purified by silica gel column chromatography (cyclohexane-ethyl acetate mixture as eluant).

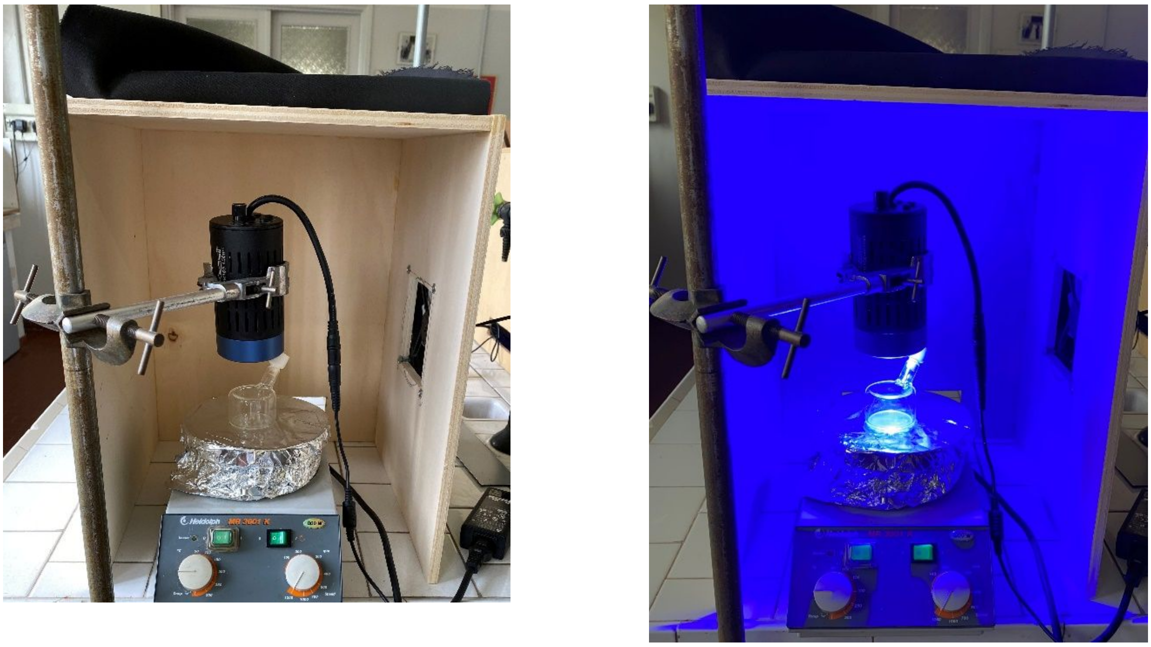

Figure S1: Irradiation system used to perform the reactions on this work: A 40 W Kessil lamp (with emission centered at 456 nm) is held three centimetres above the reaction vessel which was stirred gently for 24 h. A fan is placed on the right of the reaction vessel to avoid any heating of the solution.

4-(3,3-Dimethyl-2-oxobutyl)benzonitrile (2): From 80.1 mg (0.383 mmol) of **1a**, 880 µL (10 equiv.) of **ES1,** 64 mg of $NaHCO_3$ (0.1 mmol, 0.1 M) in 8 mL of degassed acetonitrile:water (9:1). Purification was carried out by silica gel chromatographic column (eluant: 95:5 Cyclohexane/ethyl acetate) to afford 51.0 mg of **2** (62% yield, white solid, mp 45-46°C). Compound **2** was likewise isolated in 73% yield when irradiating a 0.05 M solution of **1a** in the presence of 5 equiv. of **ES1**. ^{1}H NMR (300 MHz, $CDCl_3$) δ 7.60 (d, *J* = 8.3 Hz, 2H), 7.28 (d, *J* = 8.5 Hz, 2H), 3.86 (s, 2H), 1.22 (s, 9H). ^{13}C NMR (75 MHz, $CDCl_3$) δ 211.6, 140.6, 132.2, 130.6, 119.0, 110.8, 44.9, 43.3, 26.4. HRMS (EI) m/z: $[M]^+$ calcd for $C_{13}H_{15}NO$ 202.1226, found 202.1222.

1-(4-Fluorophenyl)-3,3-dimethylbutan-2-one (3): From 79.0 mg (0.391 mmol) of **1b,** 880 µL (10 equiv.) of **ES1**, 64 mg of $NaHCO_3$ (0.1 mmol, 0.1 M) in 8 mL of degassed acetonitrile:water (9:1). Purification was carried out by silica gel chromatographic column (eluant: 95:5 Cyclohexane/ethyl acetate) to afford 66.0 mg of **3** (87 % yield, slightly yellow oil). Spectroscopic data were in accordance with literature data.[S3]

1-(4-Chlorophenyl)-3,3-dimethylbutan-2-one (4): From 88.0 mg (0.402 mmol) of **1c**, 880 µL (10 equiv.) of **ES1**, 64 mg of $NaHCO_3$ (0.1 mmol, 0.1 M) in 8 mL of degassed acetonitrile:water (9:1). Purification was carried out by silica gel chromatographic column (eluant: 95:5 Cyclohexane/ethyl acetate) to afford 77.9 mg of **4** (92% yield, slightly yellow solid, mp = 47-49 °C). Spectroscopic data were in accordance with literature data.[S4]

1-(4-Bromophenyl)-3,3-dimethylbutan-2-one (5): From 98.5 mg (0.376 mmol) **1d**, 880 µL (10 equiv.) of **ES1**, 64 mg of $NaHCO_3$ (0.1 mmol, 0.1 M) in 8 mL of degassed acetonitrile:water (9:1). Purification was carried out by silica gel chromatographic column (eluant: 95:5 Cyclohexane/ethyl acetate) to afford 87.9 mg of **5** (92% yield, slightly yellow solid, mp = 54-56 °C). ^{1}H NMR (300 MHz, $CDCl_3$) δ 7.13 (dd, *J* = 8.7, 5.4 Hz, 2H), 6.99 (t, *J* = 8.7 Hz, 2H), 3.77 (s, 2H), 1.20 (s, 9H). ^{13}C NMR (75 MHz, $CDCl_3$) δ 212.8, 163.54 160.3, 131.2, 131.1, 130.7, 115.5, 115.2, 44.7, 42.4, 26.5. HRMS (EI) m/z: $[M]^+$ calcd for $C_{12}H_{15}OBr$ 255.0379, found 255.0374.

1-(4-Iodophenyl)-3,3-dimethylbutan-2-one (6): From 122.1 mg (0.395 mmol) of **1e,** 880 µL (10 equiv.) of **ES1**, and 64 mg of $NaHCO_3$ (0.1 mmol, 0.1 M) in 8 mL of degassed acetonitrile:water (9:1). Purification was carried out by silica gel chromatographic column (eluant: 95:5 Cyclohexane/ethyl acetate) to afford 119.3 mg of **6** (99%, slightly yellow solid, mp = 58-60 °C). ^{1}H NMR (300 MHz, $CDCl_3$) δ 7.64 (d, J = 8.3 Hz, 2H), 6.94 (d, J = 8.3 Hz, 2H), 3.76 (s, 2H), 1.22 (s,

9H). ^{13}C NMR (75 MHz, $CDCl_3$) δ 212.10, 137.32, 134.46, 131.51, 44.55, 42.57, 26.25. HRMS (EI) m/z: $[M]^+$ calcd for $C_{12}H_{15}OI$ 303.0240, found 303.0229.

1-(4-Acetylphenyl)-3,3-dimethylbutan-2-one (7): from 89.0 mg (0.394 mmol) of **1f**, 880 μL (10 equiv.) of **ES1** and 64 mg of $NaHCO_3$ (0.1 mmol, 0.1 M) in 8 mL of degassed acetonitruile:water (9:1). Purification was carried out by silica gel chromatographic column (eluant: 95:5 Cyclohexane/ethyl acetate) to afford 81.6 mg of **7** (95% yield, yellow oil). ^1H NMR (300 MHz, $CDCl_3$) δ 7.88 (d, J = 8.3 Hz, 2H), 7.25 (d, J = 8.2 Hz, 2H), 3.85 (s, 2H), 2.56 (s, 3H), 1.19 (s, 9H). ^{13}C NMR (75 MHz, $CDCl_3$) δ 212.1, 197.9, 140.6, 129.9, 128.5, 44.8, 43.2, 26.7, 26.4. MS (m/z): 218 (5), 133 (60), 118 (10), 104 (16), 85 (30), 57 (100), 44 (52). HRMS (EI) m/z: $[M]^+$ calcd for $C_{14}H_{18}O_2$ 219.1380, found 219.1371.

3,3-Dimethyl-1-(4-nitrophenyl)butan-2-one (8): From 77.8 mg (0.339 mmol) of **1g**, 880 μL (10 equiv.) of **ES1** and 64 mg of $NaHCO_3$ (0.1 mmol, 0.1 M) in 8 mL of degassed acetonitrile:water (9:1). Purification was carried out by silica gel chromatographic column (eluant: 95:5 Cyclohexane/ethyl acetate) to afford 87.3 mg of **8** (99 % yield, slightly yellow solid, mp = 60-61°C). ^1H NMR (300 MHz, $CDCl_3$) δ 8.17 (d, J = 8.6 Hz, 2H), 7.34 (d, J = 8.5 Hz, 2H), 3.92 (s, 2H), 1.23 (s, 9H). ^{13}C NMR (75 MHz, $CDCl_3$) δ 211.2, 142.4, 130.4, 123.4, 42.8, 26.2. HRMS (EI) m/z: $[M]^+$ calcd for $C_{12}H_{15}NO_3$ 222.1125, found 222.1132.

3,3-Dimethyl-1-(4-(trifluoromethyl)phenyl)butan-2-one (9): From 105.1 mg (0.417 mmol) of **1h**, 880 μL (10 equiv.) of **ES1** and 64 mg of $NaHCO_3$ (0.1 mmol, 0.1 M) in 8 mL of degassed acetonitrile:water (9:1). Purification was carried out by silica gel chromatographic column (eluant: 95:5 Cyclohexane/ethyl acetate) to afford 93.8 mg of **9** (92% yield, white solid, mp = 51-53 °C). Spectroscopic data were in accordance with literature data.[S5]

3,3-Dimethyl-1-(*p*-tolyl)butan-2-one (10): From 76.7 mg (0.387 mmol) of **1i**, 880 μL (10 equiv.) of **ES1** and 64 mg of $NaHCO_3$ (0.1 mmol, 0.1 M) in 8 mL of degassed acetonitrile:water (9:1). Purification was carried out by silica gel chromatographic column (eluant: 95:5 Cyclohexane/ethyl acetate) to afford 72.9 mg of **10** (99% yield, red oil). Spectroscopic data were in accordance with literature data.[S6]

3,3-Dimethyl-1-(4-(methylthio)phenyl)butan-2-one (11): From 88.1 mg (0.383 mmol) of **1j**, 880 μL (10 equiv.) of **ES1** and 64 mg of $NaHCO_3$ (0.1 mmol, 0.1 M) in 8 mL of degassed acetonitrile:water (9:1). Purification was carried out by silica gel chromatographic column (eluant: 95:5 Cyclohexane/ethyl acetate) to afford 63.8 mg of **11** (75% yield, red oil). ^1H NMR (300 MHz,

$CDCl_3$) δ 7.35 – 7.23 (m, 2H), 7.20 – 7.00 (m, 2H), 4.02 (s, 2H), 2.44 (s, 3H), 1.29 (s, 9H). ^{13}C NMR (75 MHz, $CDCl_3$) δ 212.5, 137.8, 134.4, 130.8, 127.7, 127.4, 125.5, 44.7, 41.9, 26.9, 16.8. HRMS (EI) m/z: $[M]^+$ calcd for $C_{13}H_{18}OS$ 223.1151, found 223.1142.

3-(3,3-Dimethyl-2-oxobutyl)benzonitrile (12): From 82.5 mg (0.394 mmol) of **1k**, 880 µL (10 equiv.) of **ES1** and 64 mg of $NaHCO_3$ (0.1 mmol, 0.1 M) in 8 mL of degassed acetonitrile:water (9:1). Purification was carried out by silica gel chromatographic column (eluant: 95:5 Cyclohexane/ethyl acetate) to afford 73.3 mg of **12** (97% yield, slightly yellow solid, mp = 38.5-39.7 °C). See further Scheme S1 below. Spectroscopic data were in accordance with literature data.[S7]

1-(3-Bromophenyl)-3,3-dimethylbutan-2-one (13): From 103.1 mg (0.394 mmol) of **1m** 880 µL (10 equiv.) of **ES1** and 64 mg of $NaHCO_3$ (0.1 mmol, 0.1 M) in 8 mL of degassed acetonitrile:water (9:1). Purification was carried out by silica gel chromatographic column (eluant: 95:5 Cyclohexane/ethyl acetate) to afford 67.2 mg of **13** (67% yield, yellow oil). Spectroscopic data were in accordance with literature data[S8]

1-(2-Bromophenyl)-3,3-dimethylbutan-2-one (14): From 104.2 mg (0.397 mmol) of **1o**, 880 µL (10 equiv.) of **ES1** and 64 mg of $NaHCO_3$ (0.1 mmol, 0.1 M) in 8 mL of degassed acetonitrile:water (9:1). Purification was carried out by silica gel chromatographic column (eluant: 95:5 Cyclohexane/ethyl acetate) to afford 91.0 mg of **14** (90% yield, orange oil). ^1H NMR (300 MHz, $CDCl_3$) δ 7.57 (dd, *J* = 7.9, 1.3 Hz, 1H), 7.29 (td, *J* = 7.4, 1.3 Hz, 1H), 7.15 (ddd, *J* = 14.8, 7.2, 1.8 Hz, 2H), 4.02 (s, 2H), 1.29 (s, 12H). ^{13}C NMR (75 MHz, $CDCl_3$) δ 211.4, 135.1, 132.6, 131.8, 128.4, 127.2, 124.9, 44.5, 44.0, 26.7. HRMS (EI) m/z: $[M]^+$ calcd for $C_{12}H_{15}OBr$ 255.0379, found 255.0382.

1-(2-Methoxyphenyl)-3,3-dimethylbutan-2-one (15): From 85.0 mg (0.397 mmol) of **1p**, 880 µL (10 equiv.) of **ES1** and 64 mg of $NaHCO_3$ (0.1 mmol, 0.1 M) in 8 mL of degassed acetonitrile:water (9:1). Purification was carried out by silica gel chromatographic column (eluant: 95:5 Cyclohexane/ethyl acetate) to afford 80.2 mg of **15** (98% yield, slightly yellow solid, mp = 34-35 °C[S9]). Spectroscopic data were in accordance with literature data.[S9]

1-(3-benzoylphenyl)-3,3-dimethylbutan-2-one (16): From 110.6 mg (0.384 mmol) of **1q** 880 µL (10 equiv.) of **ES1** and 64 mg of $NaHCO_3$ (0.1 mmol, 0.1 M) in 8 mL of degassed acetonitrile:water (9:1). Purification was carried out by silica gel chromatographic column (eluant: 95:5 Cyclohexane/ethyl acetate) to afford 66.8 mg of **16** (77%, white solid, mp = 78-79 °C). ^1H NMR (300 MHz, $CDCl_3$) δ 7.99–7.68 (m, 4H), 7.59 (t, *J* = 7.4 Hz, 1H), 7.48 (t, *J* = 7.4 Hz, 2H), 7.30 (d, *J* = 8.2 Hz, 2H), 3.90 (s, 2H), 1.24 (s, 9H). ^{13}C NMR (75 MHz, $CDCl_3$) δ 212.0, 139.8, 137.6, 135.8, 132.2,

130.1, 129.9, 129.5, 128.1, 44.7, 43.1, 26.2. MS (m/z): 208 (5), 196 (80), 167 (10), 118 (22),105 (26), 85 (25), 77(20), 57 (100). HRMS (EI) m/z: $[M]^+$ calcd for $C_{19}H_{20}O_2$ 281.1536, found 281.1524.

3,3-Dimethyl-1-(naphthalen-1-yl)butan-2-one (17): From 94.1 mg (0.402 mmol) of **1r**, 880 μL (10 equiv.) of **ES1** and 64 mg of $NaHCO_3$ (0.1 mmol, 0.1 M) in 8 mL of degassed acetonitrile:water (9:1). Purification was carried out by silica gel chromatographic column (eluant: 95:5 Cyclohexane/ethyl acetate) to afford 112.6 mg of **17** (99% yield, red solid, mp = 67-69 °C[S10a]). Spectroscopic data were in accordance with literature data.[S10]

4-(2-Oxo-2-phenylethyl)benzonitrile (18): From 85.3 mg (0.408 mmol) of **1a** 800 μL (10 equiv.) of **ES2** and 64 mg of $NaHCO_3$ (0.1 mmol, 0.1 M) in 8 mL of degassed acetonitrile:water (9:1). Purification was carried out by silica gel chromatographic column (eluant: 9:1 Cyclohexane/ethyl acetate) to afford 80.2 mg of **18** (89% yield, slightly yellow solid, mp = 113-114 °C[S11a]). Spectroscopic data were in accordance with literature data.[S11]

2-(4-Fluorophenyl)-1-phenylethan-1-one (19): From 83.8 mg (0.415 mmol) of **1b**, 800 μL (10 equiv.) of **ES2** and 64 mg of $NaHCO_3$ (0.1 mmol, 0.1 M) in 8 mL of degassed acetonitrile:water (9:1). Purification was carried out by silica gel chromatographic column (eluant: 9:1 Cyclohexane/ethyl acetate) to afford 71.1 mg of **19** (80% yield, yellow solid, mp = 109-110 °C[S12]). Spectroscopic data were in accordance with literature data.[S13]

2-(4-Chlorophenyl)-1-phenylethan-1-one (20): From 87.2 mg (0.400 mmol) of **1c**, 800 μL (10 equiv.) of **ES2** and 64 mg of $NaHCO_3$ (0.1 mmol, 0.1 M) in 8 mL of degassed acetonitrile:water (9:1). Purification was carried out by silica gel chromatographic column (eluant: 9:1 Cyclohexane/ethyl acetate) to afford 49.0 mg of **20** (50% yield, yellow solid, mp = 137-139 °C[S14]) Spectroscopic data were in accordance with literature data.[S13]

2-(4-Bromophenyl)-1-phenylethan-1-one (21): From 106.4 mg (0.406 mmol) of **1d**, 800 μL (10 equiv.) of **ES2** and 64 mg of $NaHCO_3$ (0.1 mmol, 0.1 M) in 8 mL of degassed acetonitrile:water (9:1). Purification was carried out by silica gel chromatographic column (eluant: 9:1 Cyclohexane/ethyl acetate) to afford 83.3 mg of **21** (75% yield, yellow solid, mp = 150-152 °C[S15]). Spectroscopic data were in accordance with literature data.[S13]

2-(4-Iodophenyl)-1-phenylethan-1-one (22): From 120.0 mg (0.387 mmol) of **1e**, 800 μL (10 equiv.) of **ES2** and 64 mg of $NaHCO_3$ (0.1 mmol, 0.1 M) in 8 mL of degassed acetonitrile:water (9:1). Purification step was carried out by silica gel chromatographic column (eluant: 9:1 Cyclohexane/ethyl

acetate) to afford 88.4 mg of **22** (71% yield, red solid, mp = 158-159 °C[S16]). Spectroscopic data were in accordance with literature data.[S17]

1-Phenyl-2-(4-(trifluoromethyl)phenyl)ethan-1-one (23): From 102.1 mg (0.405 mmol) of **1h**, 800 μL (10 equiv.) of **ES2** and 64 mg of $NaHCO_3$ (0.1 mmol, 0.1 M) in 8 mL of degassed acetonitrile:water (9:1). Purification was carried out by silica gel chromatographic column (eluant: 9:1 Cyclohexane/ethyl acetate) to afford 83.8 mg of **23** (78% yield, yellow solid, mp = 130-132 °C[S18]). Spectroscopic data were in accordance with literature data.[S13]

1-Phenyl-2-(*p*-tolyl)ethan-1-one (24): From 84.2 mg (0.425 mmol) of **1i**, 800 μL (10 equiv.) of **ES2** and 64 mg of $NaHCO_3$ (0.1 mmol, 0.1 M) in 8 mL of degassed acetonitrile:water (9:1). Purification was carried out by silica gel chromatographic column (eluant: 9:1 Cyclohexane/ethyl acetate) to afford 64.3 mg of **24** (72% yield, yellow solid, mp = 95-96 °C[S19]). Spectroscopic data were in accordance with literature data. [S13]

2-(4-(Methylthio)phenyl)-1-phenylethan-1-one (25): From 97.8 mg (0.425 mmol) of **1j**, 800 μL (10 equiv.) of **ES2** and 64 mg of $NaHCO_3$ (0.1 mmol, 0.1 M) in 8 mL of degassed acetonitrile:water (9:1). Purification was carried out by silica gel chromatographic column (eluant: 9:1 Cyclohexane/ethyl acetate) to afford 82.4 mg of **25** (80% yield, red brown solid, mp = 72-73 °C). Spectroscopic data were in accordance with literature data.[S20]

2-(3-Chlorophenyl)-1-phenylethan-1-one (26): From 91.9 mg (0.405 mmol) of **1l**, 800 μL (10 equiv.) of **ES2** and 64 mg of $NaHCO_3$ (0.1 mmol, 0.1 M) in 8 mL of degassed acetonitrile:water (9:1). Purification was carried out by silica gel chromatographic (eluant: 9:1 Cyclohexane/ethyl acetate) to afford 86.2 mg of **26** (89% yield, yellow solid, mp = 40-42 °C[S21]). Spectroscopic data were in accordance with literature data.[S13]

2-(2-Oxo-2-phenylethyl)benzonitrile (27): From 87.4 mg (0.418 mmol) of **1n**, 800 μL (10 equiv.) of **ES2** and 64 mg of $NaHCO_3$ (0.1 mmol, 0.1 M) in 8 mL of degassed acetonitrile:water (9:1). Purification was carried out by silica gel chromatographic column (eluant: 9:1 Cyclohexane ethyl acetate mixture) to afford 48.1 mg of **27** (52% yield, white solid, mp = 110-111 °C[S22]). Spectroscopic data were in accordance with literature data.[S23]

2-(2-Bromophenyl)-1-phenylethan-1-one (28): From 109.9 mg (0.420 mmol) of **1o**, 800 μL (10 equiv.) of **ES2** and 64 mg of $NaHCO_3$ (0.1 mmol, 0.1 M) in 8 mL of degassed acetonitrile:water (9:1). was carried out by silica gel chromatographic (eluant: 9:1 Cyclohexane/ethyl acetate) to afford 57.3

mg of **28** (52% yield, yellow solid, mp = 71-72 °C[S24]). Spectroscopic data were in accordance with literature data.[S25]

2-(Naphthalen-1-yl)-1-phenylethan-1-one (29): From 97.1 mg (0.415 mmol) of **1r**, 800 µL (10 equiv.) of **ES2** and 64 mg of $NaHCO_3$ (0.1 mmol, 0.1 M) in 8 mL of degassed acetonitrile:water (9:1). Purification was carried out by silica gel chromatographic column (eluant: 9:1 Cyclohexane/ethyl acetate) to afford 71.5 g of 29 (70% yield, red solid, mp = 106-107 °C[S19]). Spectroscopic data were in accordance with literature data.[S26]

2-(3-Benzoylphenyl)-1-phenylethan-1-one (30): From 0.112 mg (0.389 mmol) of **1q**, 800 µL (10 equiv.) of **ES2** and 64 mg of $NaHCO_3$ (0.1 mmol, 0.1 M) in 8 mL of degassed acetonitrile:water (9:1). Purification was carried out by silica gel chromatographic column (eluant: 9:1 Cyclohexane/ethyl acetate) to afford 35.0 mg of **30** (30% yield, yellow solid, mp = 101-102 °C). ^{1}H NMR (300 MHz, $CDCl_3$) δ 8.04 (s, 2H), 7.82 (d, *J* = 1.4 Hz, 4H), 7.59 (dd, *J* = 7.4, 3.0 Hz, 2H), 7.55 – 7.45 (m, 4H), 7.41 (d, *J* = 8.2 Hz, 2H), 4.40 (s, 2H). ^{13}C NMR (75 MHz, $CDCl_3$) δ 196.7, 196.2, 139.3, 137.5, 136.3, 136.1, 133.4, 132.3, 130.4, 130.0, 129.5, 128.7, 128.5, 128.2, 45.2. HRMS (EI) m/z: $[M]^+$ calcd for $C_{21}H_{16}O_2$ 301.1223, found 301.1216.

4-(2-Oxocyclohexyl)benzonitrile (31): From 80.9 mg (0.387 mmol) of **1a**, 800 µL (10 equiv.) of **ES3** and 64 mg of $NaHCO_3$ (0.1 mmol, 0.1 M) in 8 mL of degassed acetonitrile:water (9:1). Purification was carried out by silica gel chromatographic column (eluant: 9:1 Cyclohexane/ethyl acetate) to afford 30.0 mg of **31** (38% yield, slightly yellow solid, mp = 76-77 °C). ^{1}H NMR (300 MHz, $CDCl_3$) δ 7.63 (d, *J* = 8.3 Hz, 2H), 7.26 (d, *J* = 8.2 Hz, 2H), 3.69 (dd, *J* = 12.2, 5.3 Hz, 1H), 2.62–2.39 (m, 2H), 2.25 (d, *J* = 46.9 Hz, 2H), 2.13–1.77 (m, 4H). ^{13}C NMR (75 MHz, $CDCl_3$) δ 208.8, 144.1, 132.0, 129.4, 118.8, 110.7, 57.3, 42.1, 35.0, 27.6, 25.2. HRMS (EI) m/z: $[M]^+$ calcd for $C_{13}H_{13}NO$ 200.1070, found 200.1065.

2-(4-Chlorophenyl)cyclohexan-1-one (32): From 91.5 mg (0.419 mmol) of **1c**, 800 µL (10 equiv.) of **ES3** and 64 mg of $NaHCO_3$ (0.1 mmol, 0.1 M) in 8 mL of degassed acetonitrile:water (9:1). Purification step carried out by silica gel chromatographic column (eluant: 9:1 Cyclohexane/ethyl acetate) to afford 30.3 mg of **32** (36% yield, slightly yellow solid, mp = 83-84°C[S27]). Spectroscopic data were in accordance with literature data.[S28]

2-(4-Bromophenyl)cyclohexan-1-one (33): From 104.2 mg (0.397 mmol) of **1d**, 800 µL (10 equiv.) of **ES3** and 64 mg of $NaHCO_3$ (0.1 mmol, 0.1 M) in 8 mL of degassed acetonitrile:water (9:1). Purification was carried out by silica gel chromatographic column (eluant: 9:1 Cyclohexane/ethyl

acetate) to afford 94.4 mg of **33** (22% yield, red brown solid, mp = 84-85 °C[S29]). Spectroscopic data were in accordance with literature data.[S28]

2-(4-Fluorophenyl)cyclopentan-1-one (34): From 80.7 mg (0.413 mmol) of **1b**, 720 µL (10 equiv.) of **ES4** and 64 mg of $NaHCO_3$ (0.1 mmol, 0.1 M) in 8 mL of degassed acetonitrile:water (9:1). Purification was carried out by silica gel chromatographic column (eluant: 95:5 Cyclohexane/ethyl acetate) to afford 34.3 mg of **34** (43% yield, red oil). Spectroscopic data were in accordance with literature data.[S30]

2-(4-Chlorophenyl)cyclopentan-1-one (35): From 107.1 mg (0.491 mmol) of **1c** and 720 µL of **ES4**. Purification was carried out by silica gel chromatographic column (eluant: 95:5 Cyclohexane/ethyl acetate) to afford 31.6 mg of **35** (42% yield, red oil). Spectroscopic data were in accordance with literature data.[S31]

2-(4-Acetylphenyl)cyclopentan-1-one (36): From 94.7 mg (0.419 mmol) of **1f**, 720 µL (10 equiv.) of **ES4** and 64 mg of $NaHCO_3$ (0.1 mmol, 0.1 M) in 8 mL of degassed acetonitrile:water (9:1). Purification was carried out by silica gel chromatographic column (eluant: 95:5 Cyclohexane/ethyl acetate) to afford 45.7 mg of **36** (54% yield, yellow oil). Spectroscopic data were in accordance with literature data.[S32]

2-(4-(Trifluoromethyl)phenyl)cyclopentan-1-one (37): From 105.1 mg (0.417 mmol) of **1h**, 720 µL (10 equiv.) of **ES4** and 64 mg of $NaHCO_3$ (0.1 mmol, 0.1 M) in 8 mL of degassed acetonitrile:water (9:1). Purification was carried out by silica gel chromatographic column (eluant: 95:5 Cyclohexane/ethyl acetate) to afford 70.4 mg of **37** (74% yield, yellow oil). Spectroscopic data were in accordance with literature data.[S30]

3-(2-Oxocyclopentyl)benzonitrile (38): From 83.2 mg (0.398 mmol) of **1k**, 720 µL (10 equiv.) of **ES4** and 64 mg of $NaHCO_3$ (0.1 mmol, 0.1 M) in 8 mL of degassed acetonitrile:water (9:1). Purification was carried out by silica gel chromatographic column (eluant: 95:5 Cyclohexane/ethyl acetate) to afford 43.4 mg of **38** (59% yield, slightly yellow oil). Spectroscopic data were in accordance with literature data.[S30]

2-(3-Chlorophenyl)cyclopentan-1-one (39): From 83.5 mg (0.383 mmol) of **1l**, 720 µL (10 equiv.) of **ES4** and 64 mg of $NaHCO_3$ (0.1 mmol, 0.1 M) in 8 mL of degassed acetonitrile:water (9:1). Purification was carried out by silica gel chromatographic column (eluant: 95:5 Cyclohexane/ethyl acetate) to afford 42.4 mg of **39** (57% yield, yellow oil). Spectroscopic data were in accordance with literature data.[S30]

2-(2-Bromophenyl)cyclopentan-1-one (40): From 103.4 mg (0.395 mmol) of **1o**, 720 µL (10 equiv.) of **ES4** and 64 mg of $NaHCO_3$ (0.1 mmol, 0.1 M) in 8 mL of degassed acetonitrile:water (9:1). Purification was carried out by silica gel chromatographic column (eluant: 95:5 Cyclohexane/ethyl acetate) to afford 76.0 mg of **40** (81% yield, slightly yellow oil). Spectroscopic data were in accordance with literature data.[S33]

2-(2-Methoxyphenyl)cyclopentan-1-one (41): From 80.9 g (0.378 mmol) of **1p**, 720 µL (10 equiv.) of **ES4** and 64 mg of $NaHCO_3$ (0.1 mmol, 0.1 M) in 8 mL of degassed acetonitrile:water (9:1). Purification was carried out by silica gel chromatographic column (eluant: 95:5 Cyclohexane/ethyl acetate) to afford 58.2 mg of **41** (81% yield, red solid, mp = 107-108 °C[S34]). Spectroscopic data were in accordance with literature data.[S33]

Methyl 2-((4-cyanophenyl)diazenyl)-2-methylpropanoate (42): From 86.7 mg (0.415 mmol) of **1a**, 740 µL (10 equiv.) of **ES5** and 64 mg of $NaHCO_3$ (0.1 mmol, 0.1 M) in 8 mL of degassed acetonitrile:water (9:1). Purification was carried out by silica gel chromatographic column (eluant: 9:1 Cyclohexane/ethyl acetate) to afford 83.4 mg of **42** (87% yield, yellow oil). ^1H NMR (300 MHz, $CDCl_3$) δ 7.77 (s, 4H), 3.77 (s, 3H), 1.61 (s, 6H). ^{13}C NMR (75 MHz, $CDCl_3$) δ 173.1, 153.5, 133.0, 132.1, 126.6, 122.9, 118.1, 114.0, 76.3, 52.3, 23.0. HRMS (EI) m/z: $[M]^+$ calcd for $C_{12}H_{13}N_3O_2$ 232.1081, found 232.1071.

Methyl 2-((4-fluorophenyl)diazinyl)-2-methylpropanoate (43): From 81.4 mg (0.403 mmol) of **1b**, 740 µL (10 equiv.) of **ES5** and 64 mg of $NaHCO_3$ (0.1 mmol, 0.1 M) in 8 mL of degassed acetonitrile:water (9:1). Purification was carried out by silica gel chromatographic column (eluant: 9:1 Cyclohexane/ethyl acetate) to afford 74.9 mg of **43** (83% yield, slightly yellow oil). ^1H NMR (300 MHz, $CDCl_3$) δ 7.86 – 7.62 (m, 2H), 7.15 (t, J = 8.6 Hz, 2H), 3.77 (s, 3H), 1.60 (s, 6H). ^{13}C NMR (75 MHz, $CDCl_3$) δ 173.7, 165.82, 162.49, 124.4-124.3 (d, $J_{(C\text{-}F)}$= 8.9 Hz), 115.9-15.6 (d, $J_{(C\text{-}F)}$= 22.7 Hz), 75.3, 52.1, 23.0. HRMS (EI) m/z: $[M]^+$ calcd for $C_{11}H_{13}N_2O_2F$ 225.1034, found 225.1033.

Methyl 2-(4-chlorophenyl)-2-methylpropanoate (44): From 91.4 mg (0.419 mmol) of **1c**, 740 µL (10 equiv.) of **ES5** and 64 mg of $NaHCO_3$ (0.1 mmol, 0.1 M) in 8 mL of degassed acetonitrile:water (9:1). Purification was carried out by silica gel chromatographic column (eluant: 9:1 Cyclohexane/ethyl acetate) to afford 30.3 g of **44** (33% yield, yellow oil). ^1H NMR (300 MHz, $CDCl_3$) δ 7.67 (d, J = 8.7 Hz, 2H), 7.44 (d, J = 8.7 Hz, 2H), 3.77 (s, 3H), 1.60 (s, 6H). ^{13}C NMR (75 MHz, $CDCl_3$) δ 173.7, 149.9, 136.7, 129.1, 123.6, 75.5, 52.2, 23.0. HRMS (EI) m/z: $[M]^+$ calcd for $C_{11}H_{13}N_2O_2Cl$ 241.0738, found 241.0730.

Methyl 2-methyl-2-((4-(trifluoromethyl)phenyl)diazenyl)propanoate (45): From 104.8 mg (0.416 mmol) of **1h**, 740 µL (10 equiv.) of **ES5** and 64 mg of $NaHCO_3$ (0.1 mmol, 0.1 M) in 8 mL of degassed acetonitrile:water (9:1). Purification was carried out by silica gel chromatographic column (eluant: 9:1 Cyclohexane/ethyl acetate mixture) to afford 92.4 mg of **45** (81% yield, yellow oil). ^1H NMR (300 MHz, $CDCl_3$) δ 7.75 (q, J = 8.6 Hz, 4H), 3.76 (s, 3H), 1.61 (s, 6H). ^{13}C NMR (75 MHz, DMSO-d_6) δ 173.4, 153.5, 129.5, 128.9 (d, $J_{(C-F)}$= 96.0 Hz), 126.1 (q, $J_{(C-F)}$= 3.7 Hz), 122.5, 120 (d, $J_{(C-F)}$ = 289.3 Hz), 118.1, 76.1, 52.2, 23.0. HRMS (EI) m/z: $[M]^+$ calcd for $C_{12}H_{13}N_2O_2F_3$ 275.1002, found 275.0990.

Methyl 2-methyl-2-(p-tolyldiazenyl)propanoate (46): From 75.6 mg (0.391 mmol) of **1i**, 740 µL (10 equiv.) of **ES5** and 64 mg of $NaHCO_3$ (0.1 mmol, 0.1 M) in 8 mL of degassed acetonitrile:water (9:1). Purification was carried out by silica gel chromatographic column (eluant: 9:1 Cyclohexane/ethyl acetate) to afford 28.4 mg of **46** (33% yield, yellow oil). Spectroscopic data were in accordance with literature data.[S35]

Methyl 2-((3-benzoylphenyl)diazenyl)-2-methylpropanoate (47): From 0.109.1 mg (0.389 mmol) of **1q**, 740 µL (10 equiv.) of **ES5** and 64 mg of $NaHCO_3$ (0.1 mmol, 0.1 M) in 8 mL of degassed acetonitrile:water (9:1). Purification was carried out by silica gel chromatographic column (eluant: 9:1 Cyclohexane/ethyl acetate) to afford 65.6 mg of **47** (61% yield, yellow oil). ^1H NMR (300 MHz, $CDCl_3$) δ 7.91 (d, J = 8.5 Hz, 2H), 7.85 – 7.74 (m, 4H), 7.63 (t, J = 7.4 Hz, 1H), 7.51 (t, J = 7.5 Hz, 2H), 3.79 (s, 3H), 1.64 (s, 6H). ^{13}C NMR (75 MHz, $CDCl_3$) δ 195.8, 173.5, 132.6, 130.8, 129.9, 128.3, 122.0, 122.0, 76.1, 52.2, 23.0. HRMS (EI) m/z: $[M]^+$ calcd for $C_{18}H_{18}N_2O_3$ 311.1390, found 311.1375.

Sunlight-driven synthesis of compound 12.

SCHEME S1: Sunlight driven photochemical synthesis of **12** starting from **ES1** and **1k**.

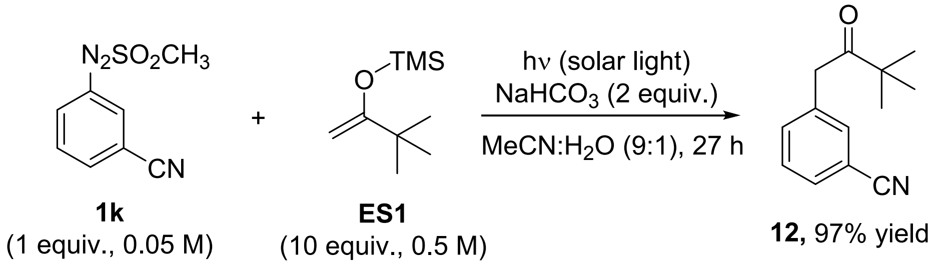

A glass vessel was charged with 91.2 mg of arylazo sulfone **1k** (0.436 mmol, 1.0 equiv., 0.05 M) and 64 mg of sodium bicarbonate (0.1 mmol, 2.0 equiv., 0.1 M) was flushed with argon and the solid was dissolved in degassed acetonitrile:water (9:1, 8.0 mL). Then, 800 µL of enol silyl ether **ES1** were added (4.0 mmol, 10.0 equiv.., 0.5 M). The glass vessel was put outside the laboratory window on an aluminium foil and was exposed to natural sunlight for 27 h (May 2021, Pavia, Italy, coordinates: 45° 11' 7" 44 **N** 09° 9' 45" 00 **E**, see Figure S2)**.** The reaction course was monitored through GC analysis. The photolyzed solution was concentrated under reduced pressure and the crude mixture obtained was purified by silica gel column chromatography (9:1 cyclohexane/ethyl acetate) affording 84.6 mg of **12** (97% yield, slightly yellow solid).

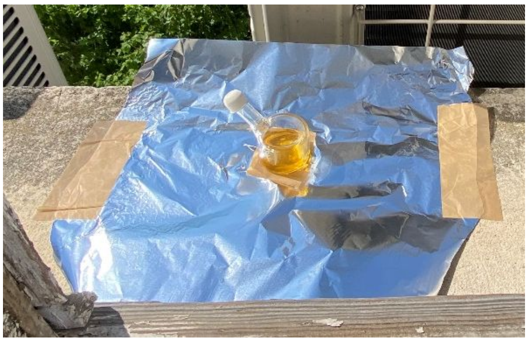

Figure S2: The reaction vessel containing the arylazo sulfone **1k** and the enol silyl ether **ES1** was put outside the laboratory window and exposed to solar light for three days in a row (9 h of light exposure every day). An aluminium foil was put under the reaction vessel.

2. Copy of the ^{1}H and ^{13}C NMR of compounds 2-47

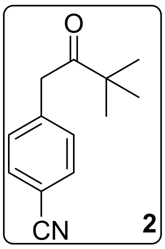

^{1}H NMR (300 MHz, $CDCl_3$)

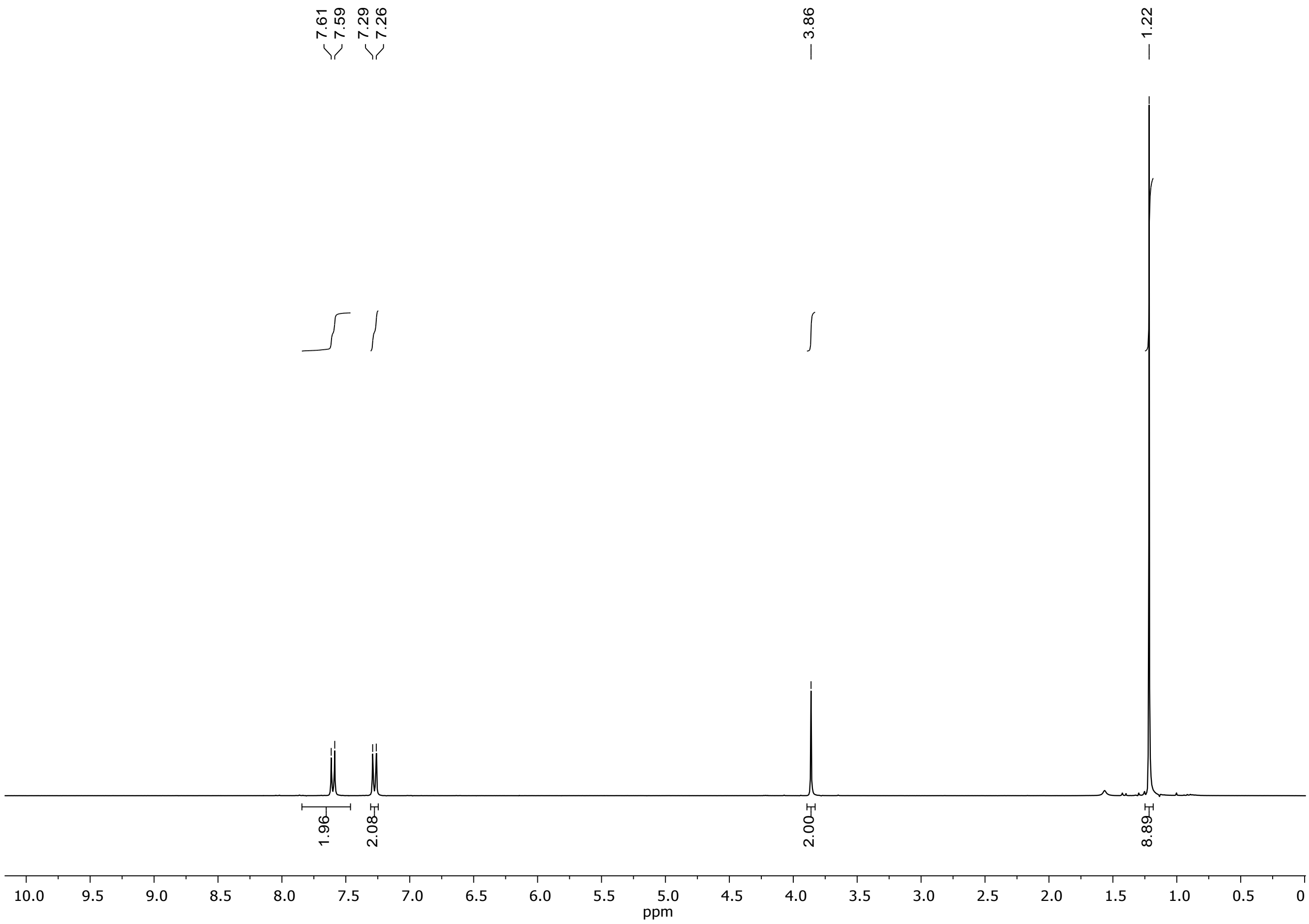

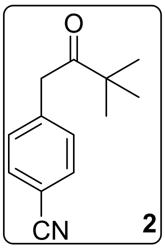

2

^{13}C NMR (75 MHz, $CDCl_3$)

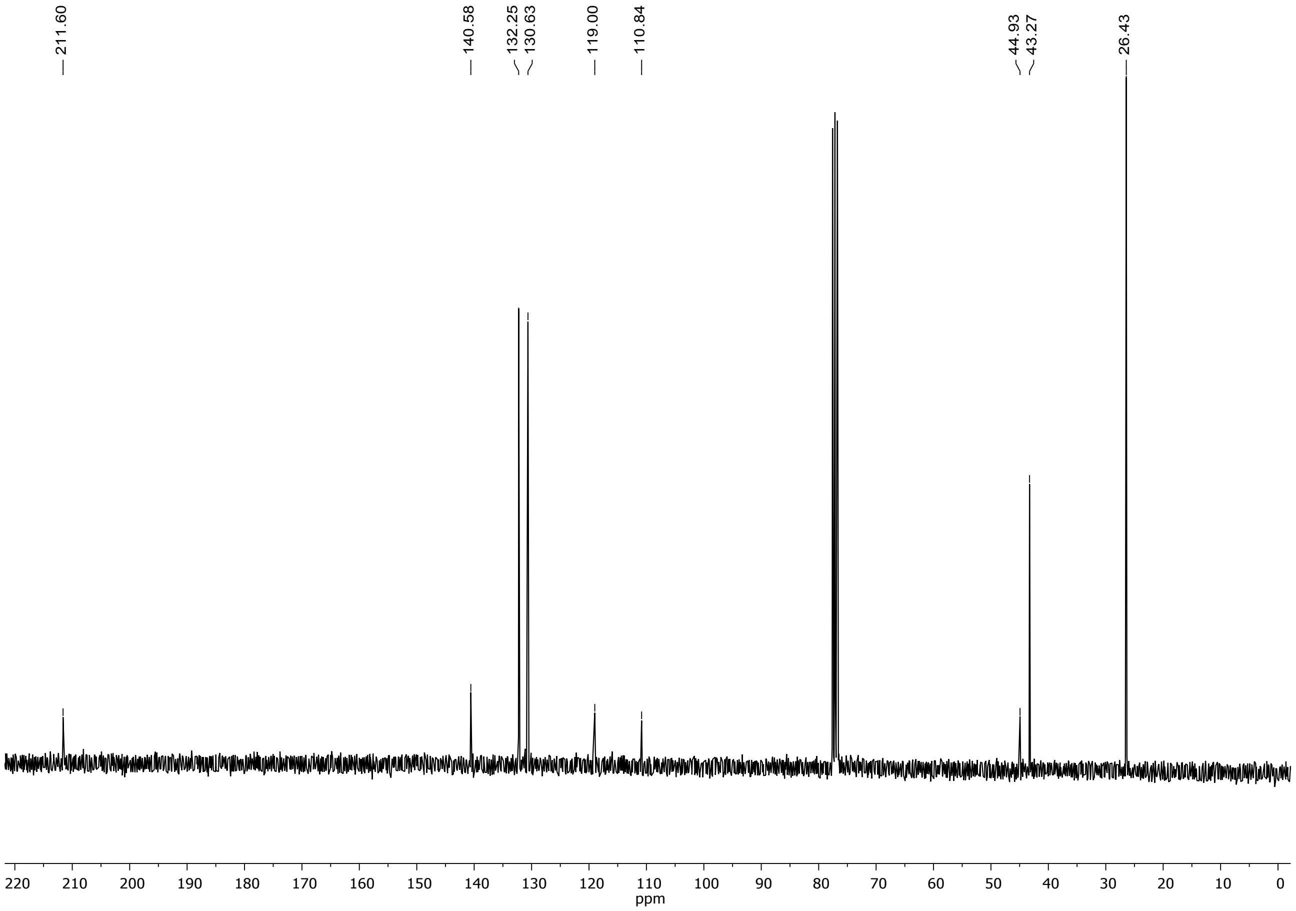

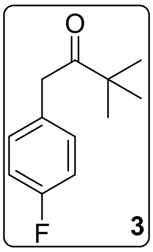

3 ^1H NMR (300 MHz, $CDCl_3$)

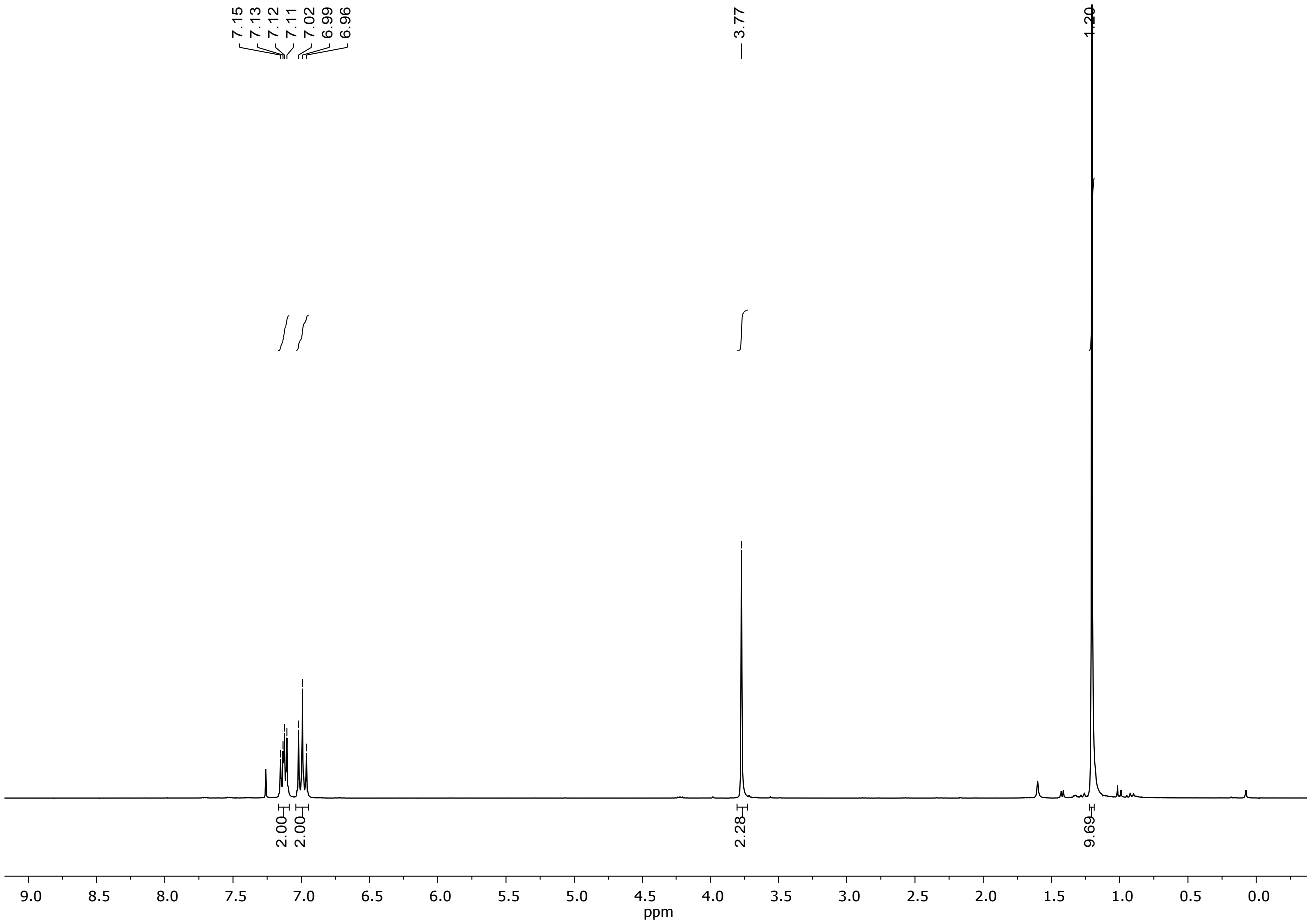

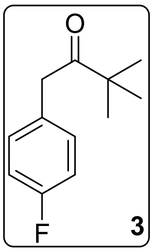

3 ^{13}C NMR (75 MHz, $CDCl_3$)

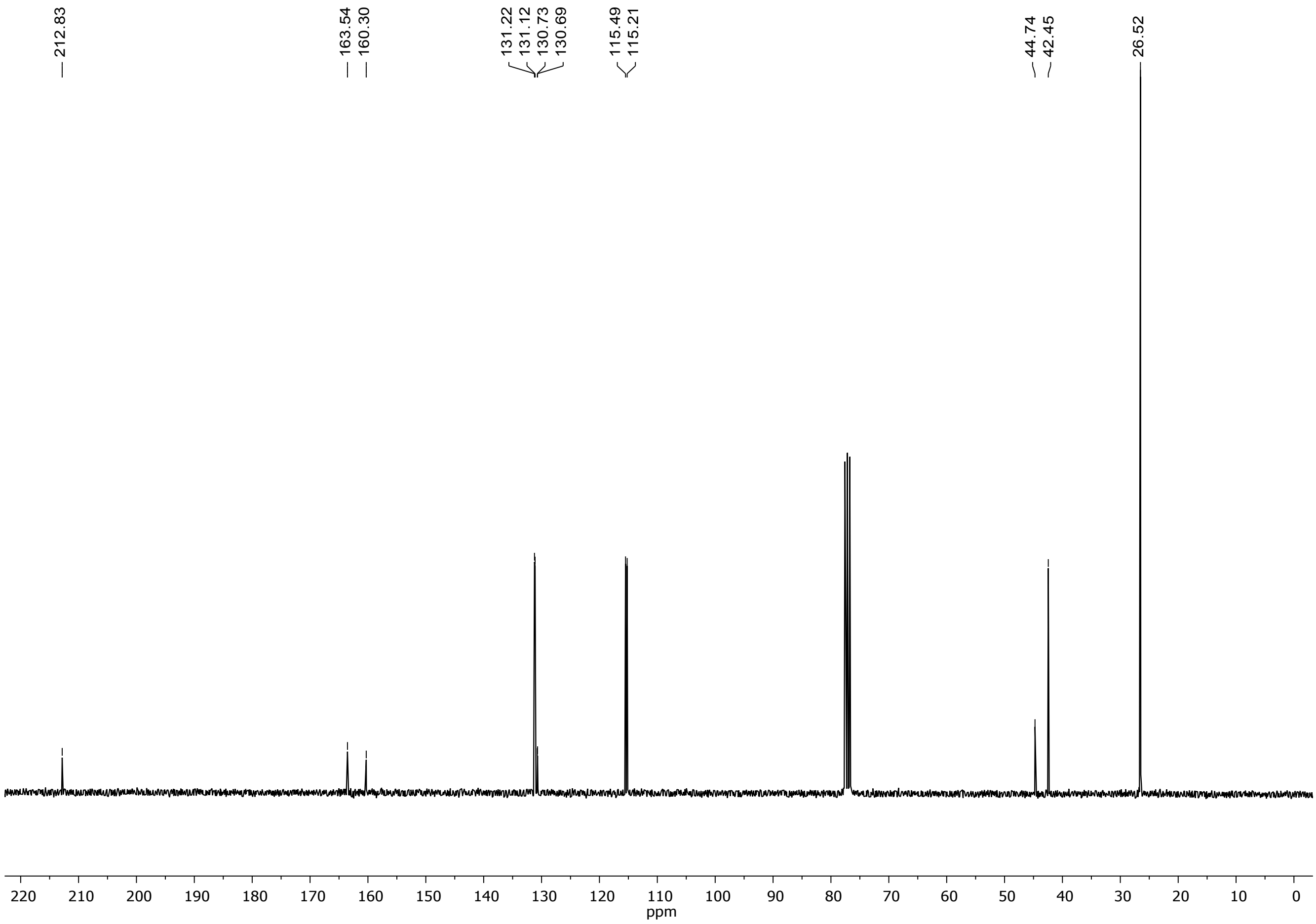

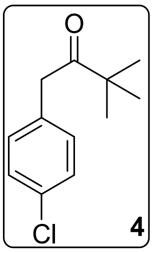

4 ^{1}H NMR (300 MHz, $CDCl_3$)

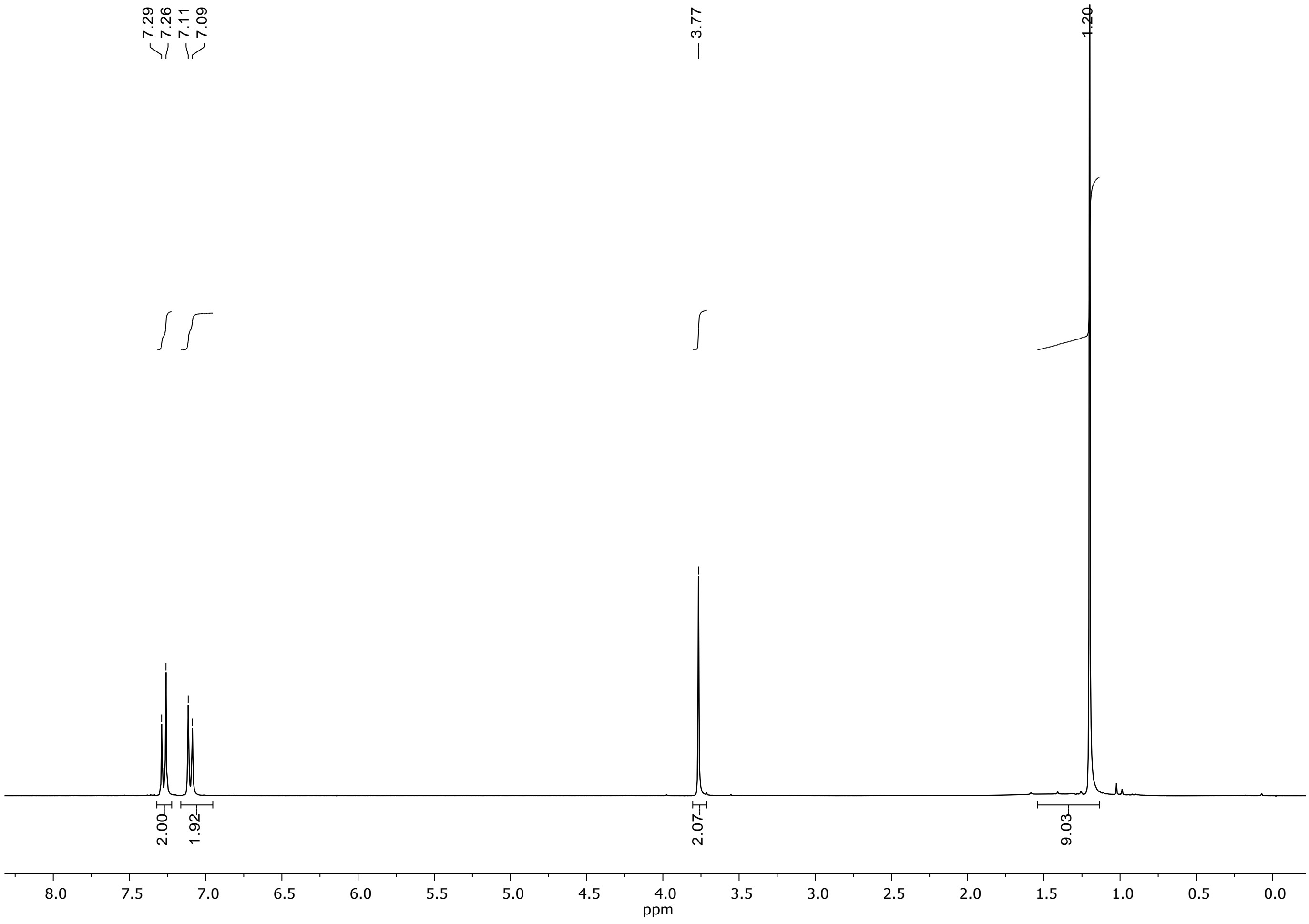

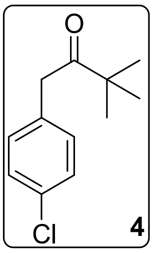

^{13}C NMR (75 MHz, $CDCl_3$)

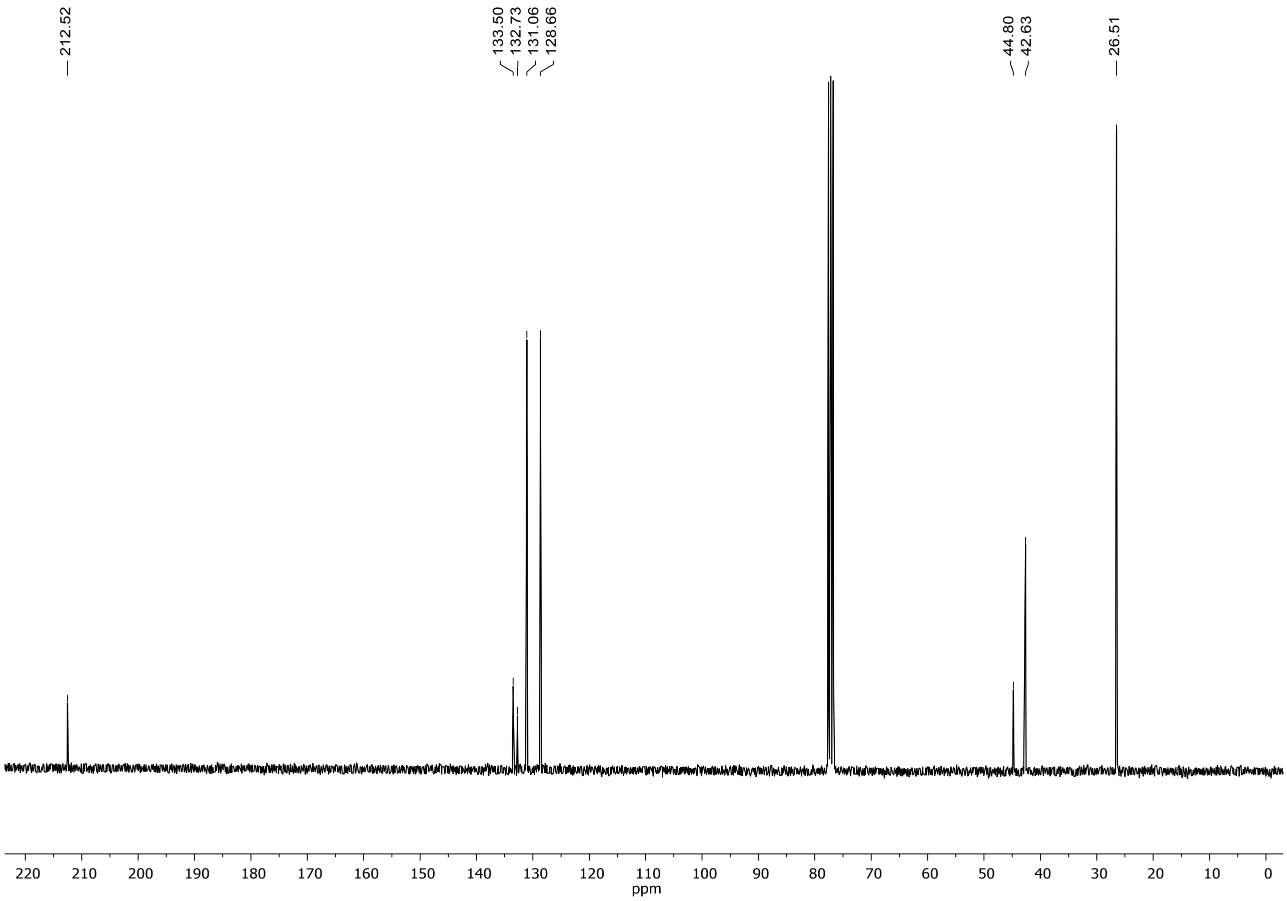

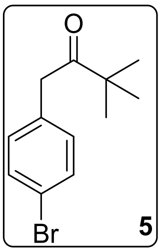

5 ^{1}H NMR (300 MHz, $CDCl_3$)

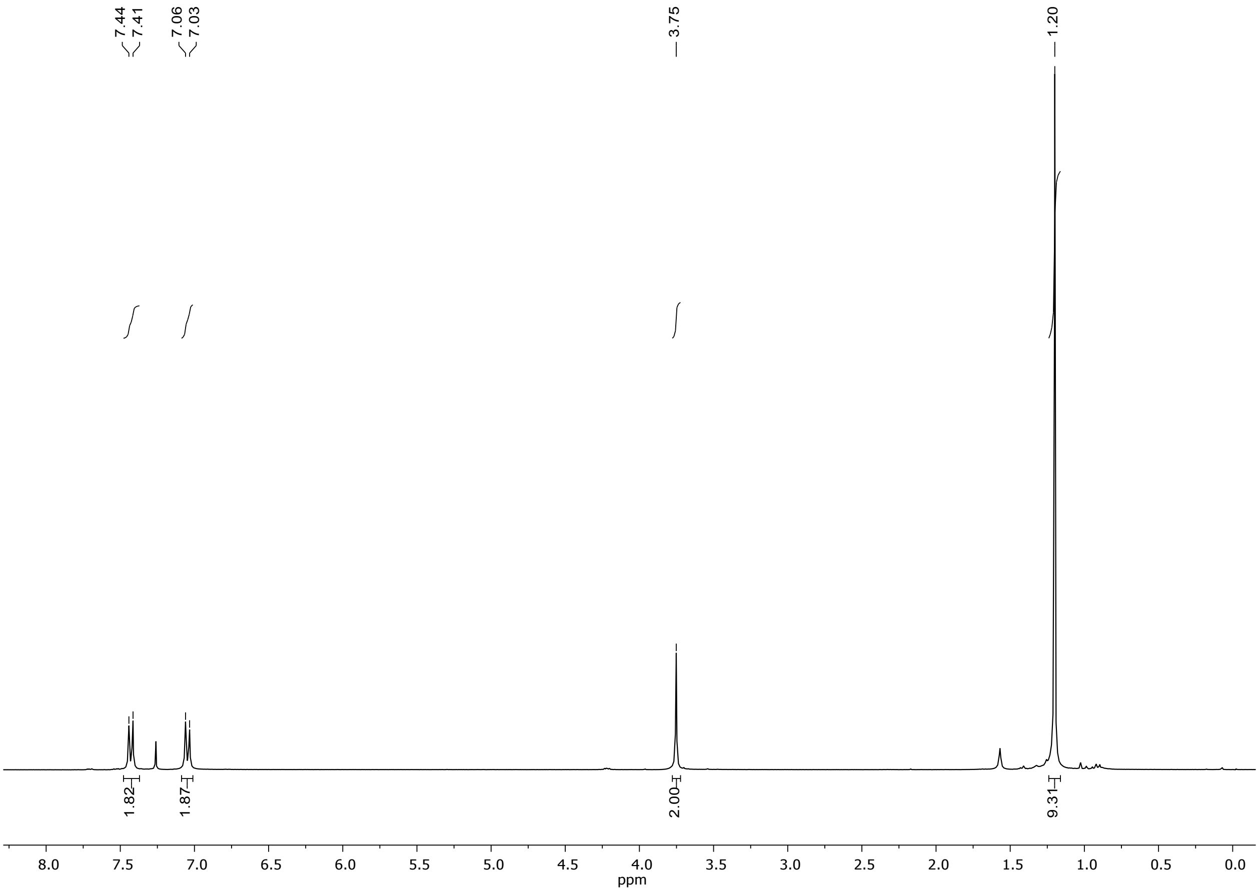

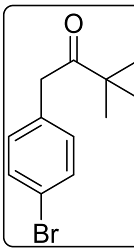

5 ^{13}C NMR (75 MHz, $CDCl_3$)

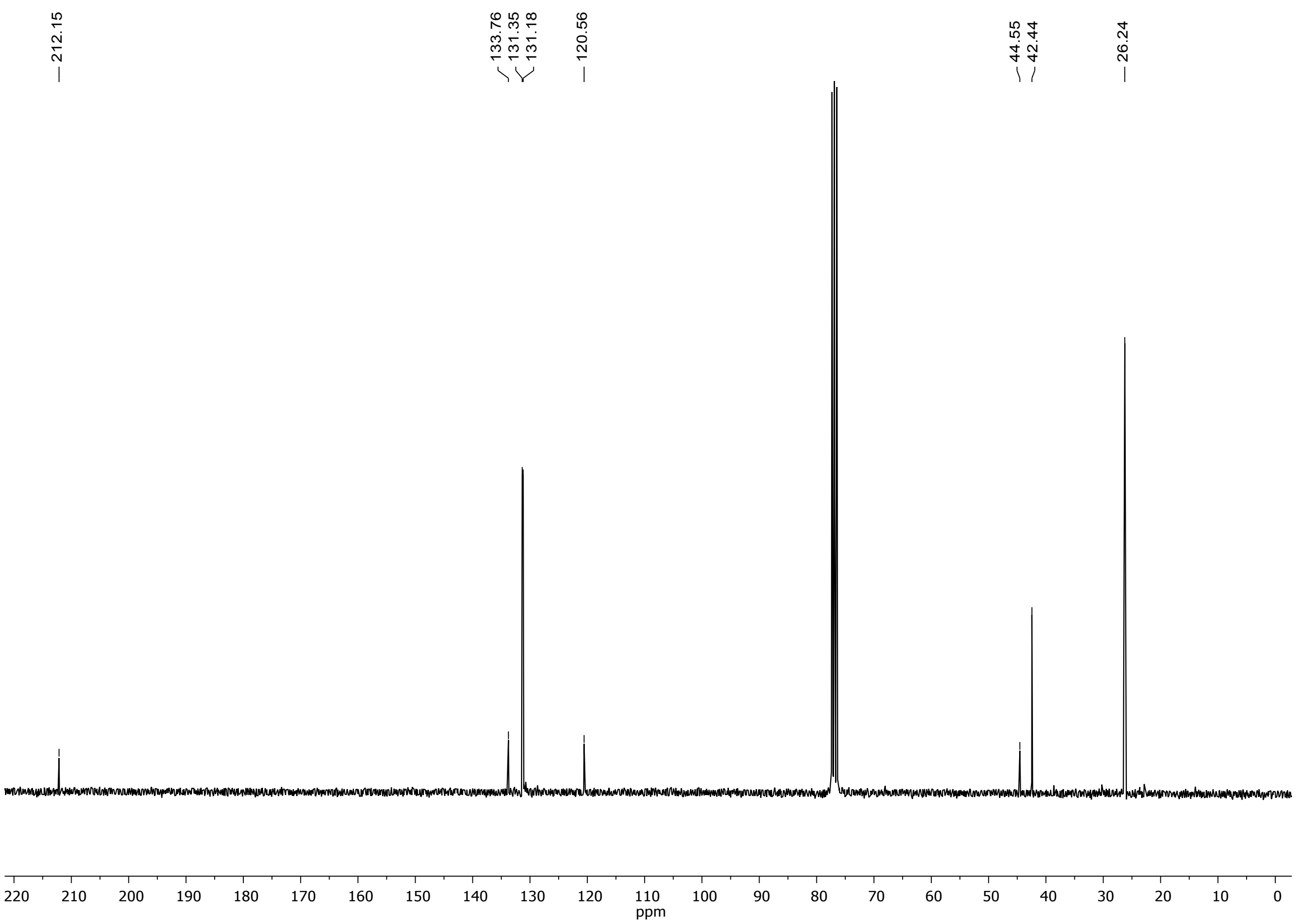

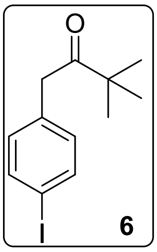

6

^{1}H NMR (300 MHz, $CDCl_3$)

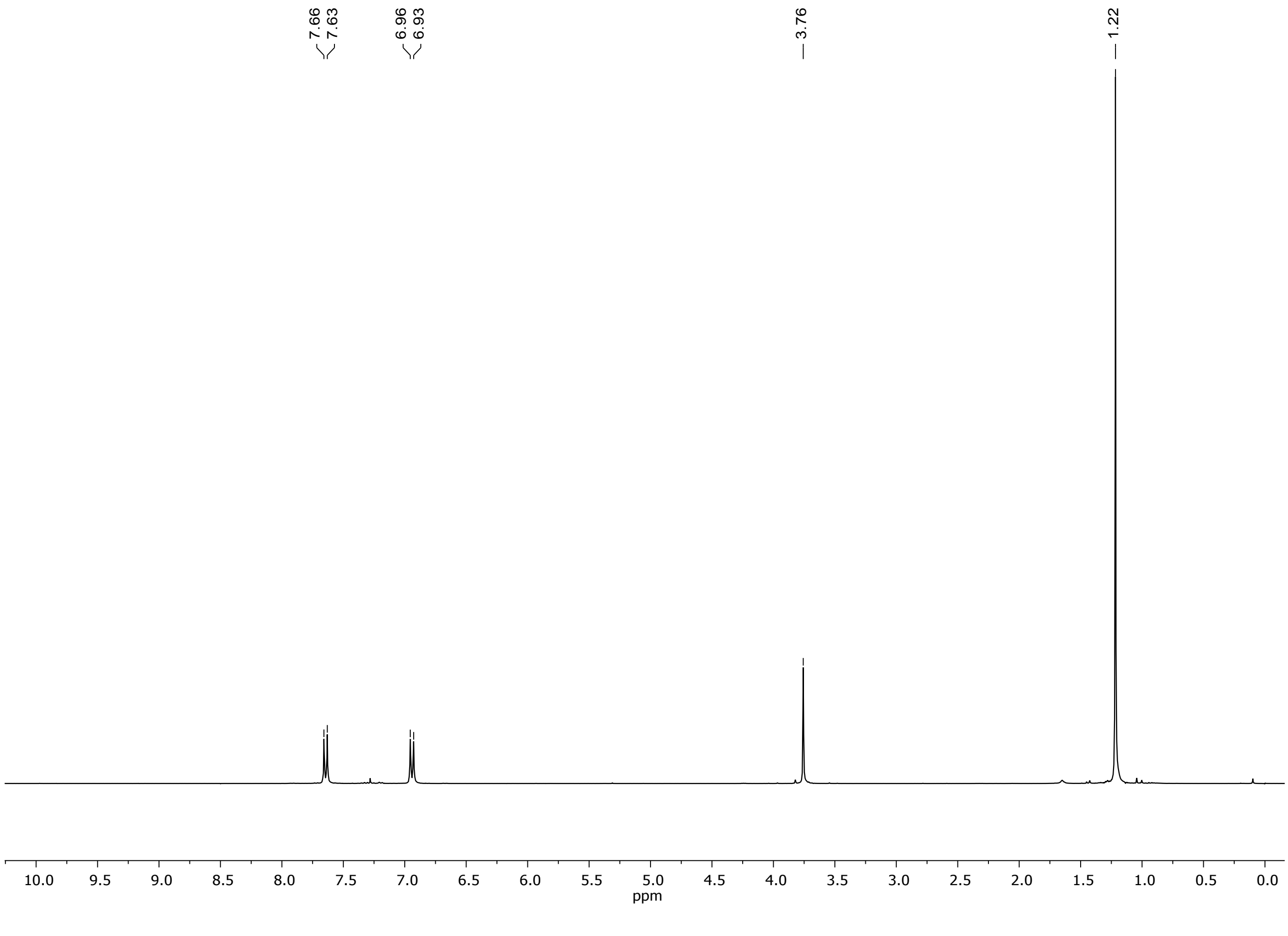

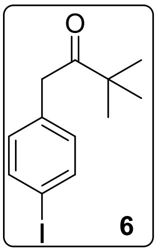

6 ^{13}C NMR (75 MHz, $CDCl_3$)

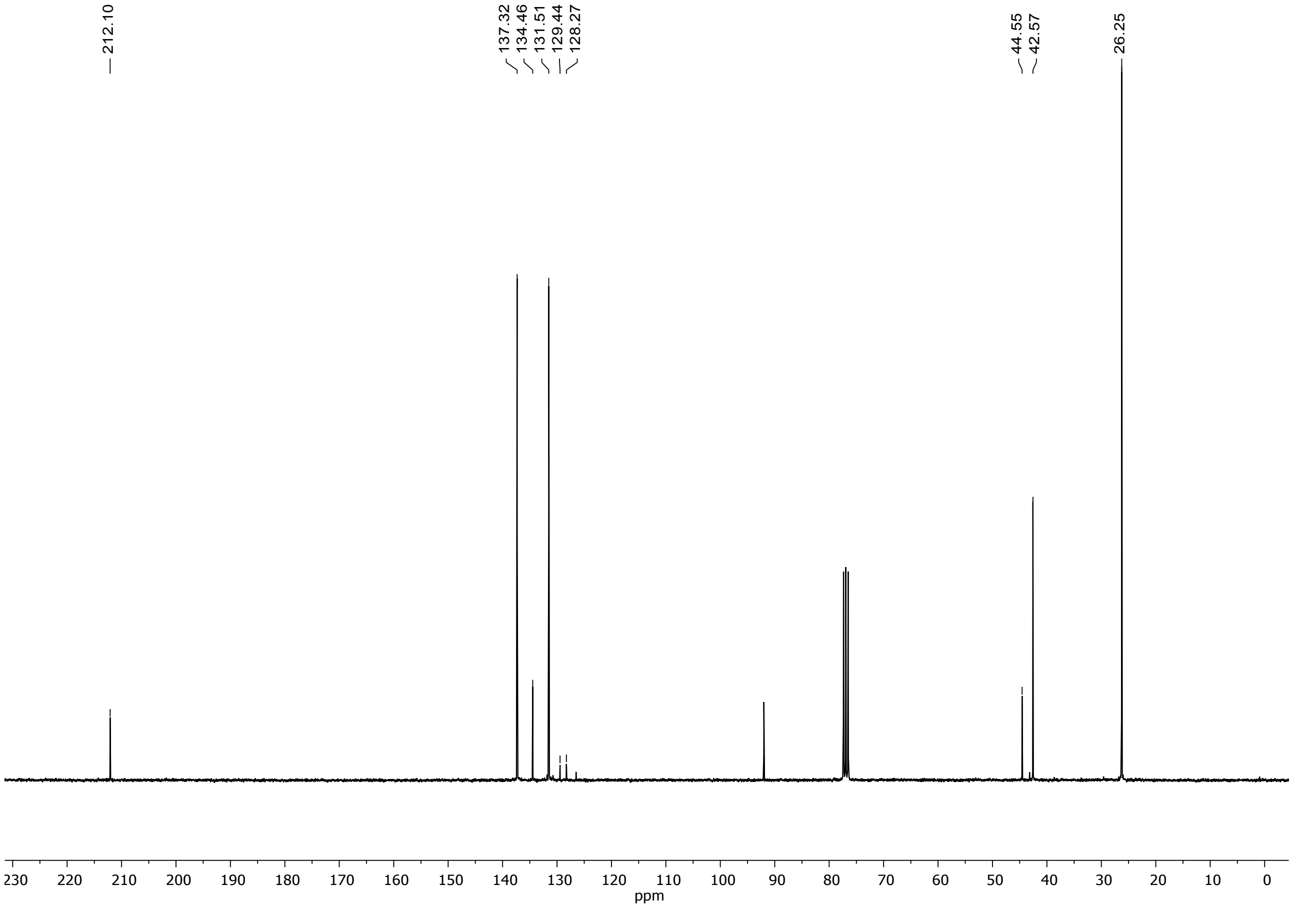

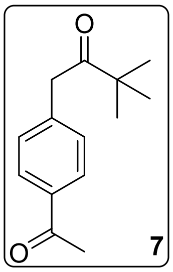

7

^{1}H NMR (300 MHz, $CDCl_3$)

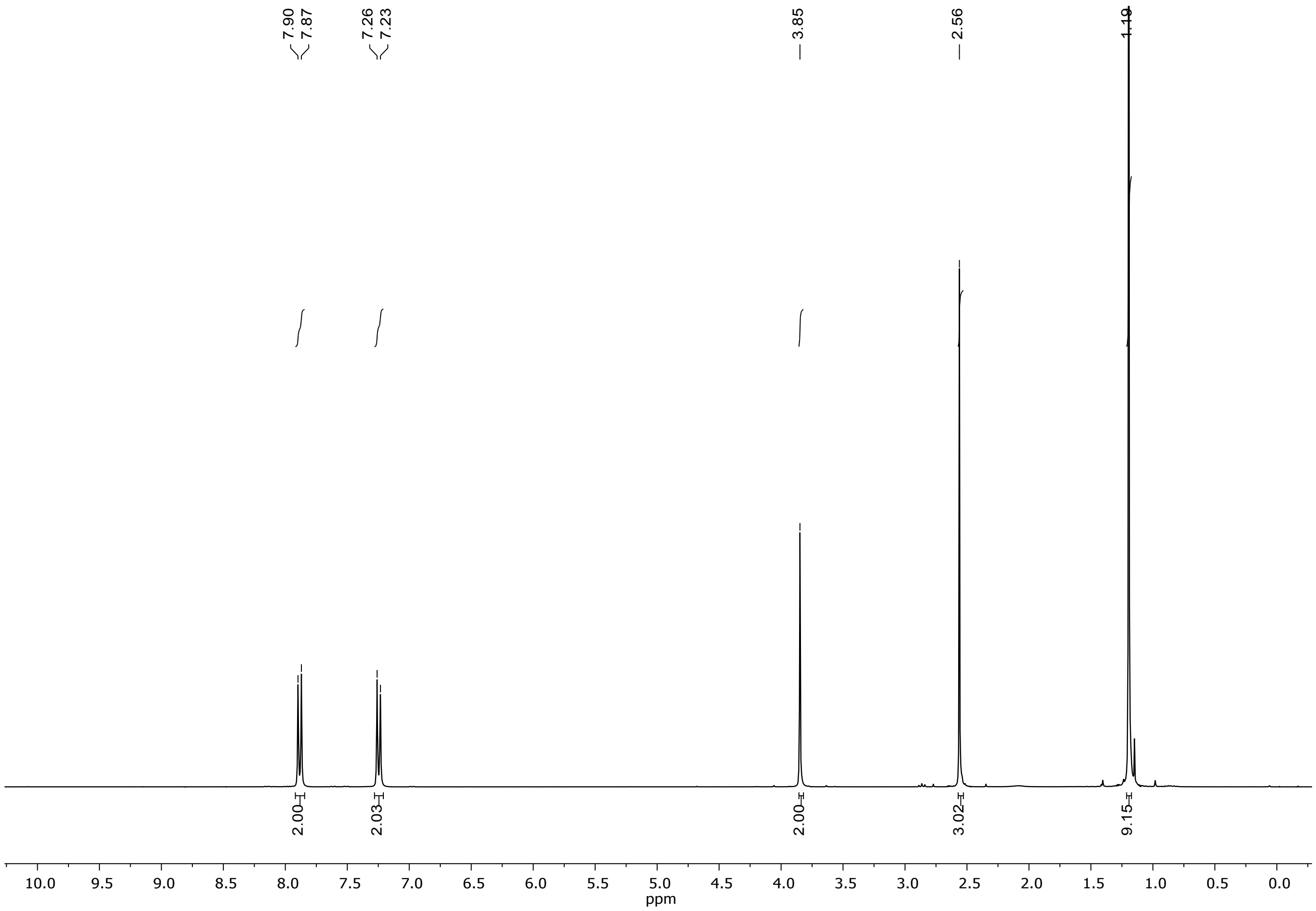

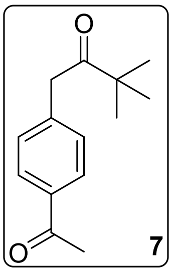

7

^{13}C NMR (75 MHz, $CDCl_3$)

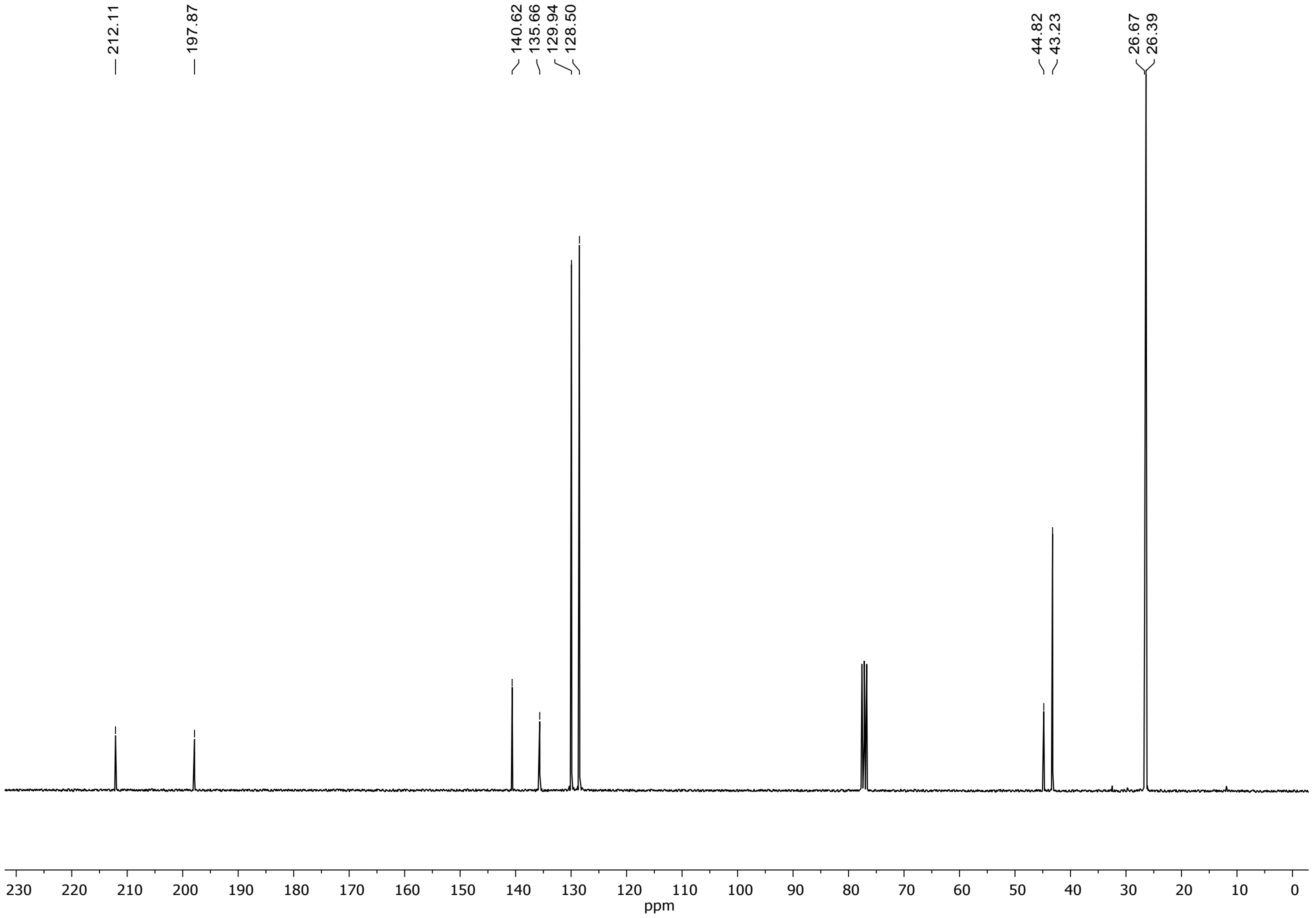

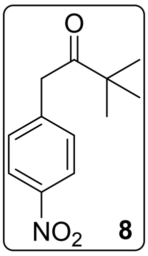

8

^{1}H NMR (300 MHz, $CDCl_3$)

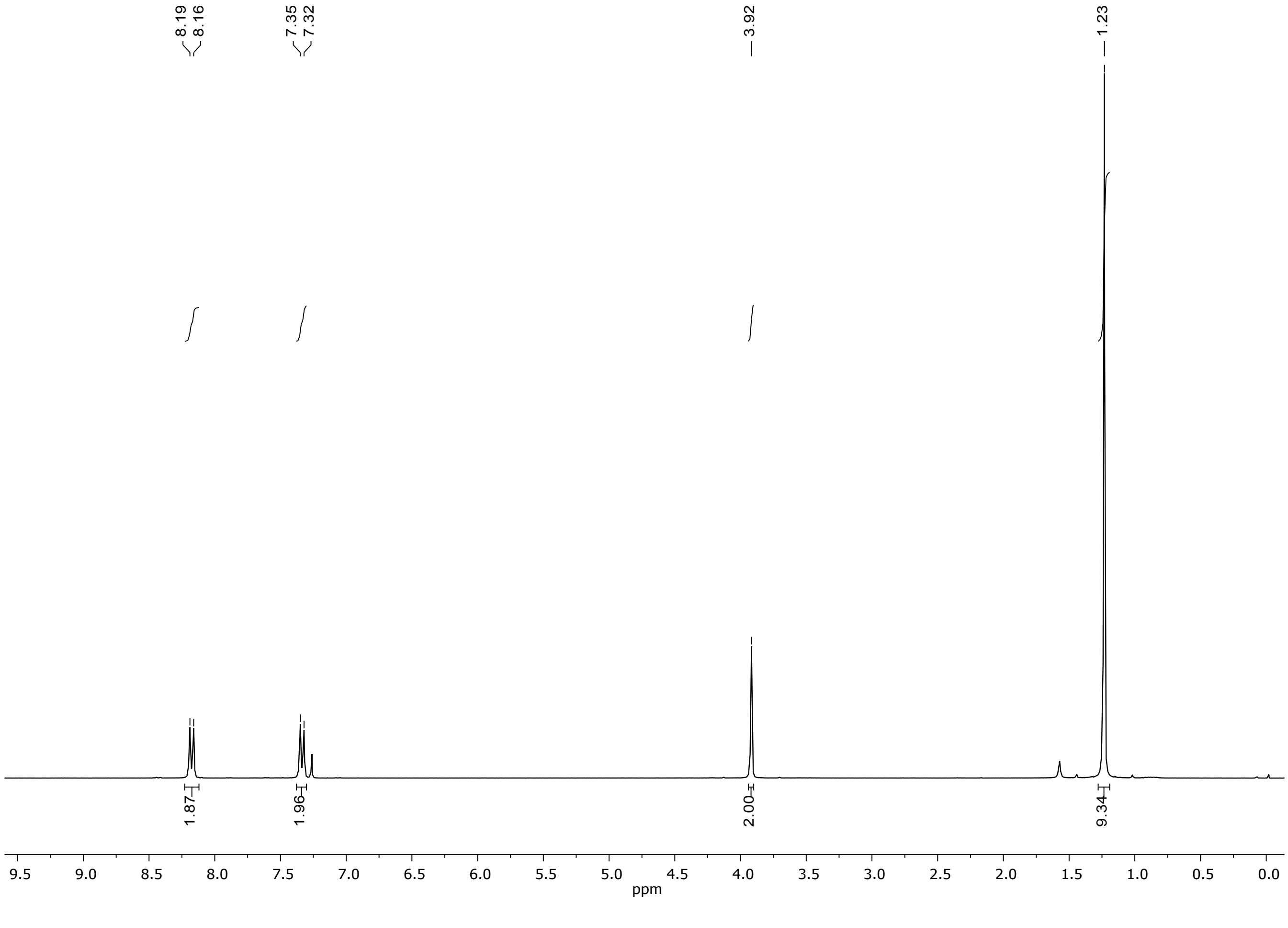

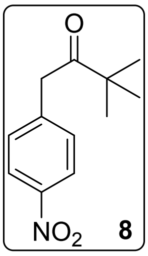

^{13}C NMR (75 MHz, $CDCl_3$)

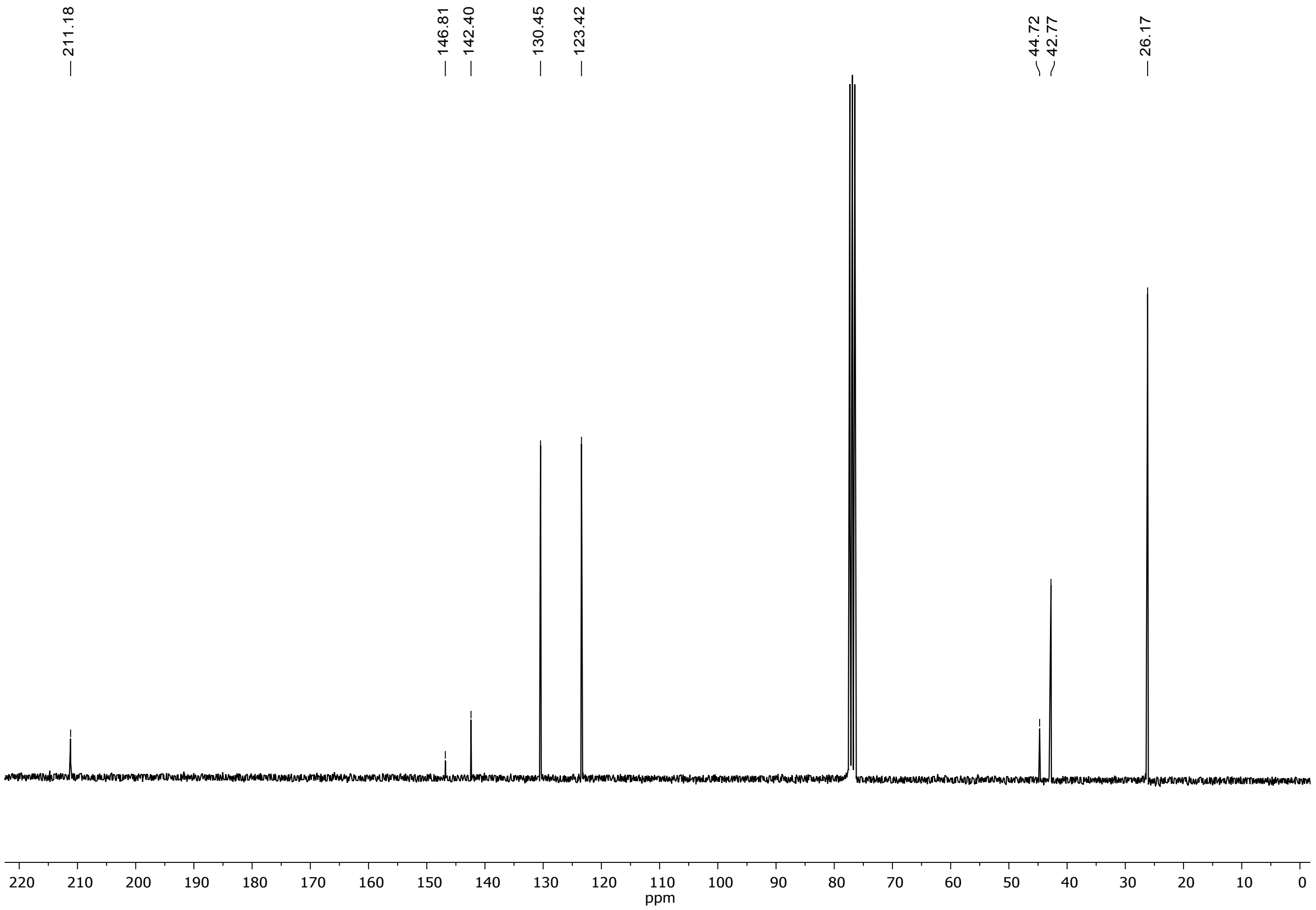

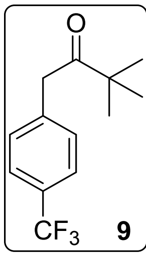

9

^{1}H NMR (300 MHz, $CDCl_3$)

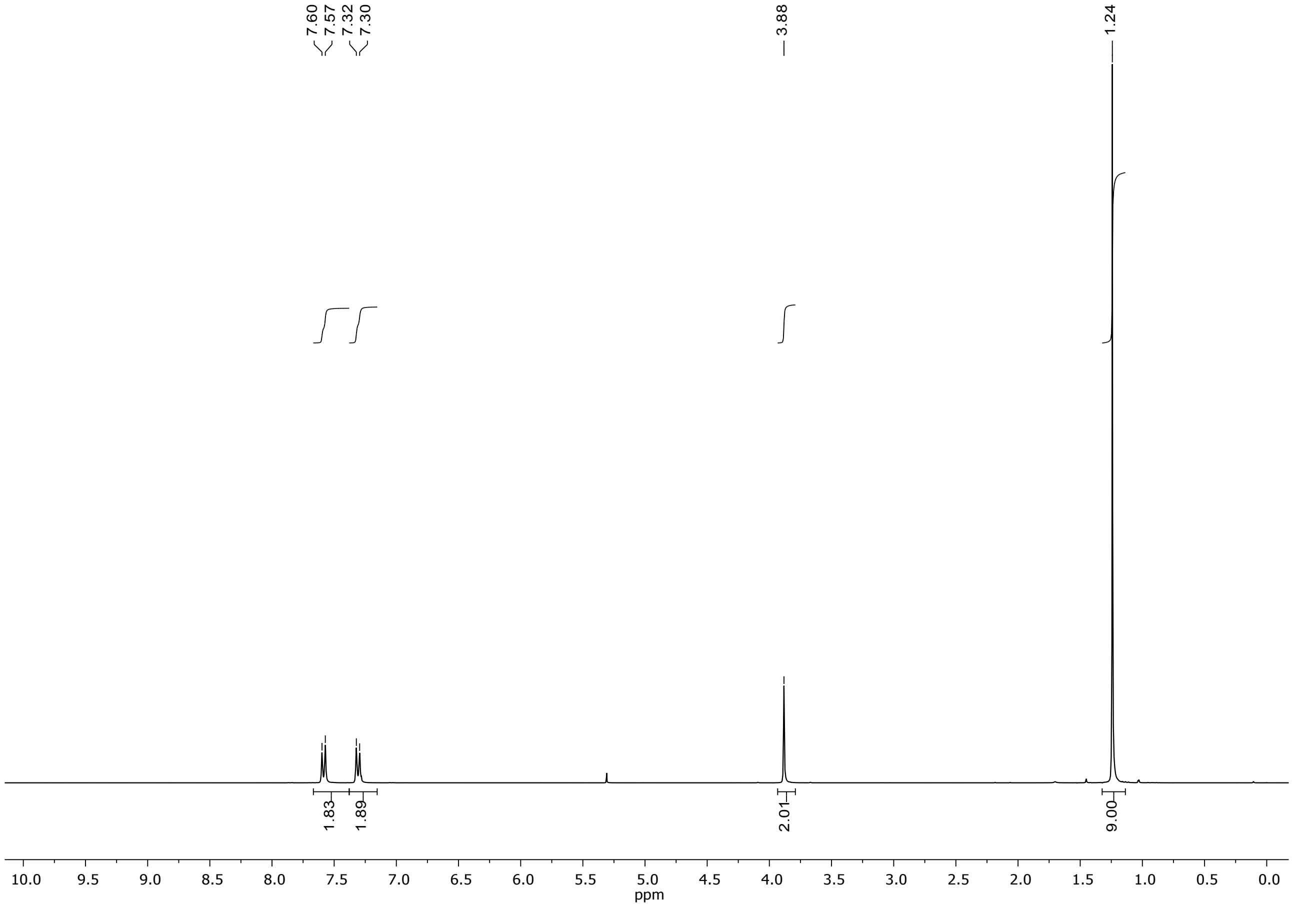

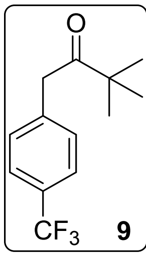

9 ^{13}C NMR (75 MHz, $CDCl_3$)

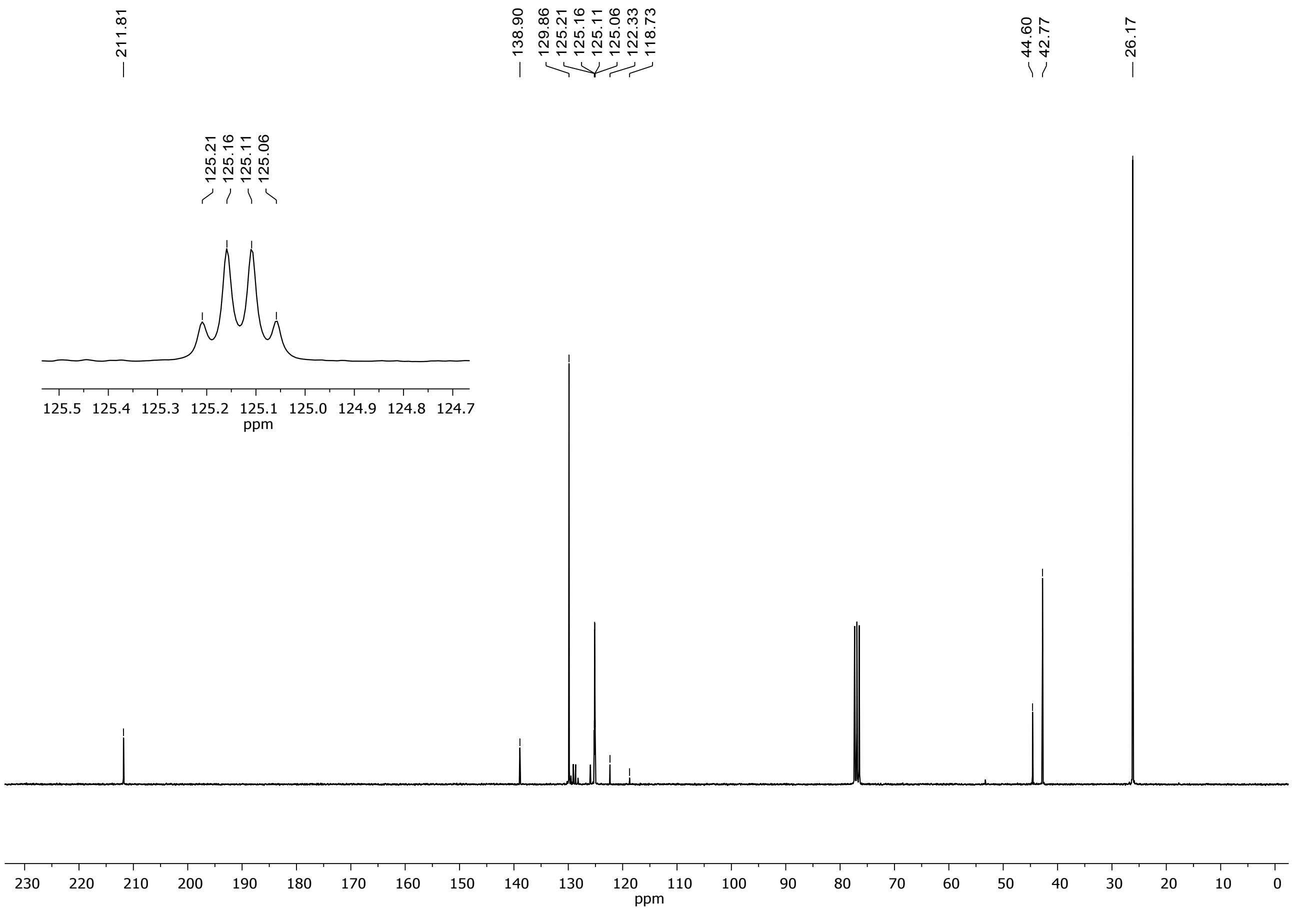

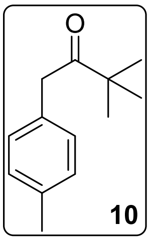
10

^{1}H NMR (300 MHz, $CDCl_3$)

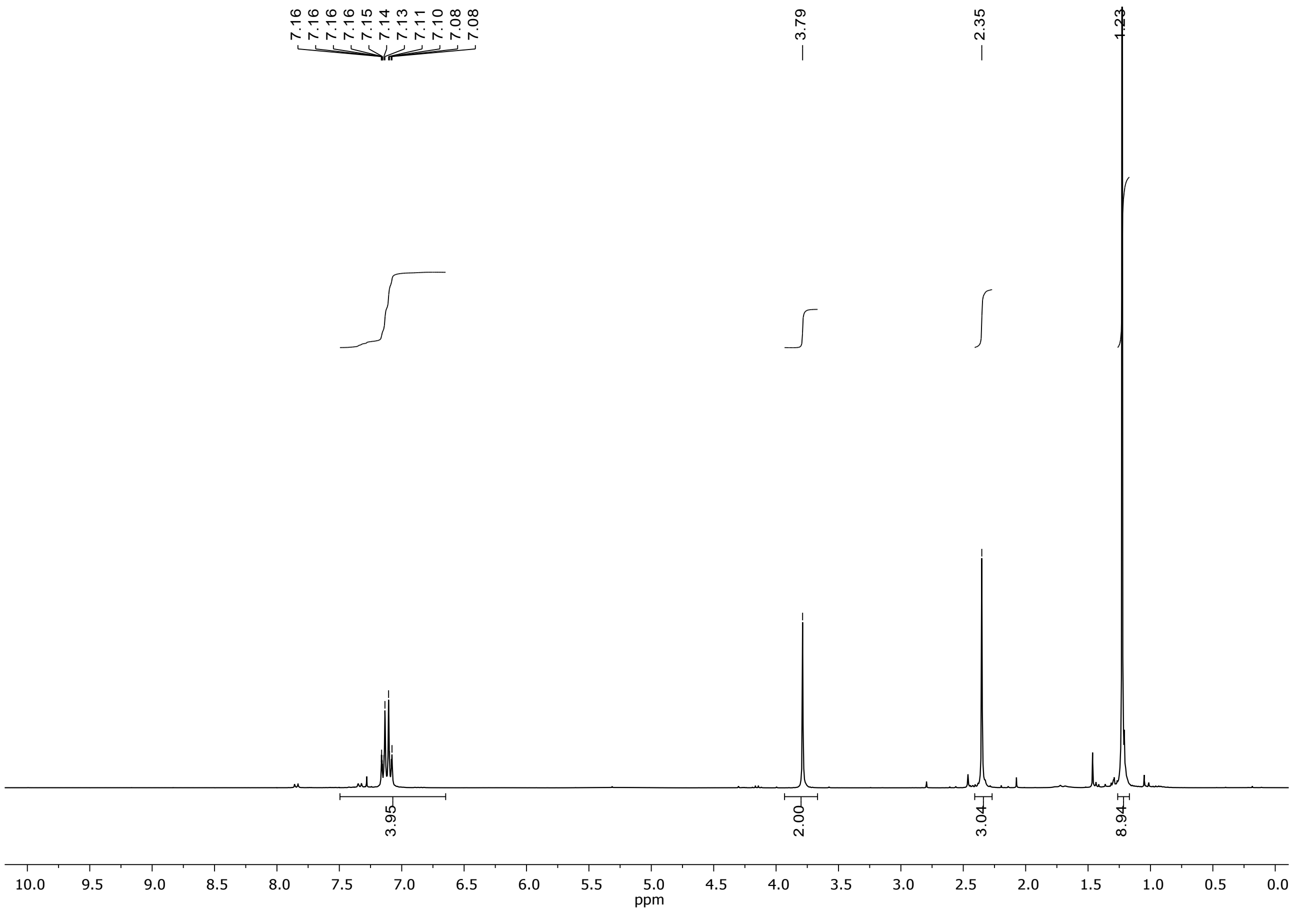

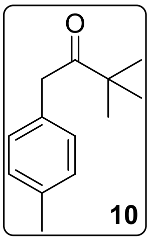

10 ^{13}C NMR (75 MHz, $CDCl_3$)

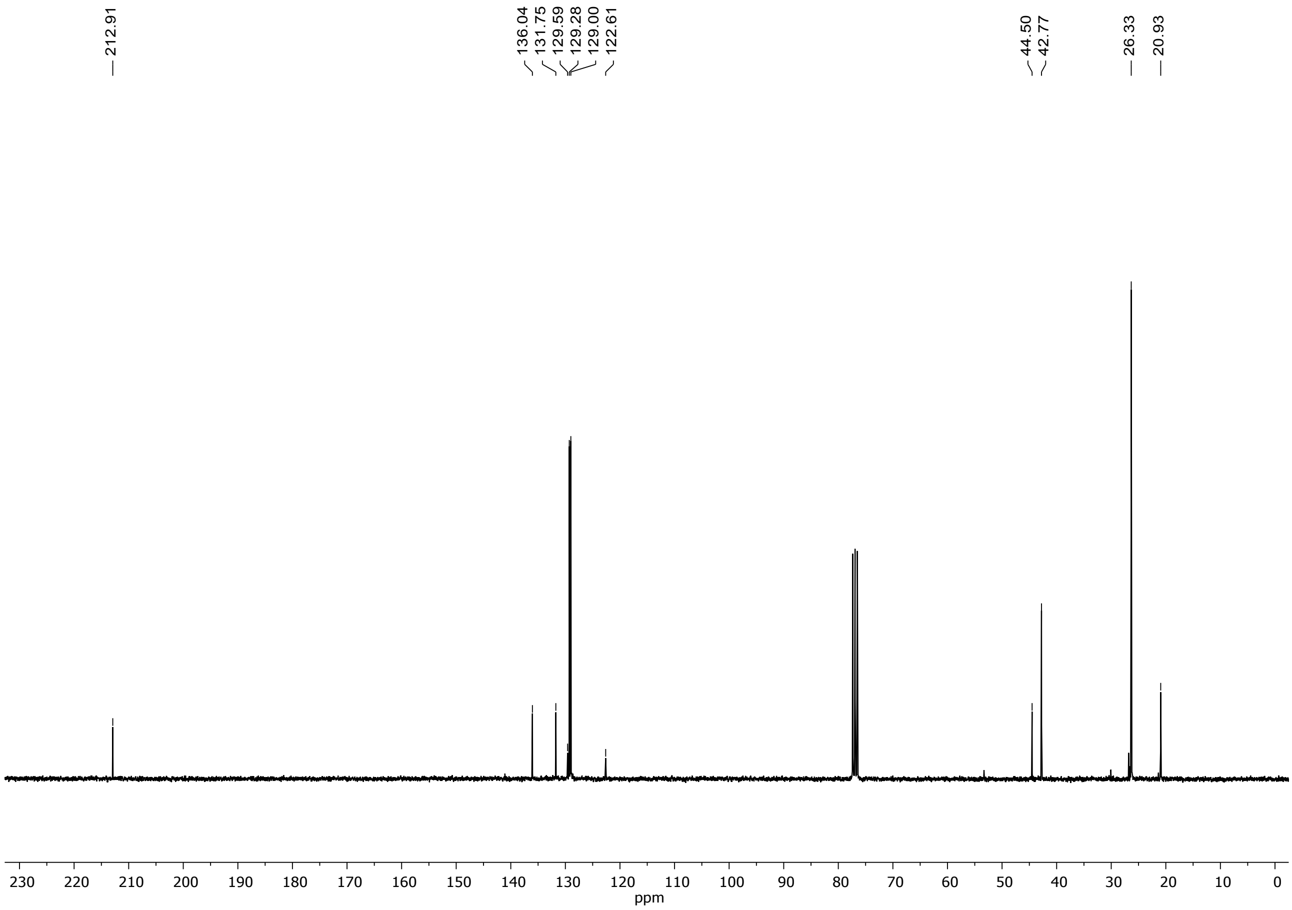

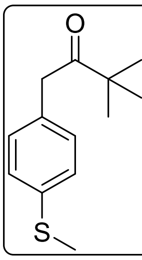

11 ^{1}H NMR (300 MHz, $CDCl_3$)

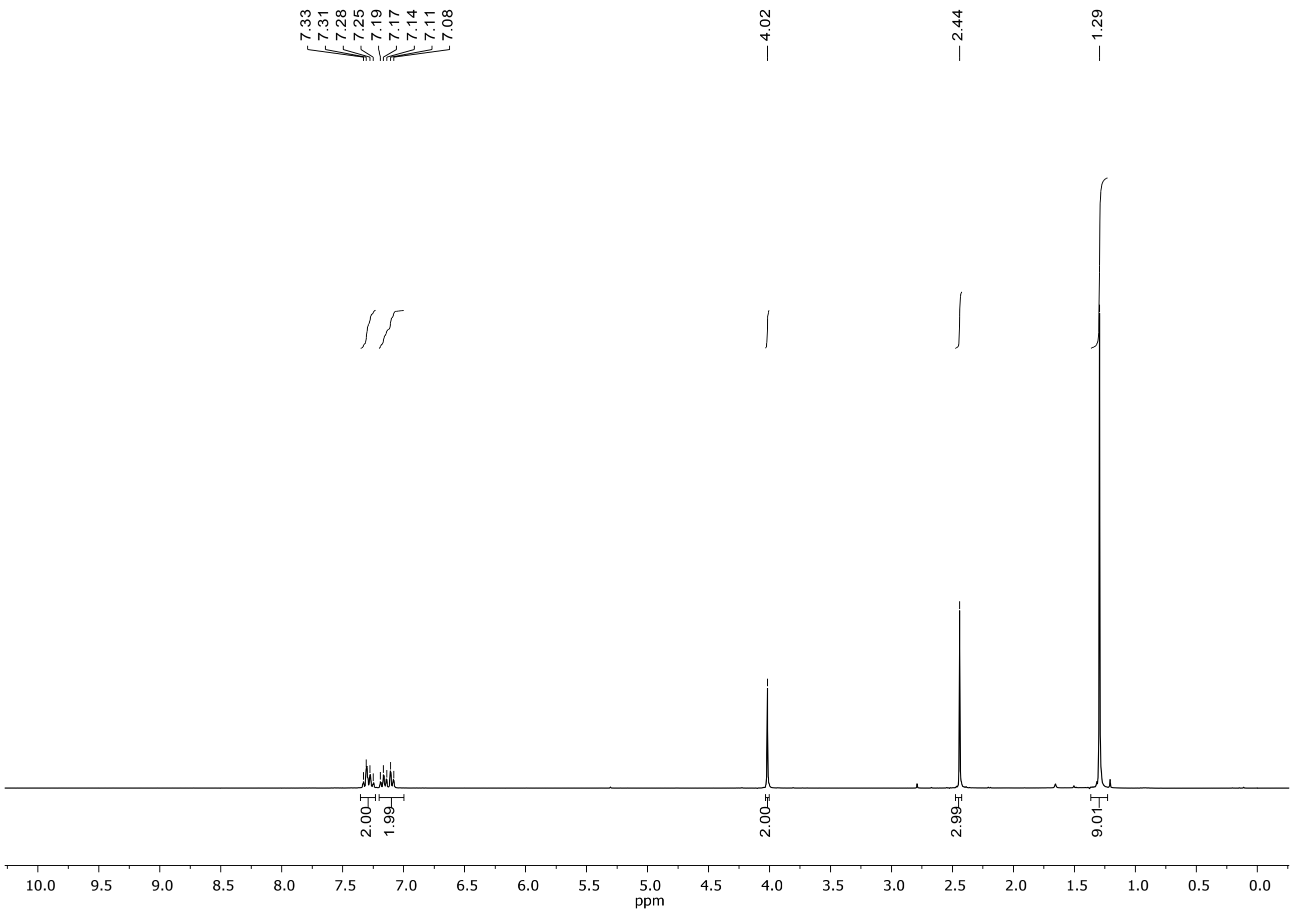

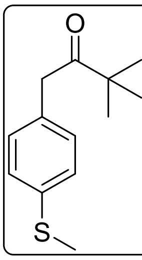

11

^{13}C NMR (75 MHz, $CDCl_3$)

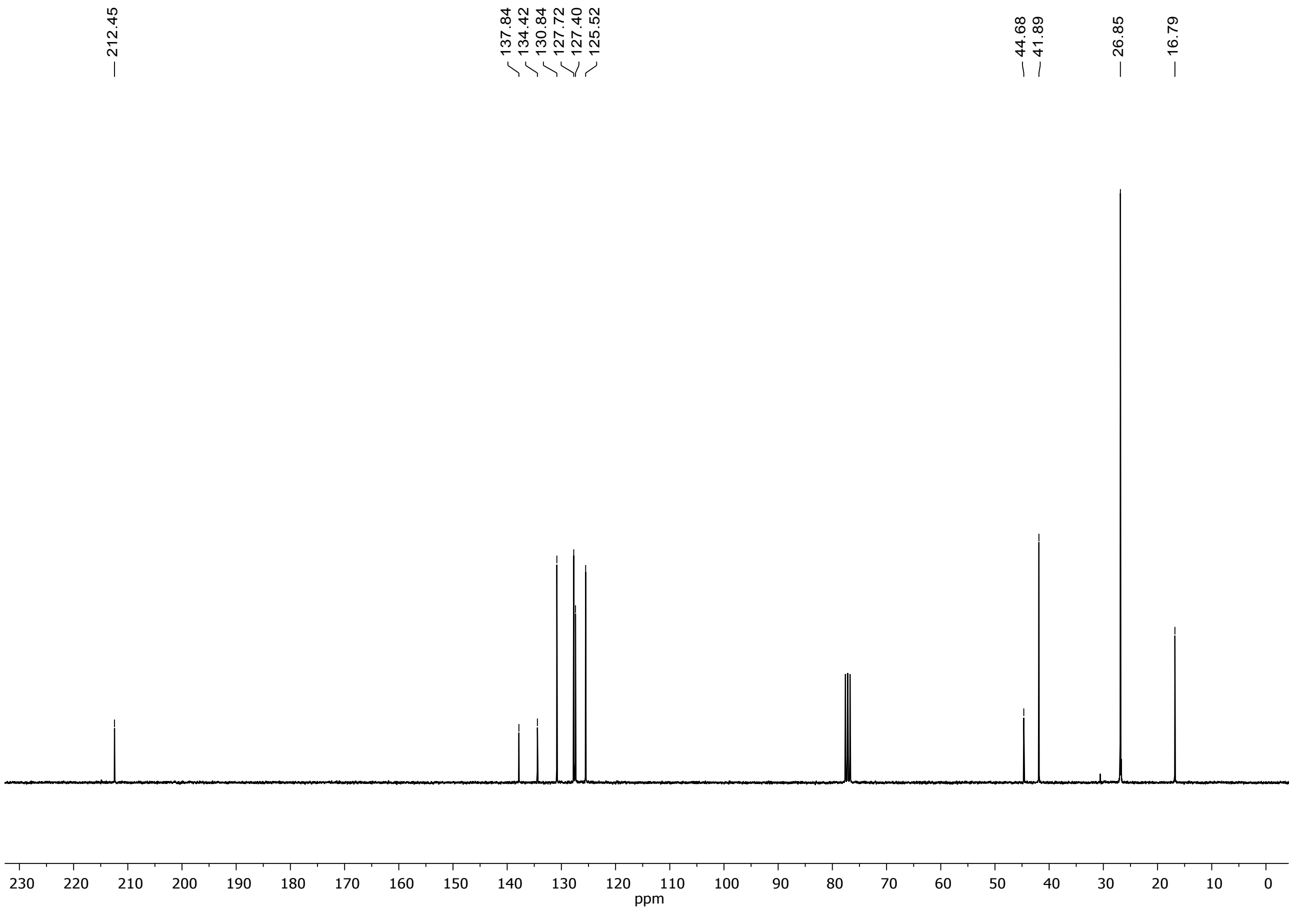

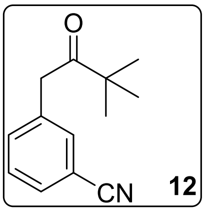

12

^{1}H NMR (300 MHz, $CDCl_3$)

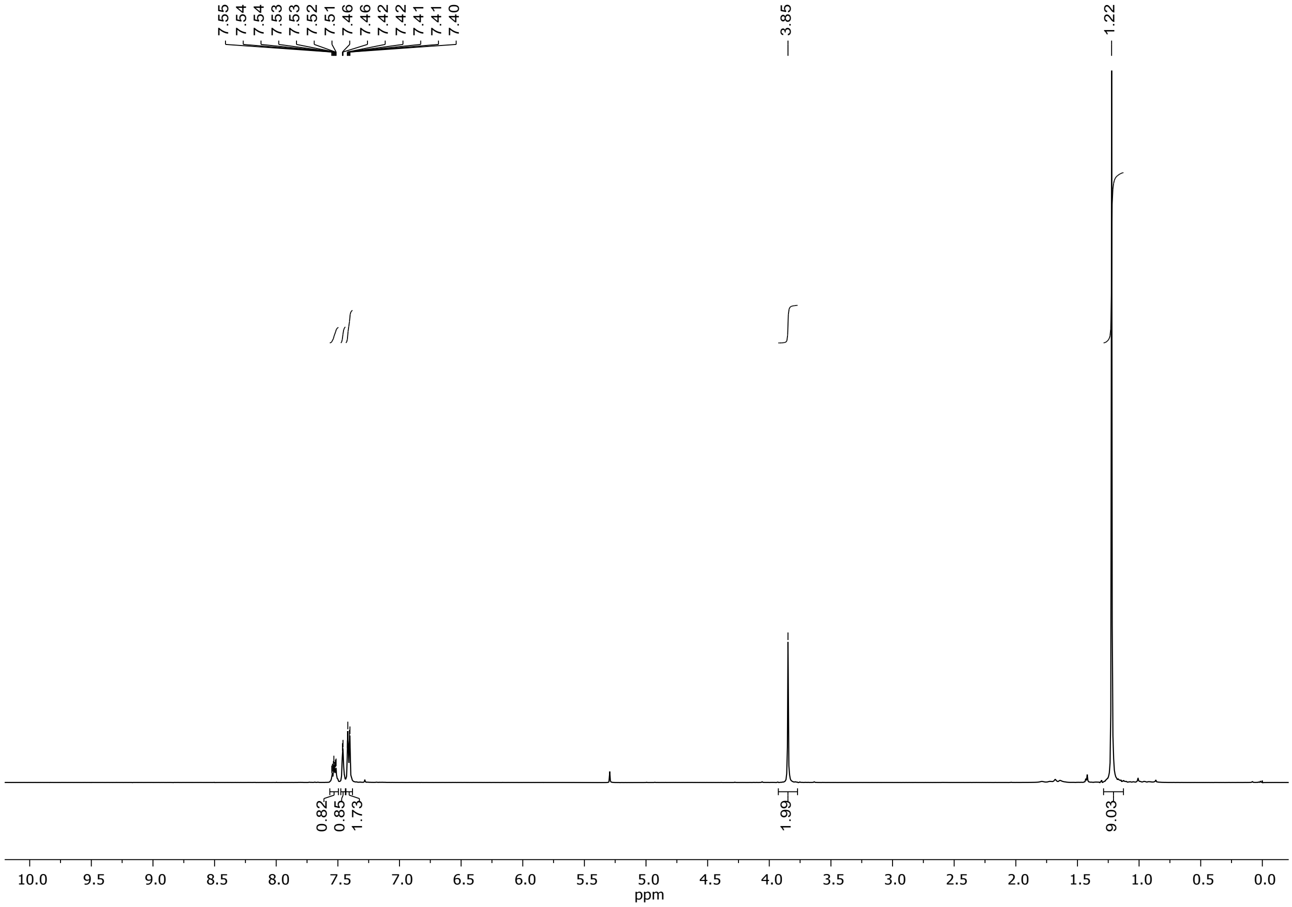

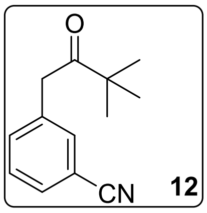

12

^{13}C NMR (75 MHz, $CDCl_3$)

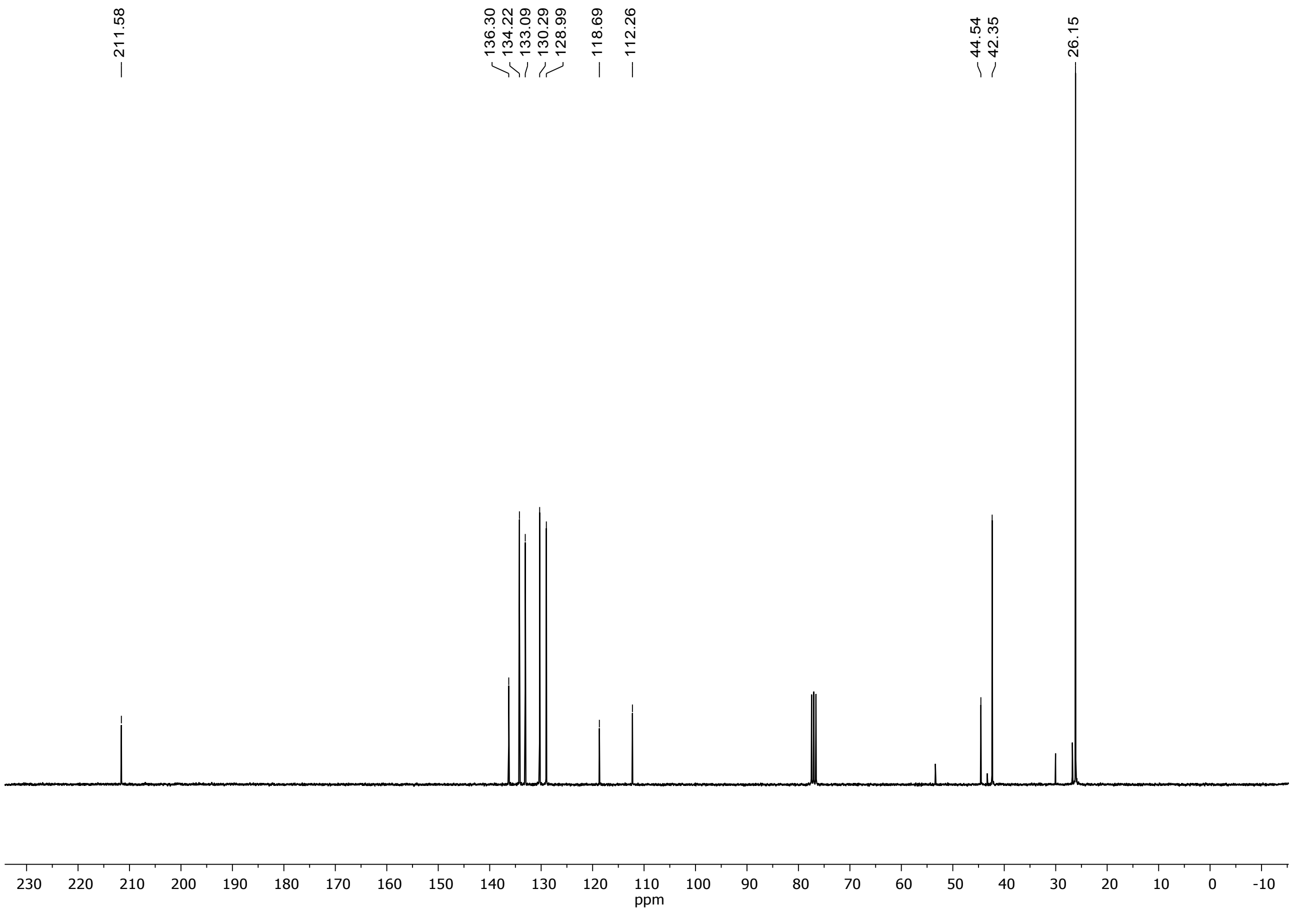

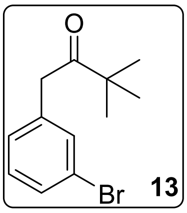

13 ^{1}H NMR (300 MHz, $CDCl_3$)

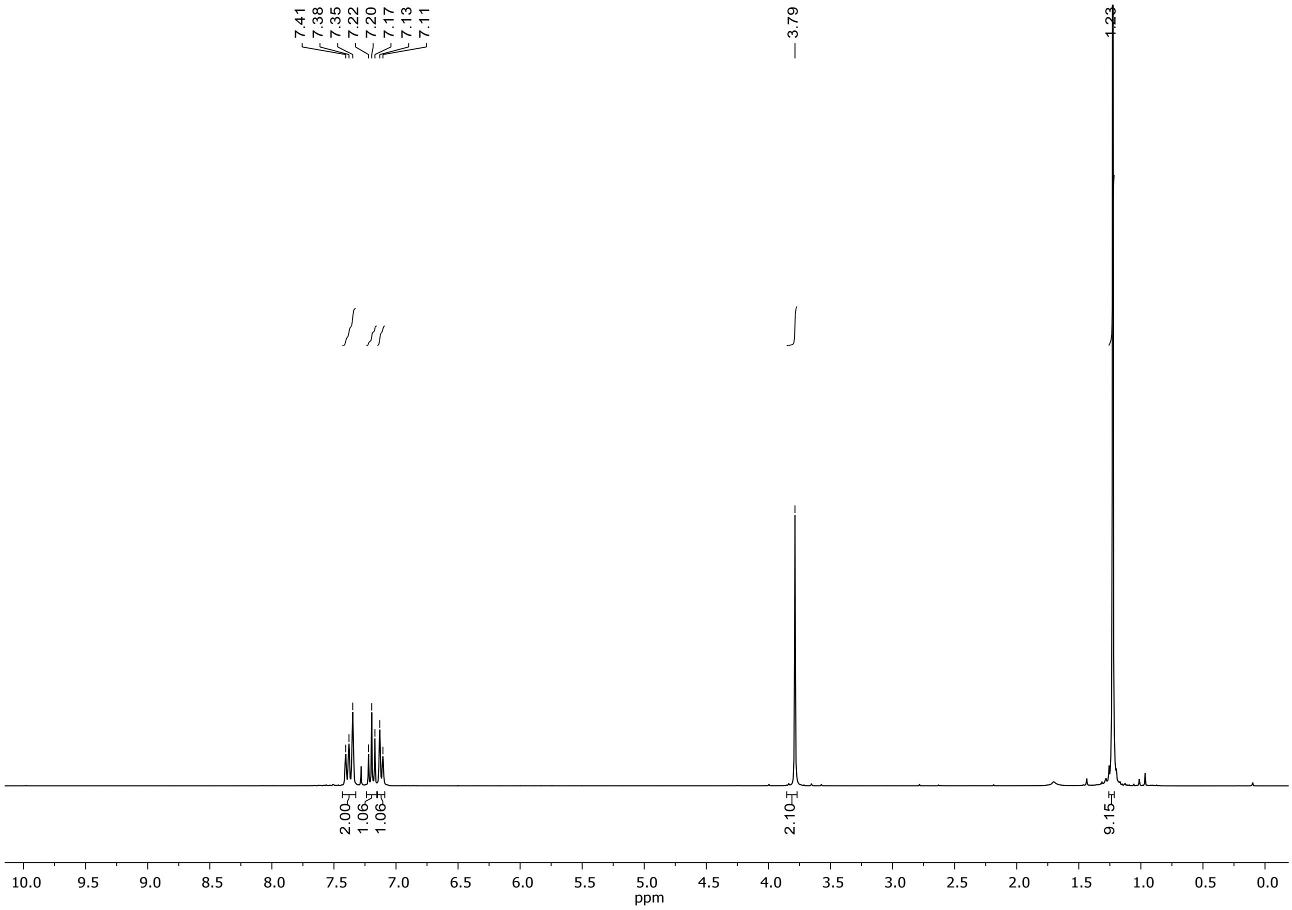

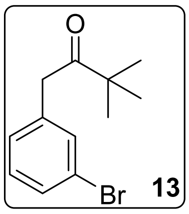

13

^{13}C NMR (75 MHz, $CDCl_3$)

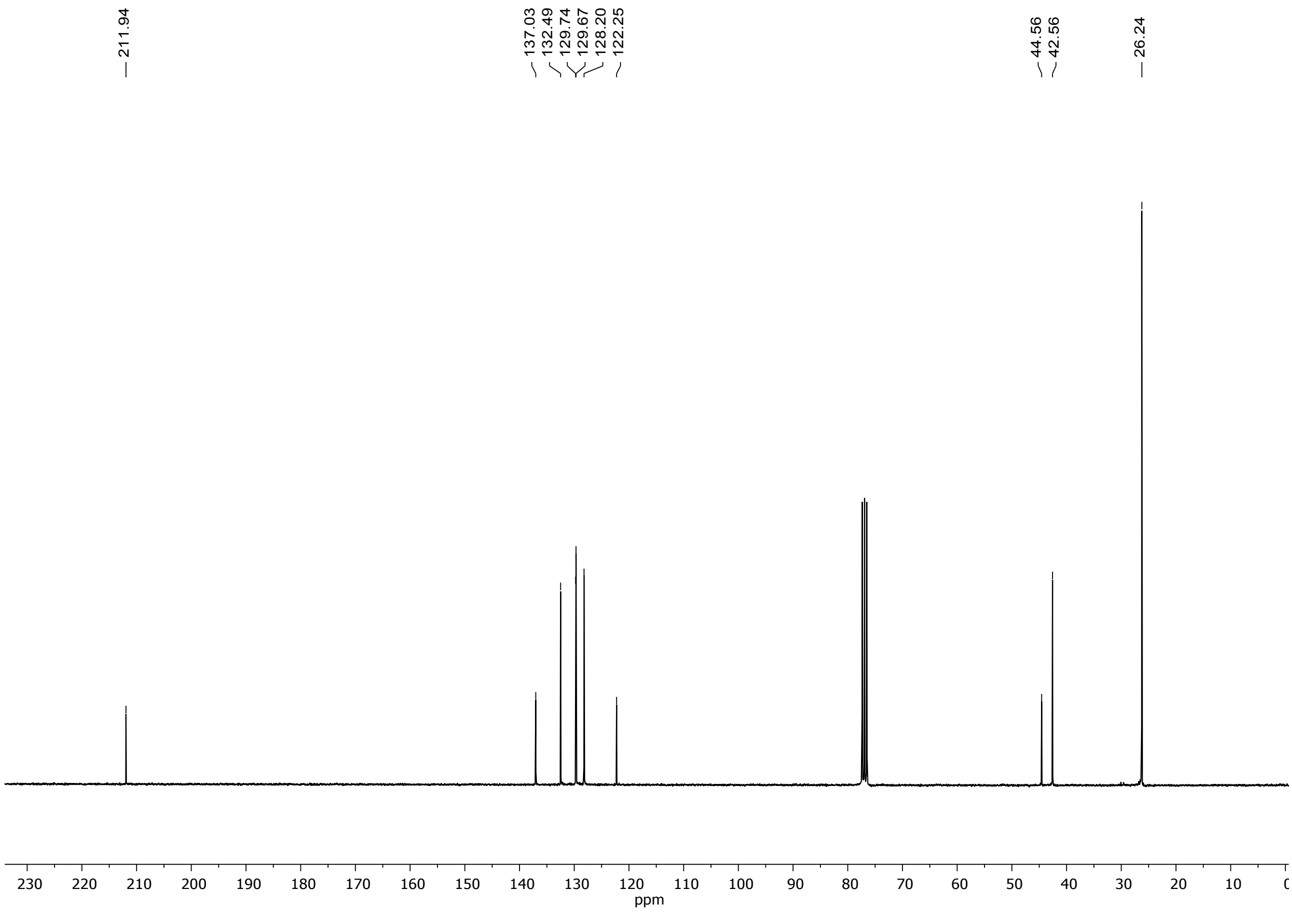

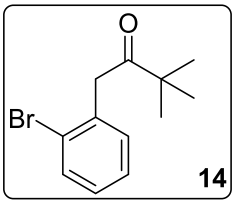

14 ^{1}H NMR (300 MHz, $CDCl_3$)

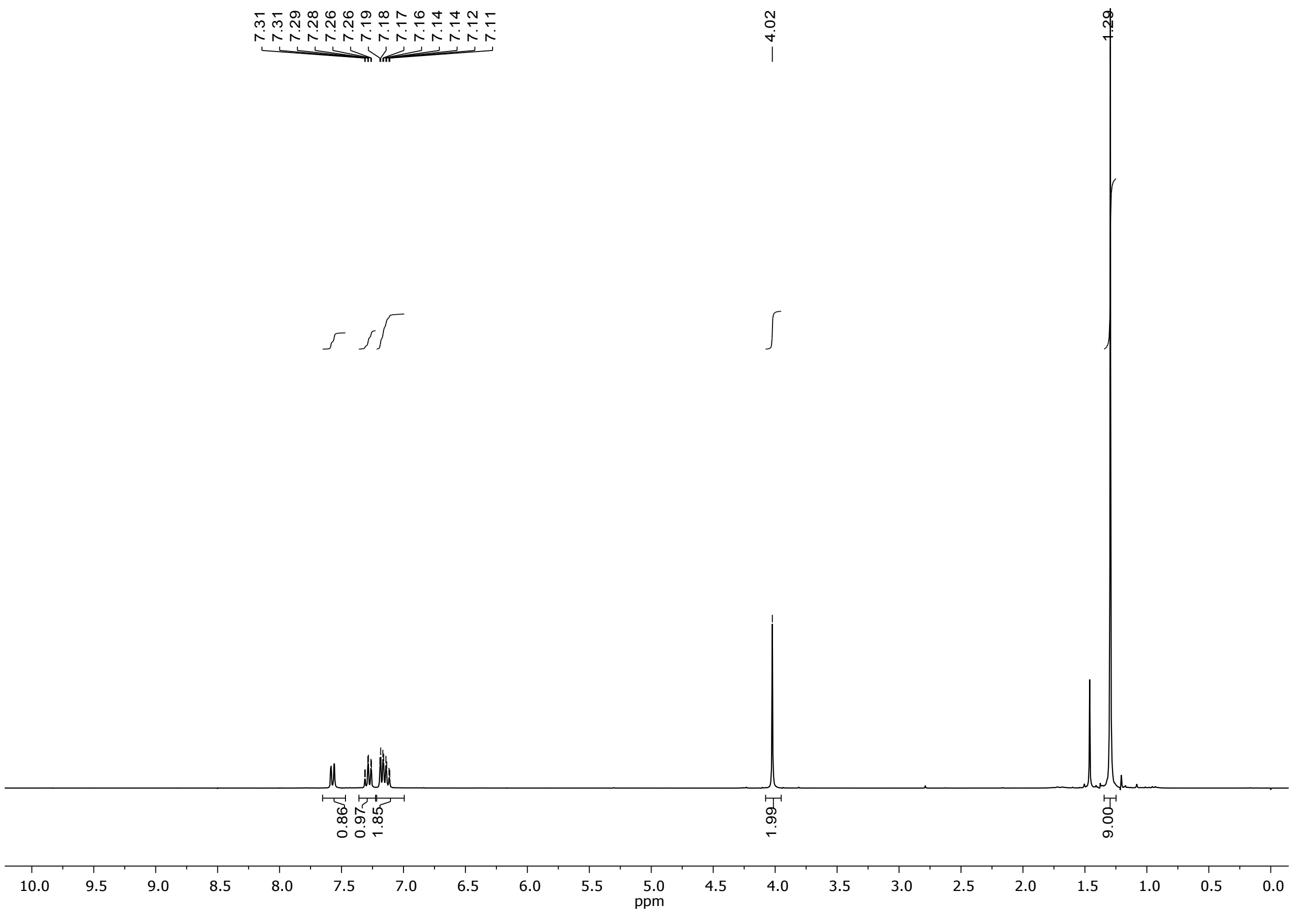

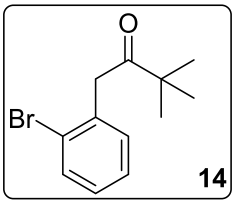

^{13}C NMR (75 MHz, $CDCl_3$)

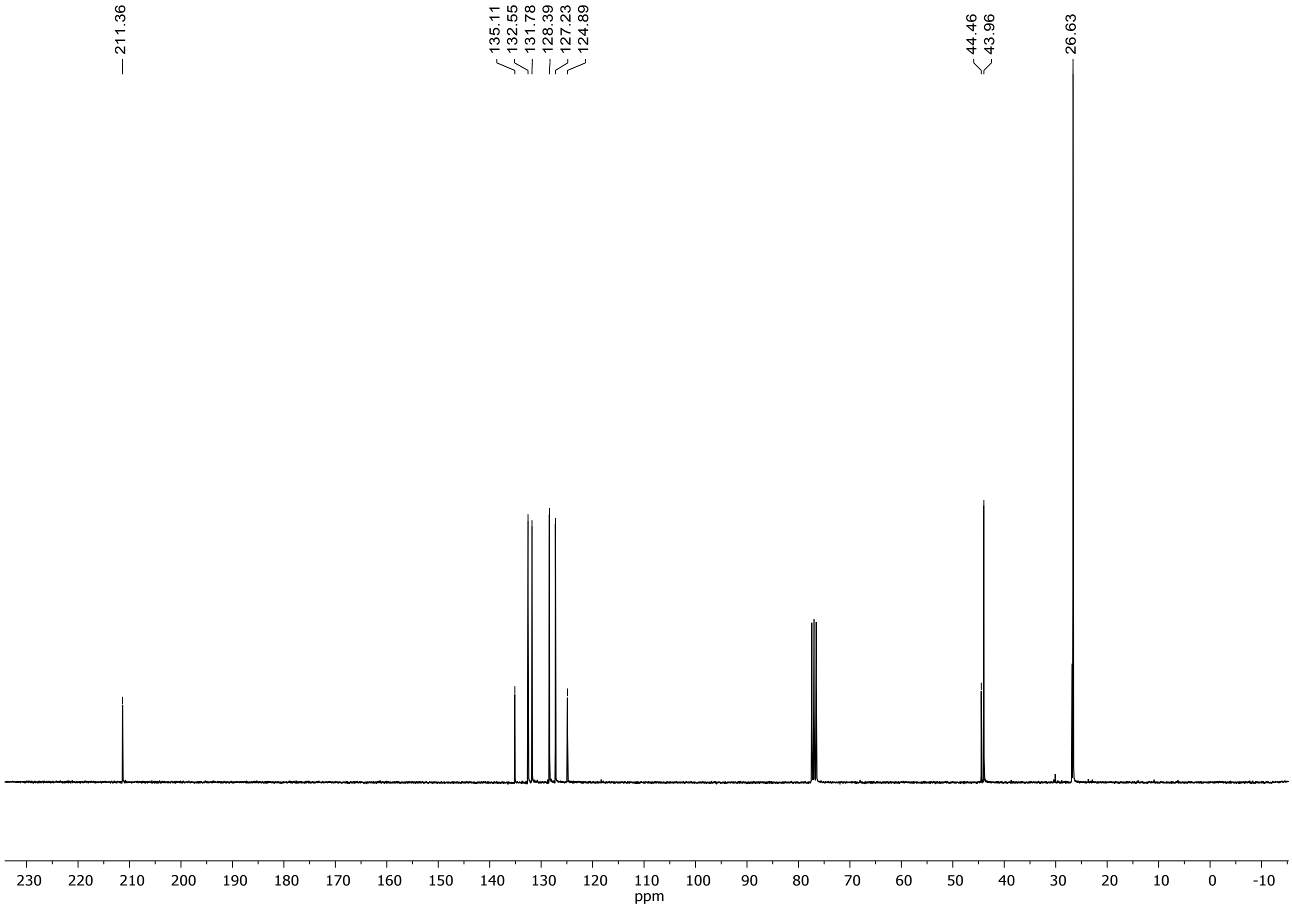

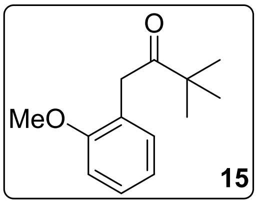

^{1}H NMR (300 MHz, $CDCl_3$)

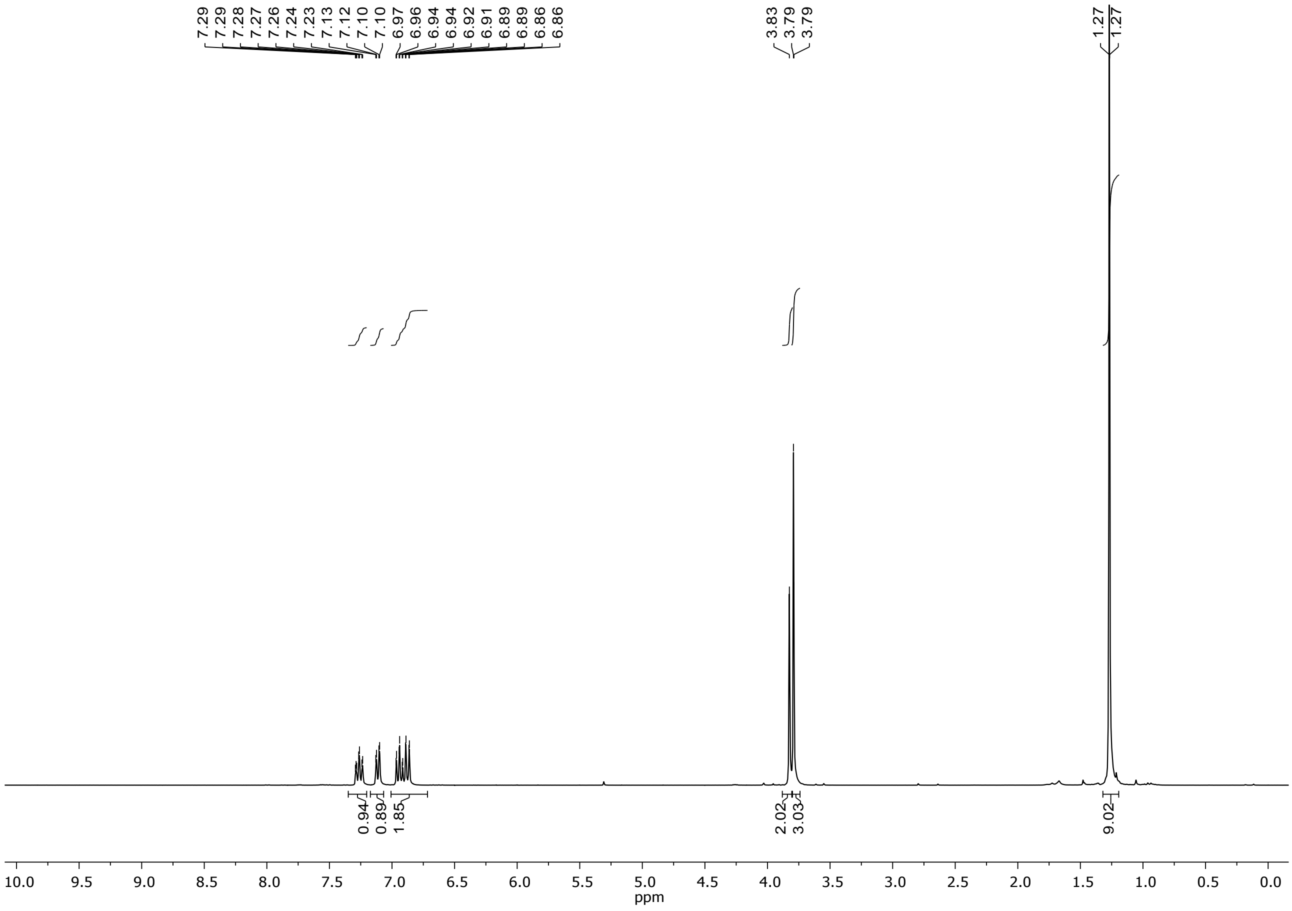

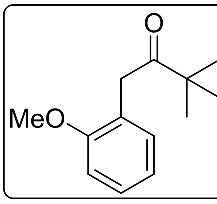

15

^{13}C NMR (75 MHz, $CDCl_3$)

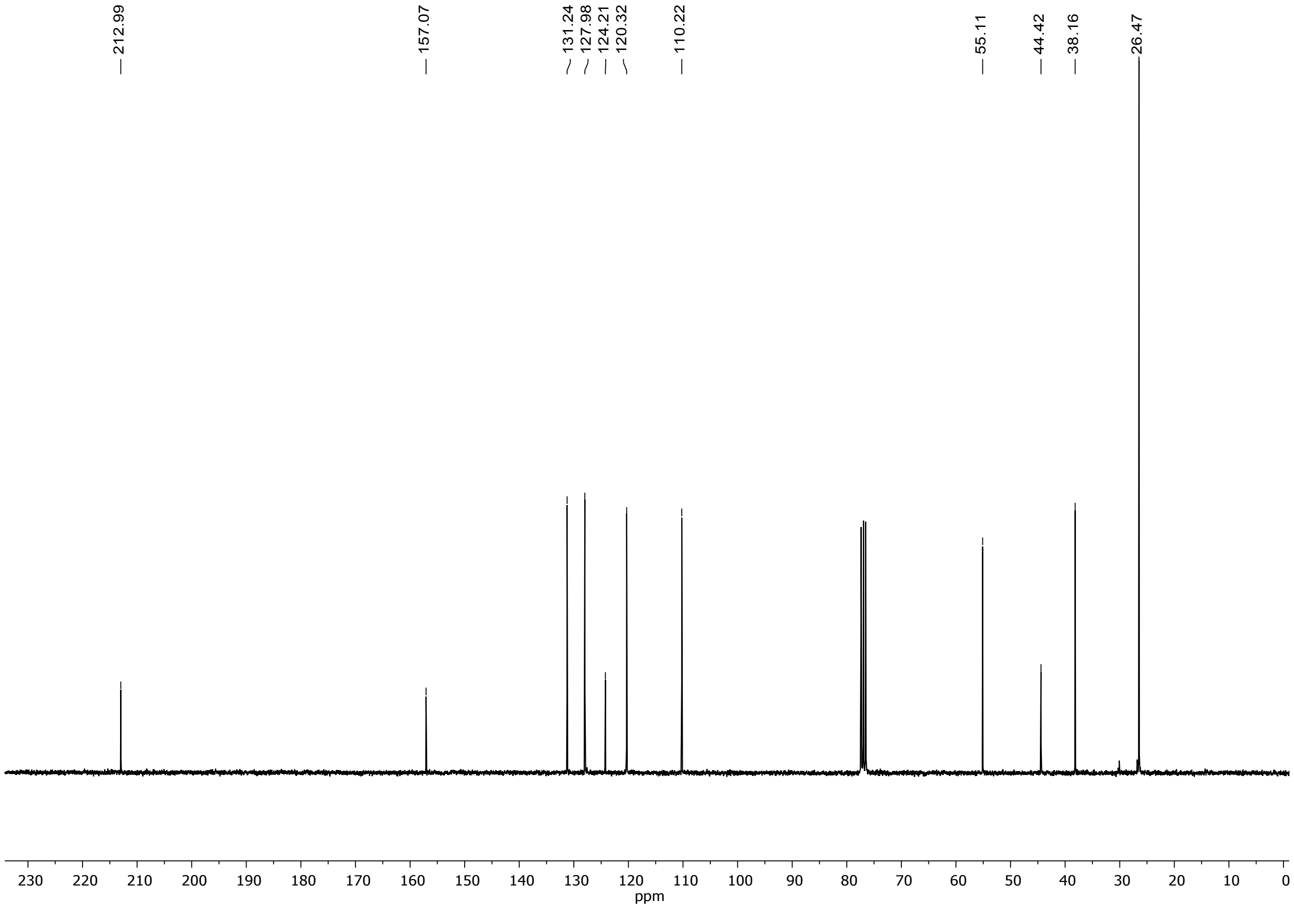

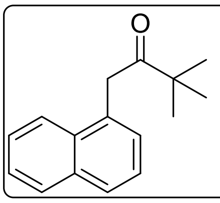

16 ^{1}H NMR (300 MHz, $CDCl_3$)

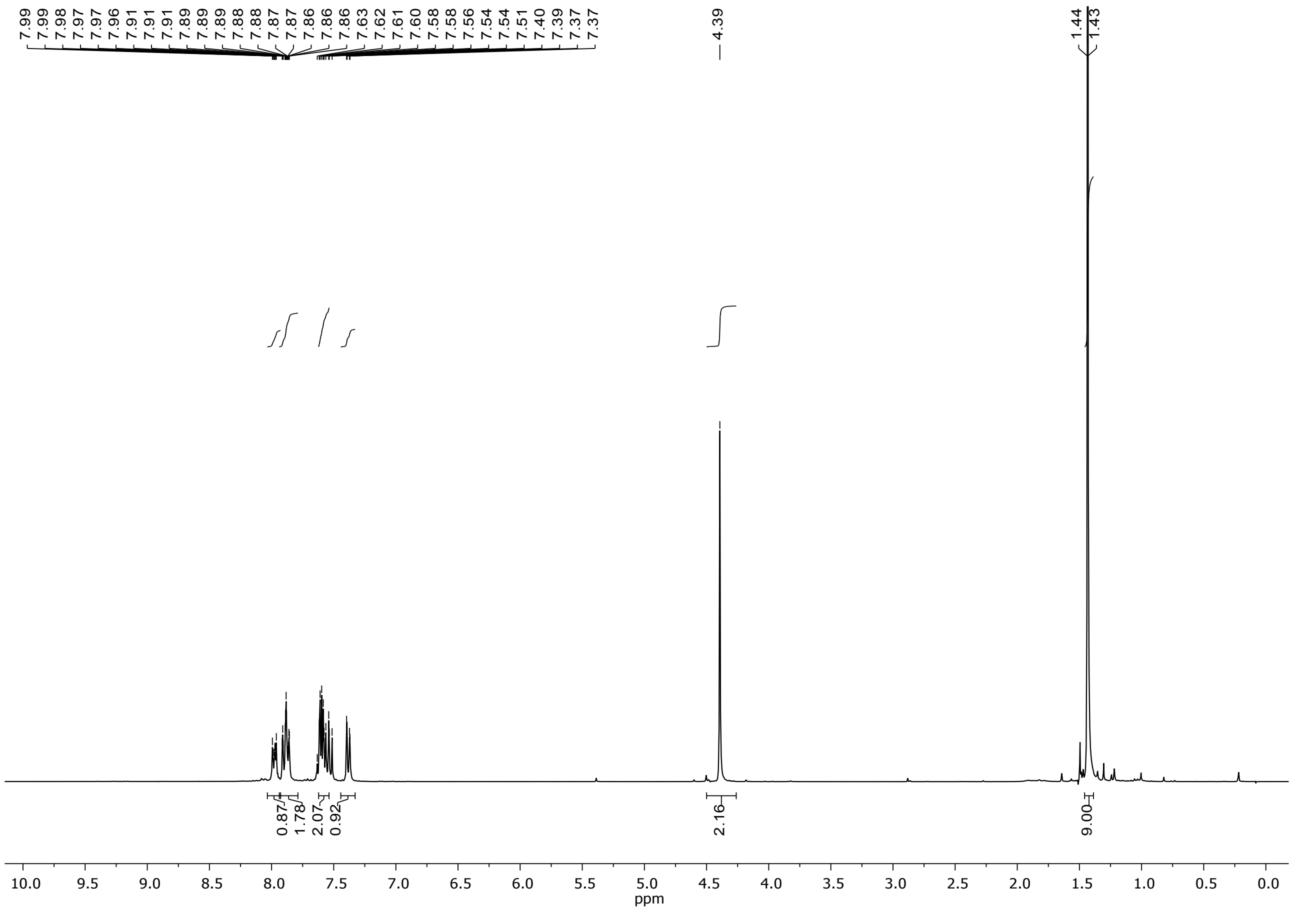

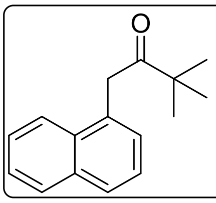
16

^{13}C NMR (75 MHz, $CDCl_3$)

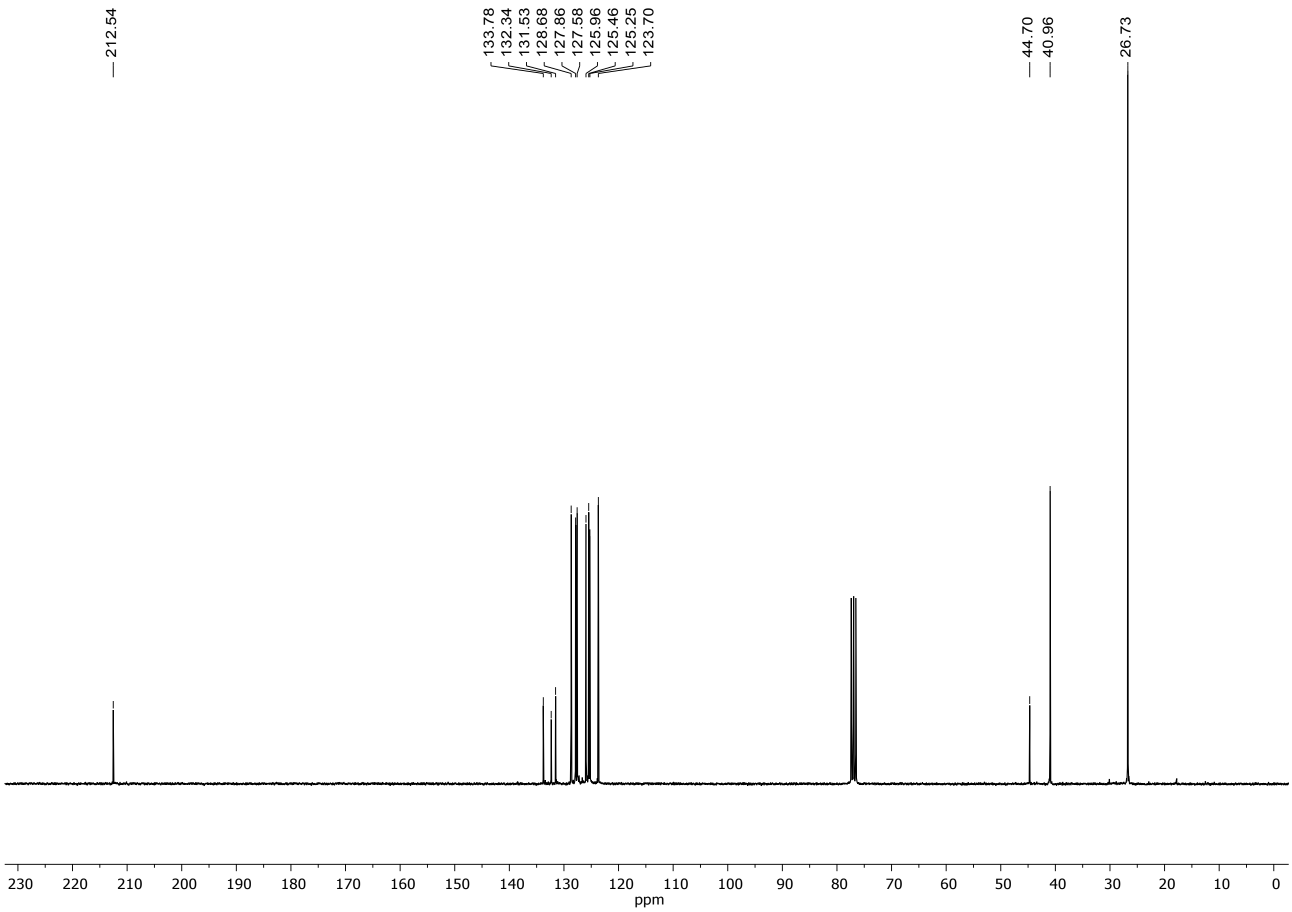

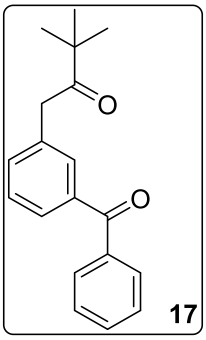

17

^{1}H NMR (300 MHz, $CDCl_3$)

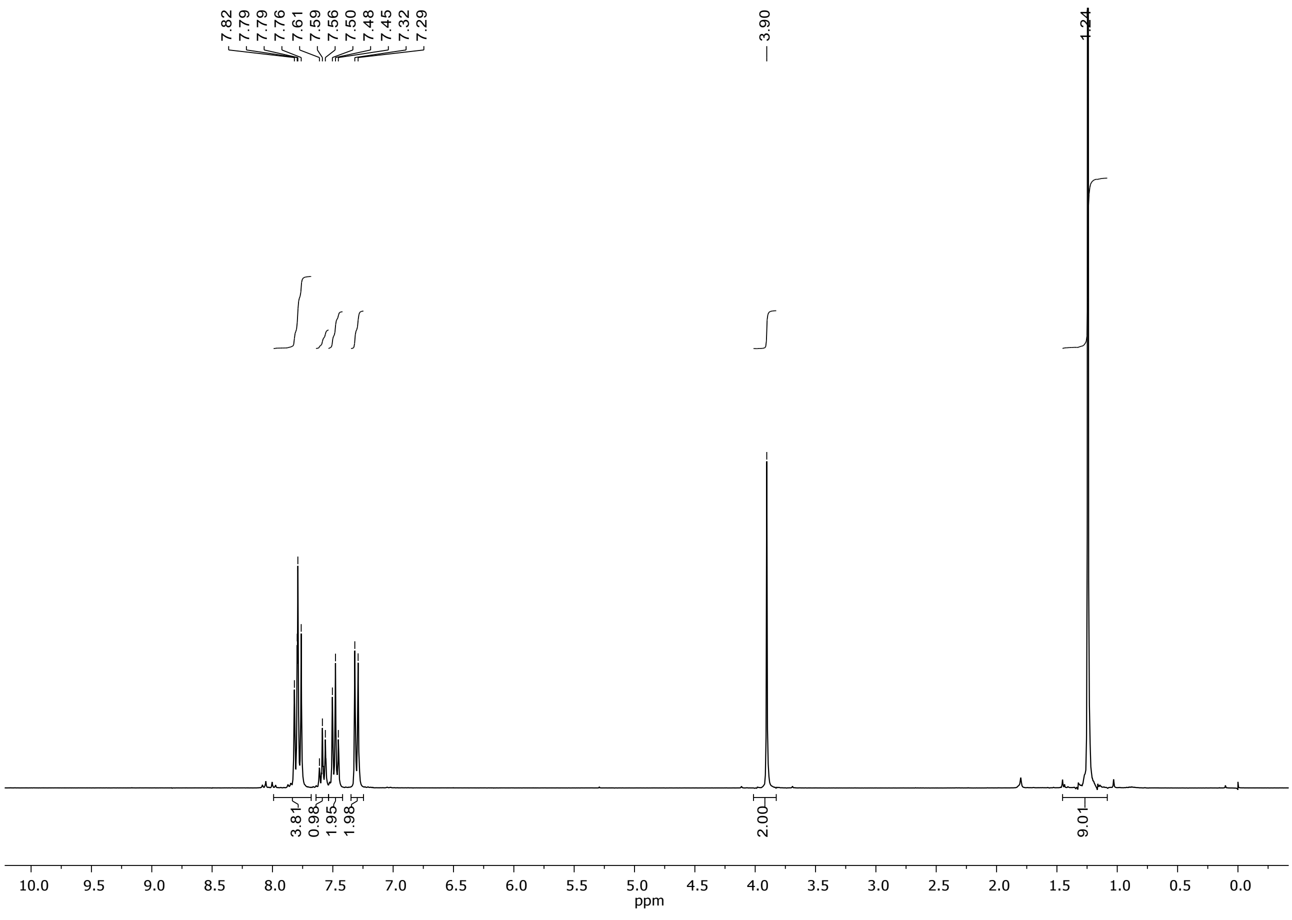

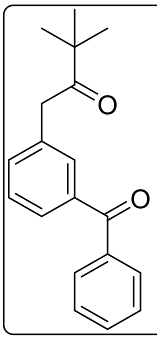

17

^{13}C NMR (75 MHz, $CDCl_3$)

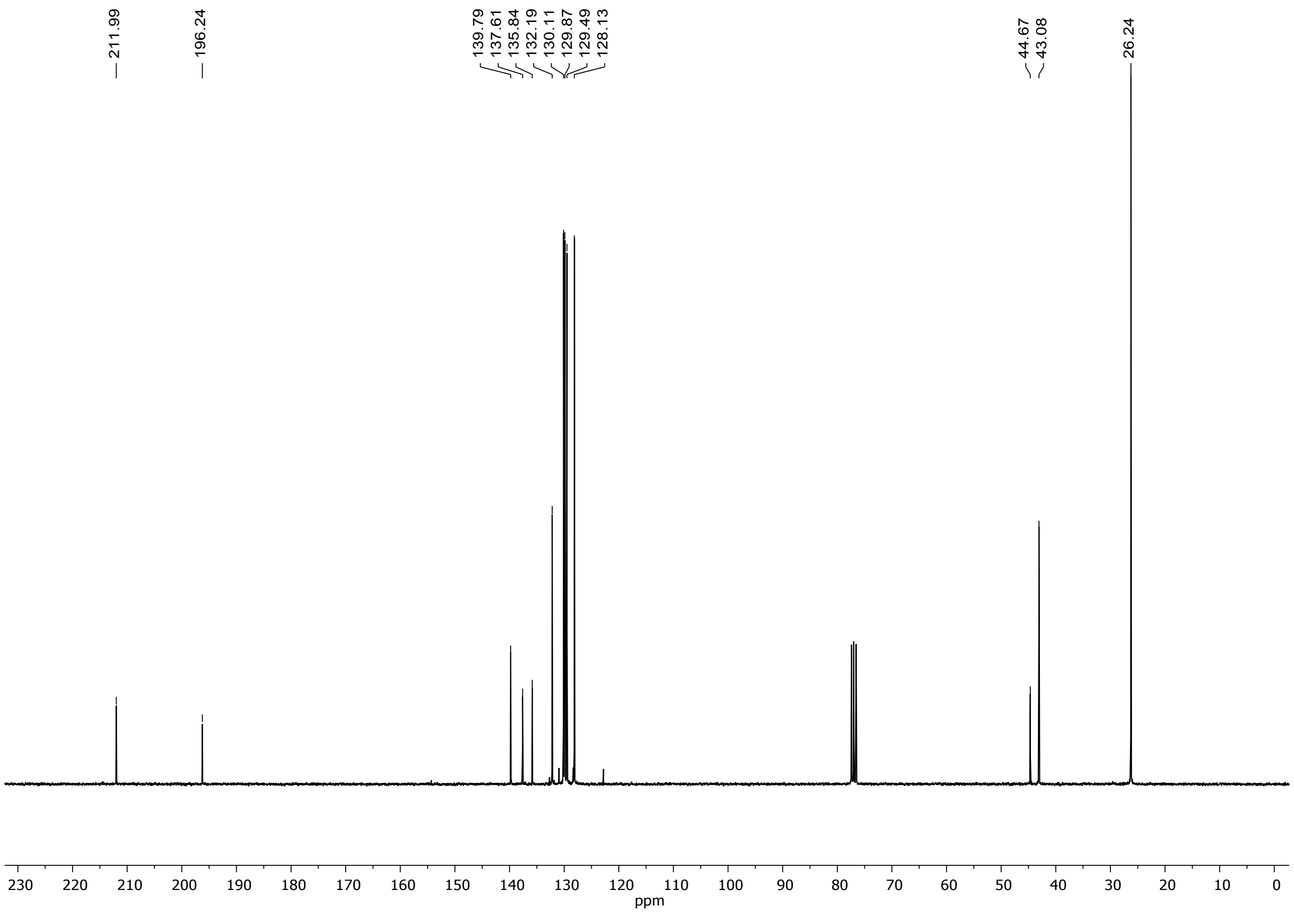

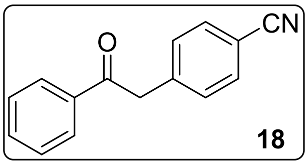

^{1}H NMR (300 MHz, $CDCl_3$)

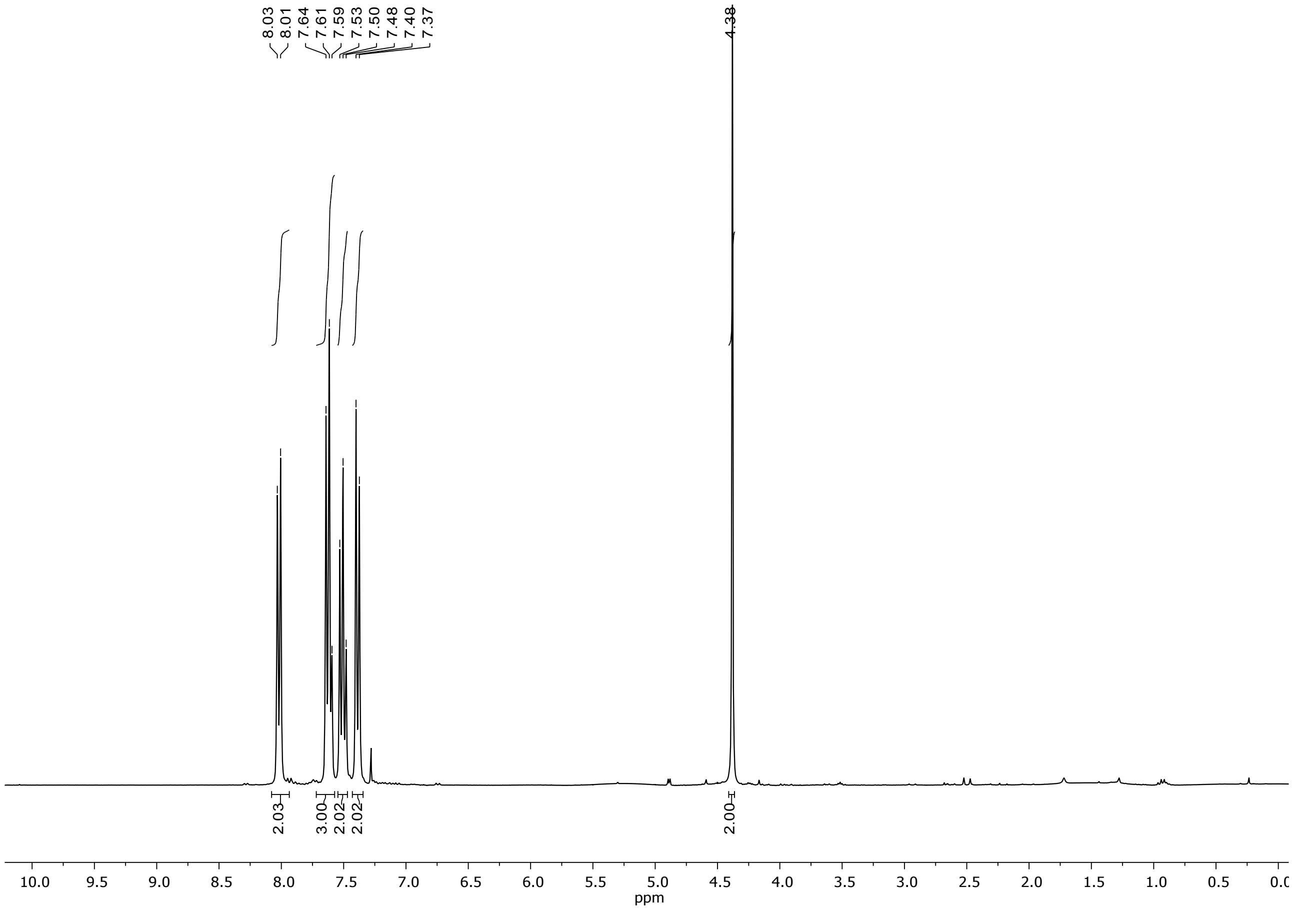

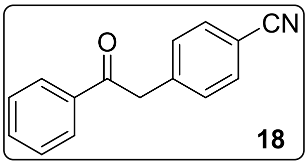

18

^{13}C NMR (75 MHz, $CDCl_3$)

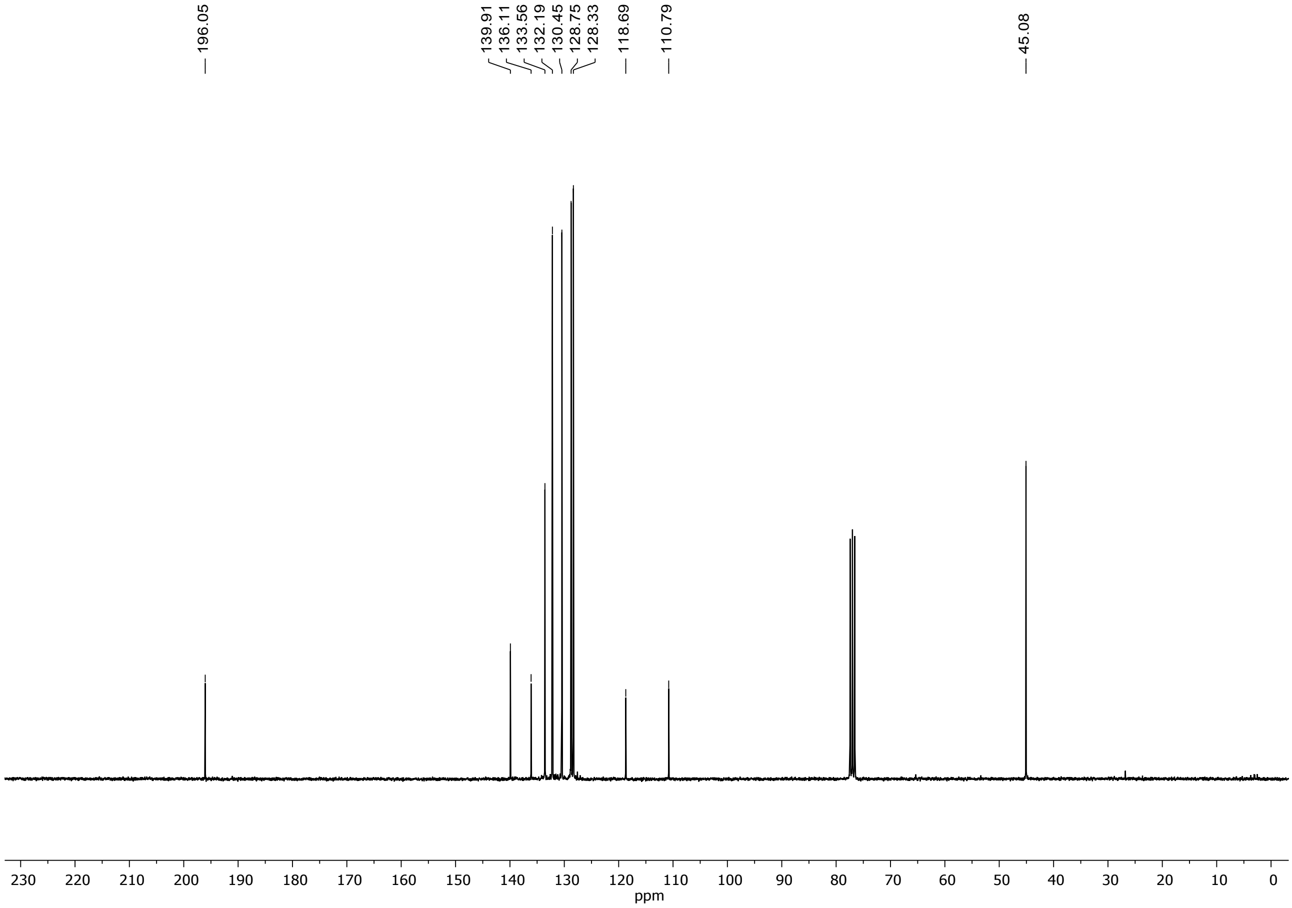

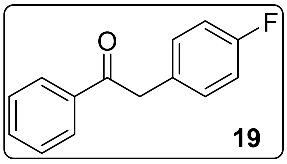

19

^{1}H NMR (300 MHz, $CDCl_3$)

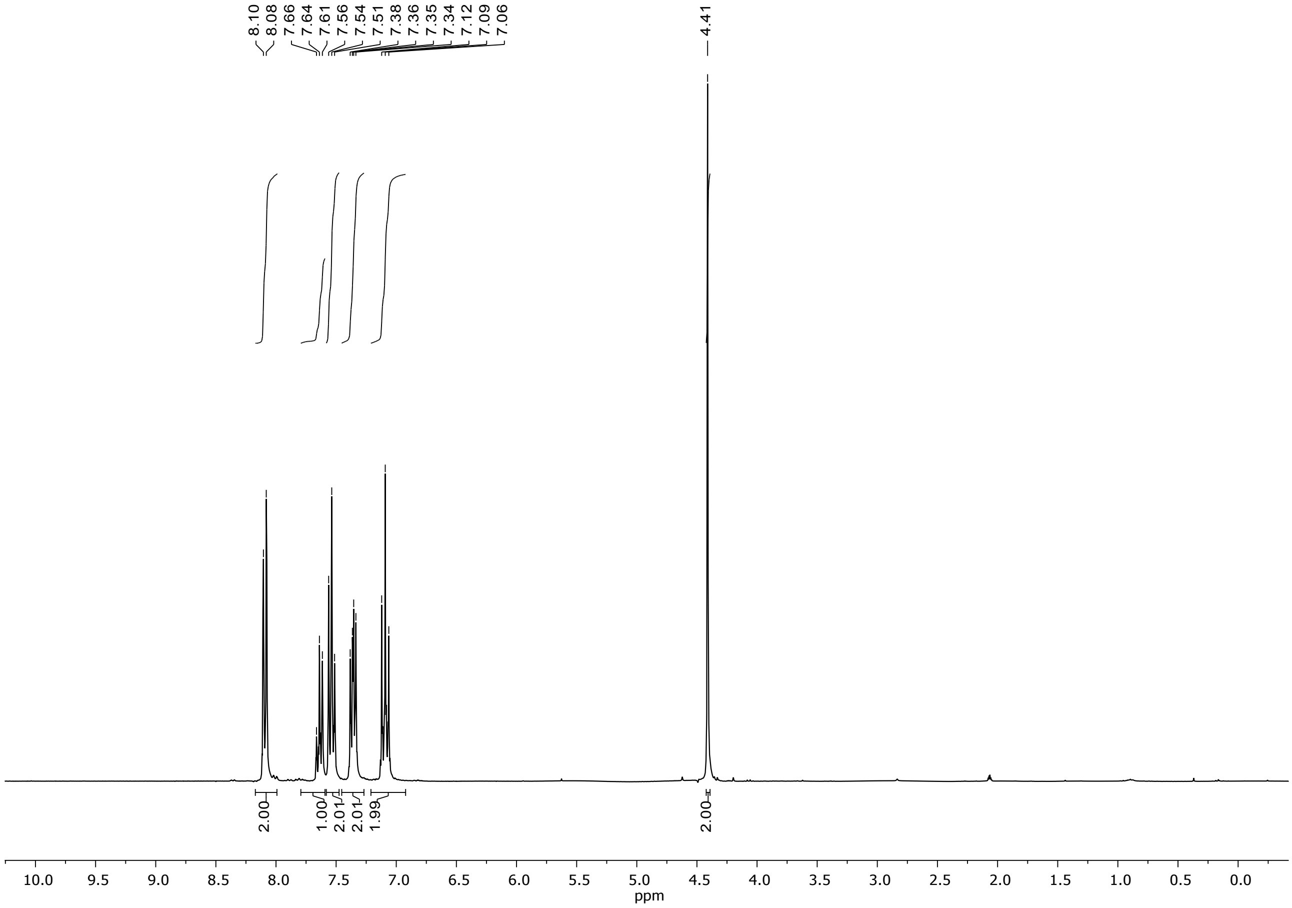

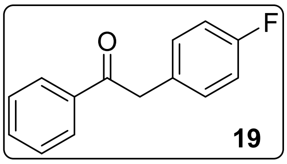

^{13}C NMR (75 MHz, $CDCl_3$)

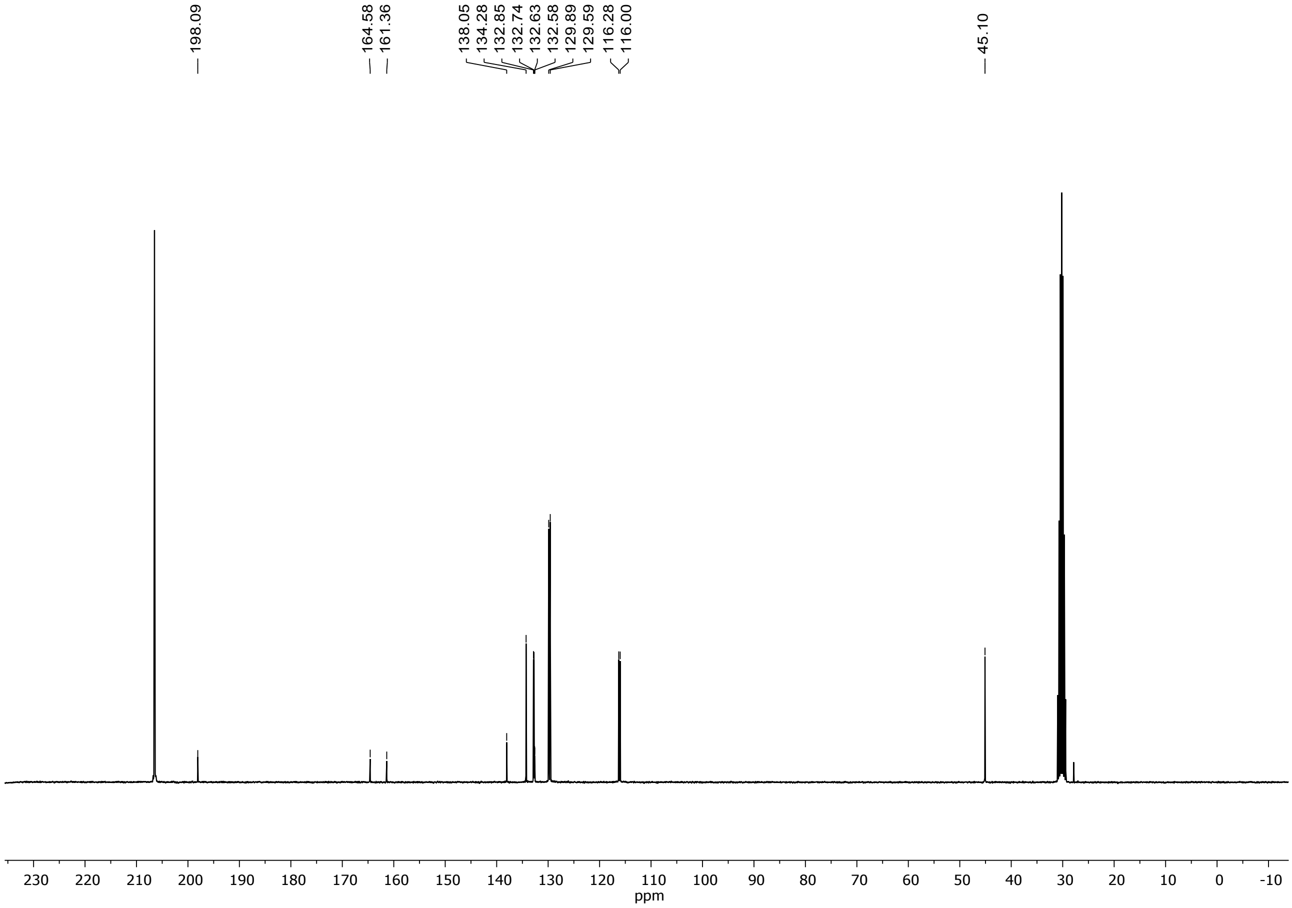

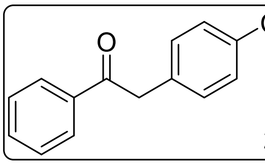

20 ^1H NMR (300 MHz, $CDCl_3$)

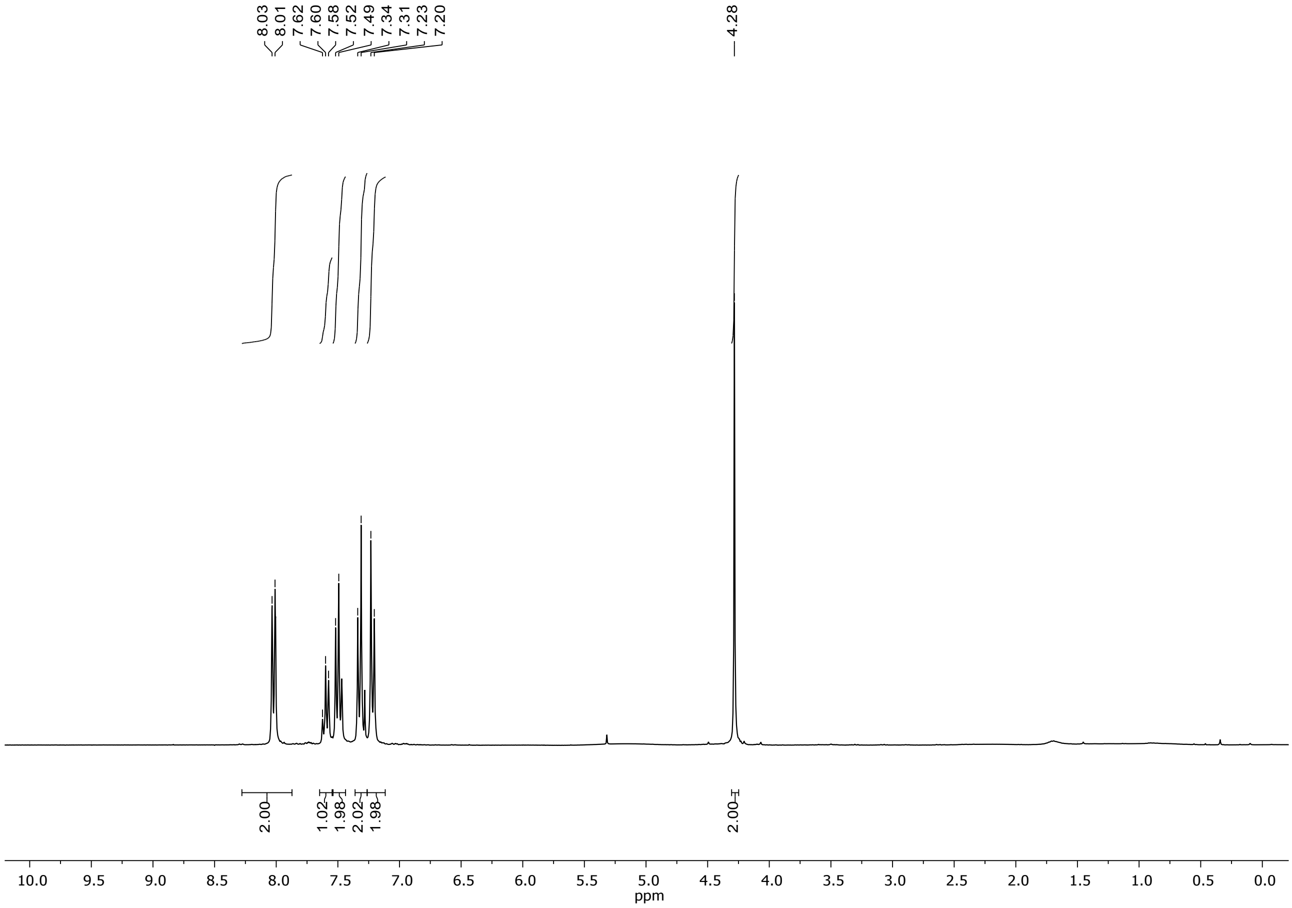

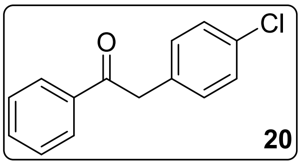

20

^{13}C NMR (75 MHz, $CDCl_3$)

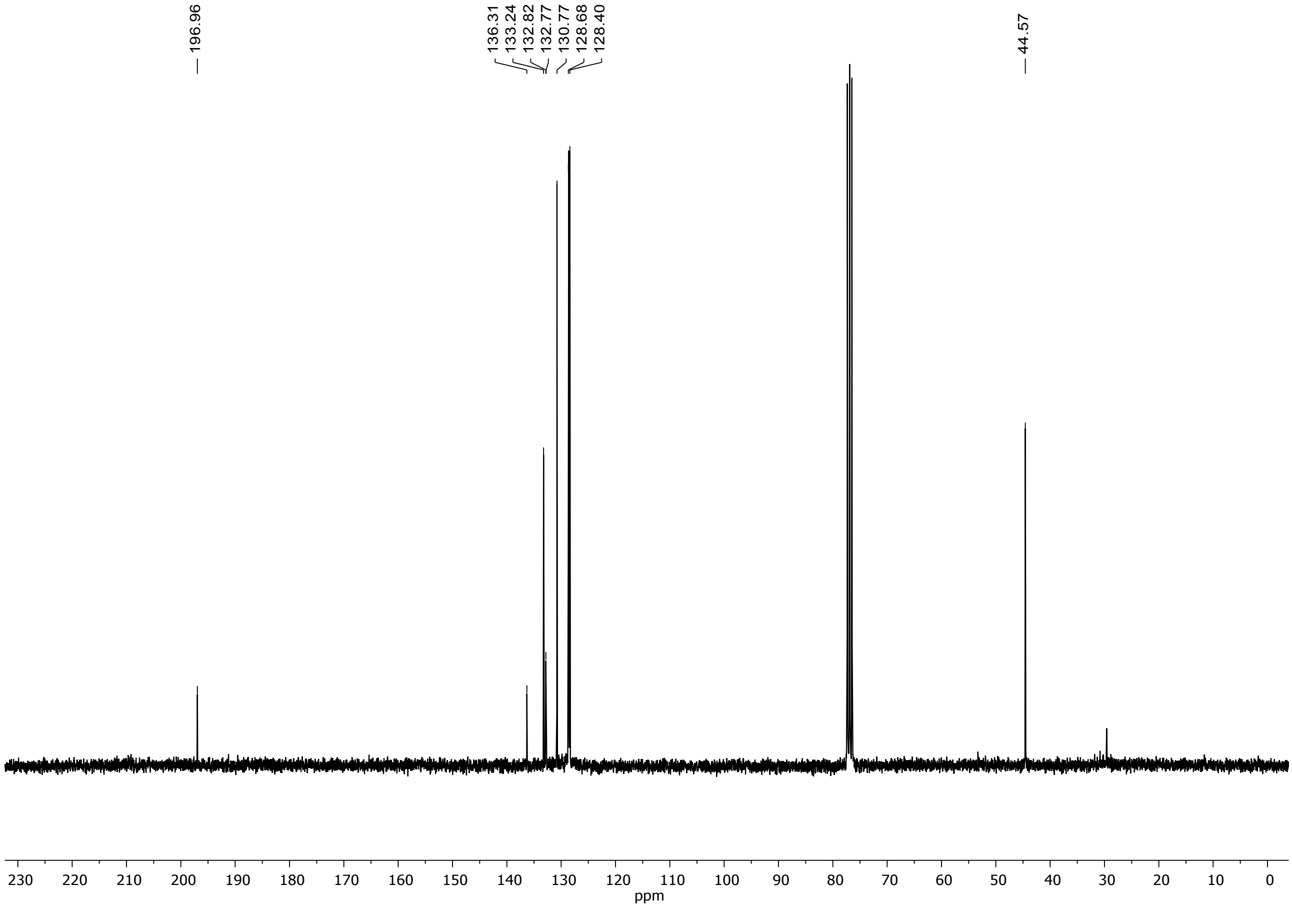

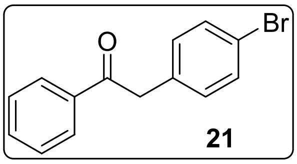

21

^{1}H NMR (300 MHz, $CDCl_3$)

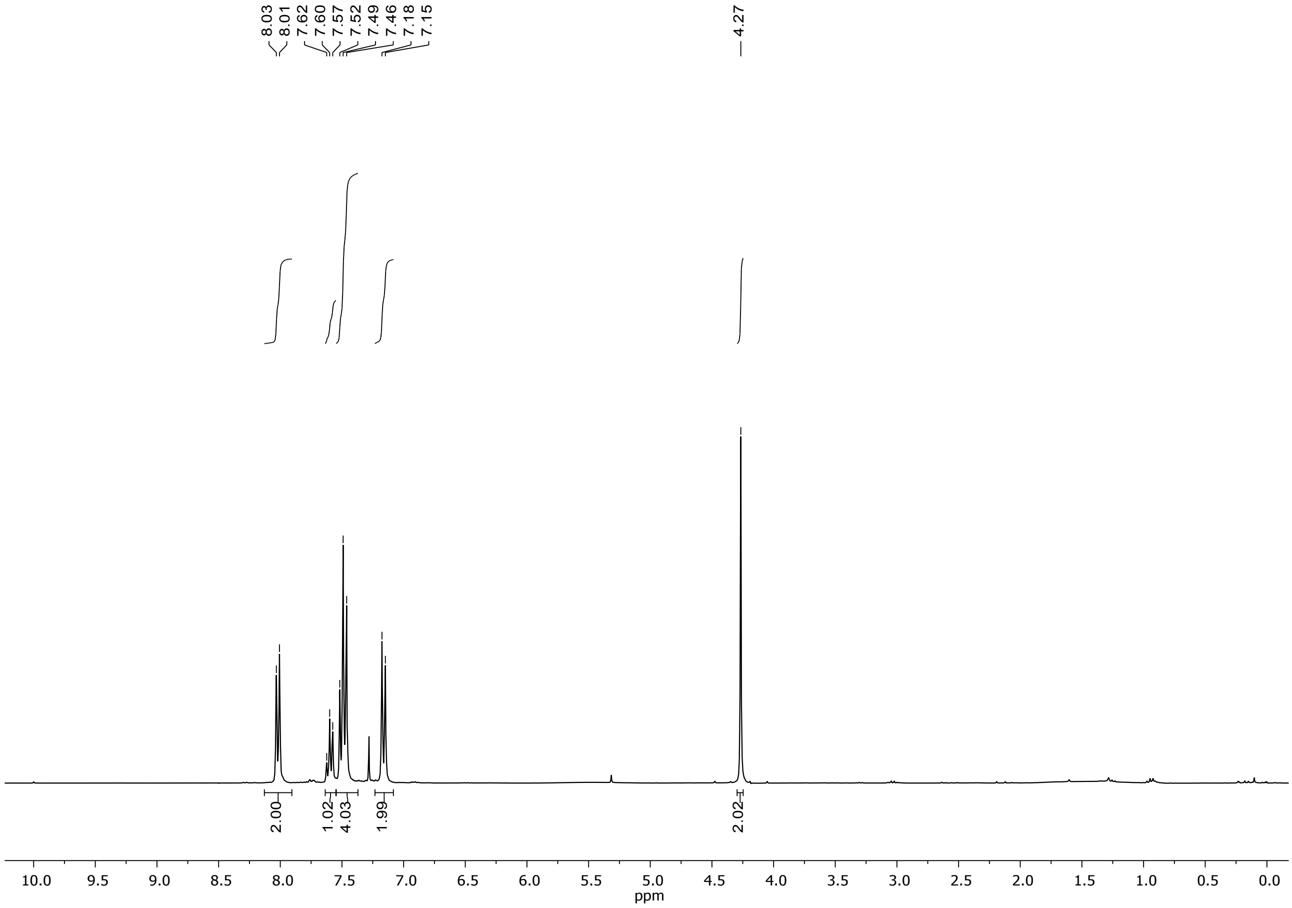

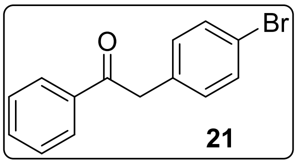

21

^{13}C NMR (75 MHz, $CDCl_3$)

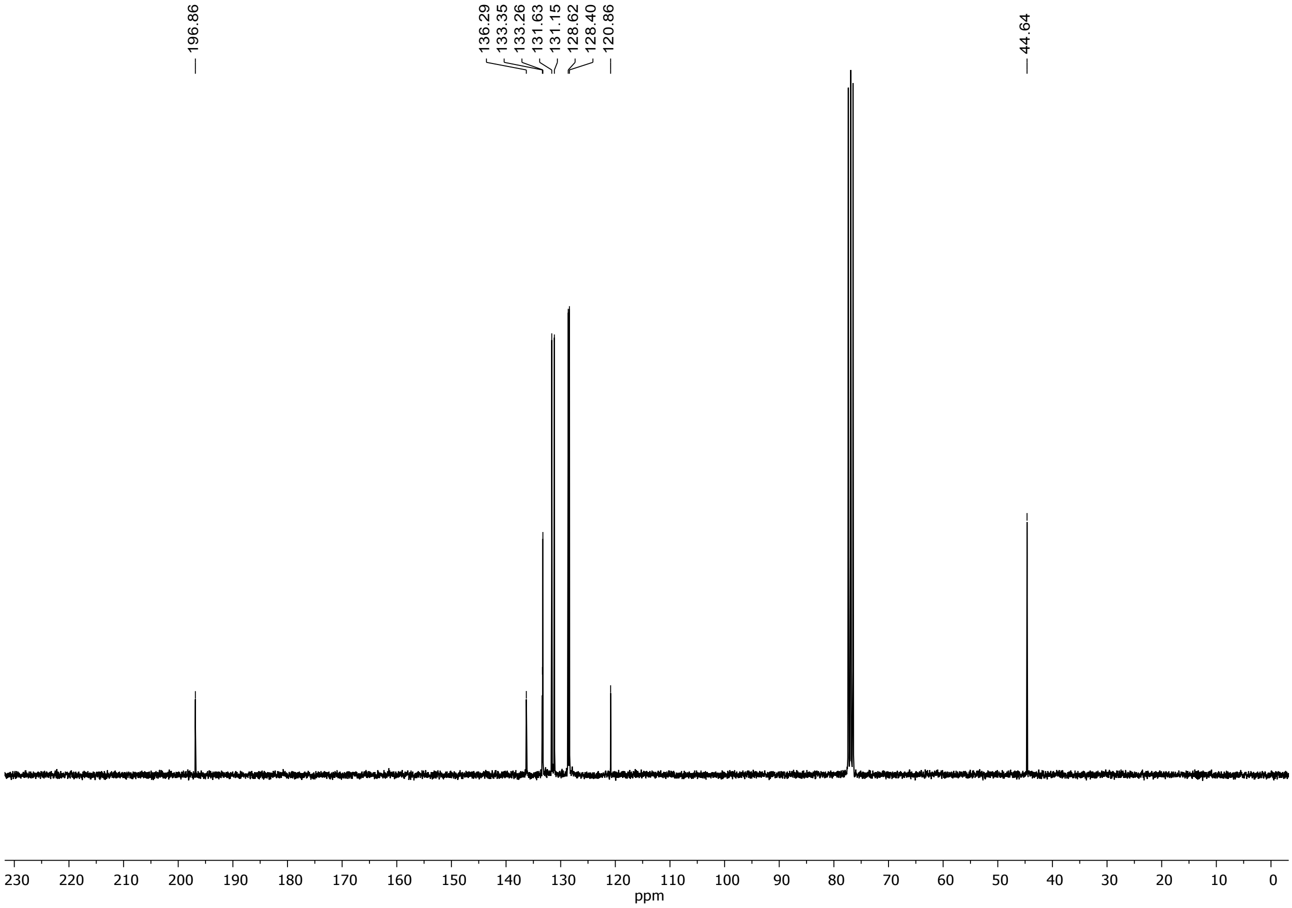

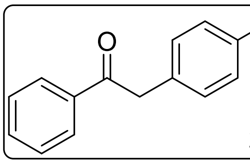

22 ^{1}H NMR (300 MHz, $CDCl_3$)

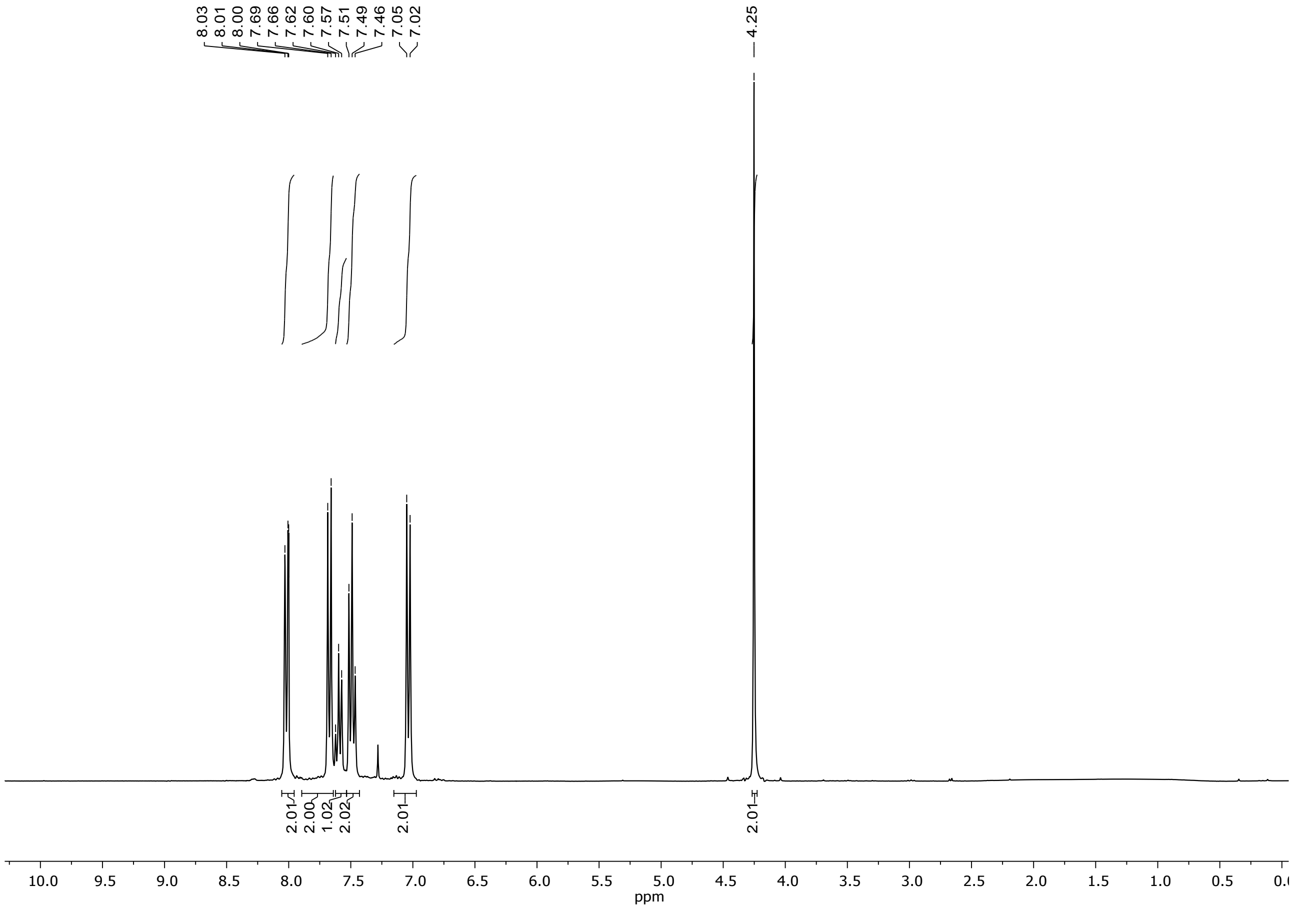

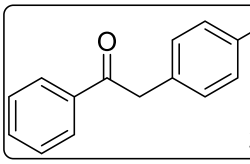

22 ^{13}C NMR (75 MHz, $CDCl_3$)

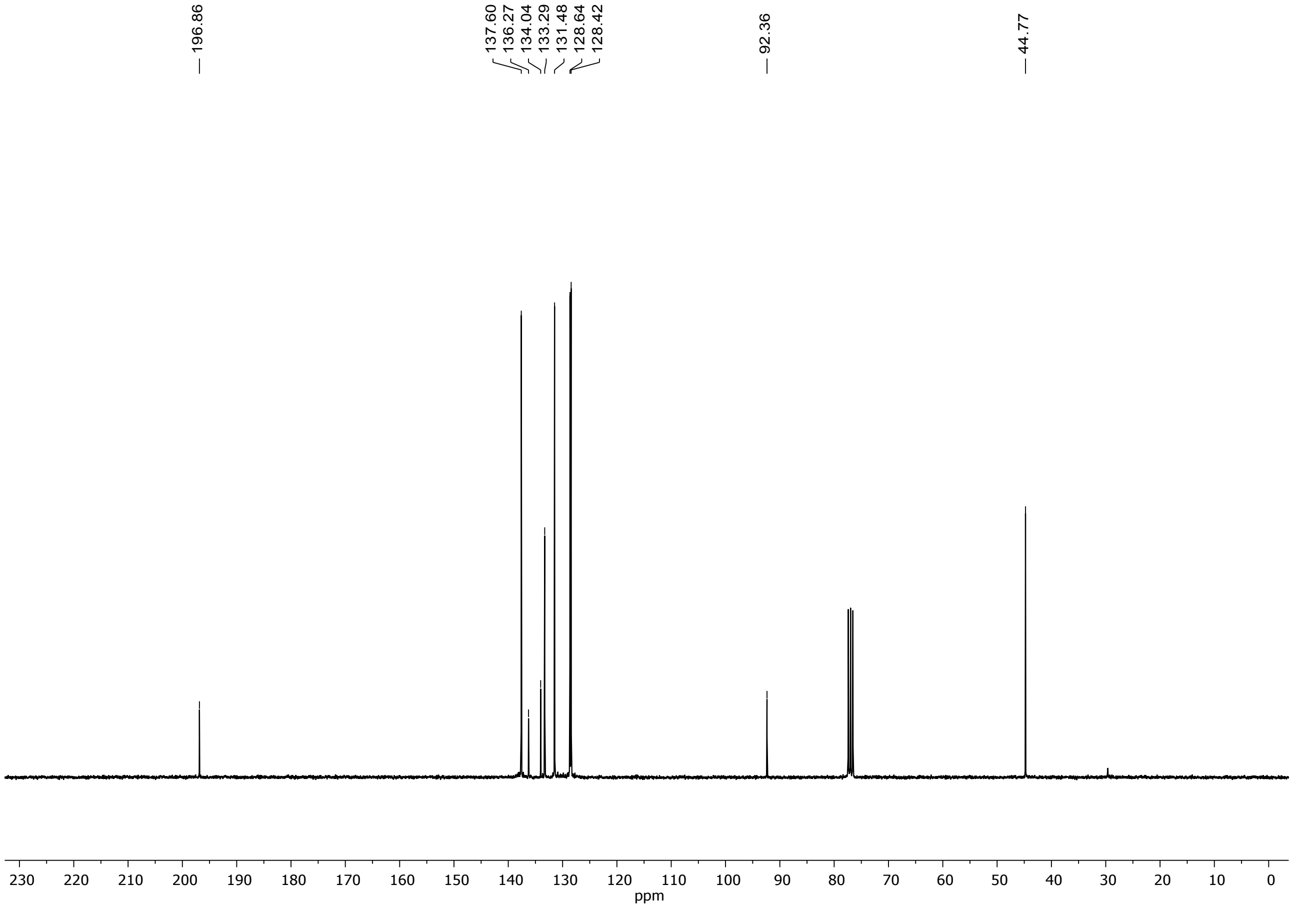

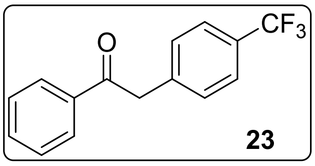

23

^{1}H NMR (300 MHz, $CDCl_3$)

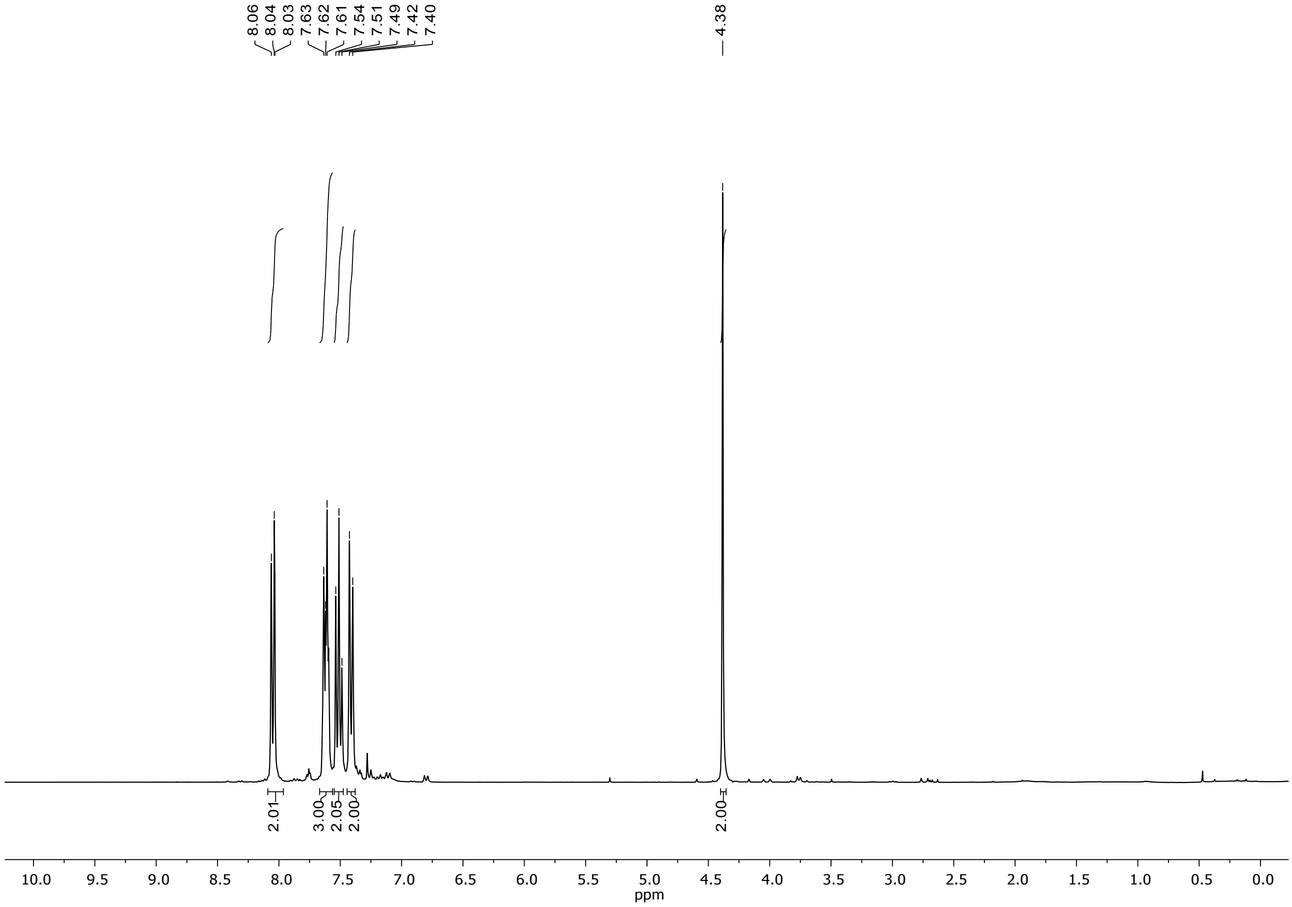

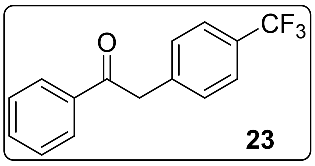

^{13}C NMR (75 MHz, $CDCl_3$)

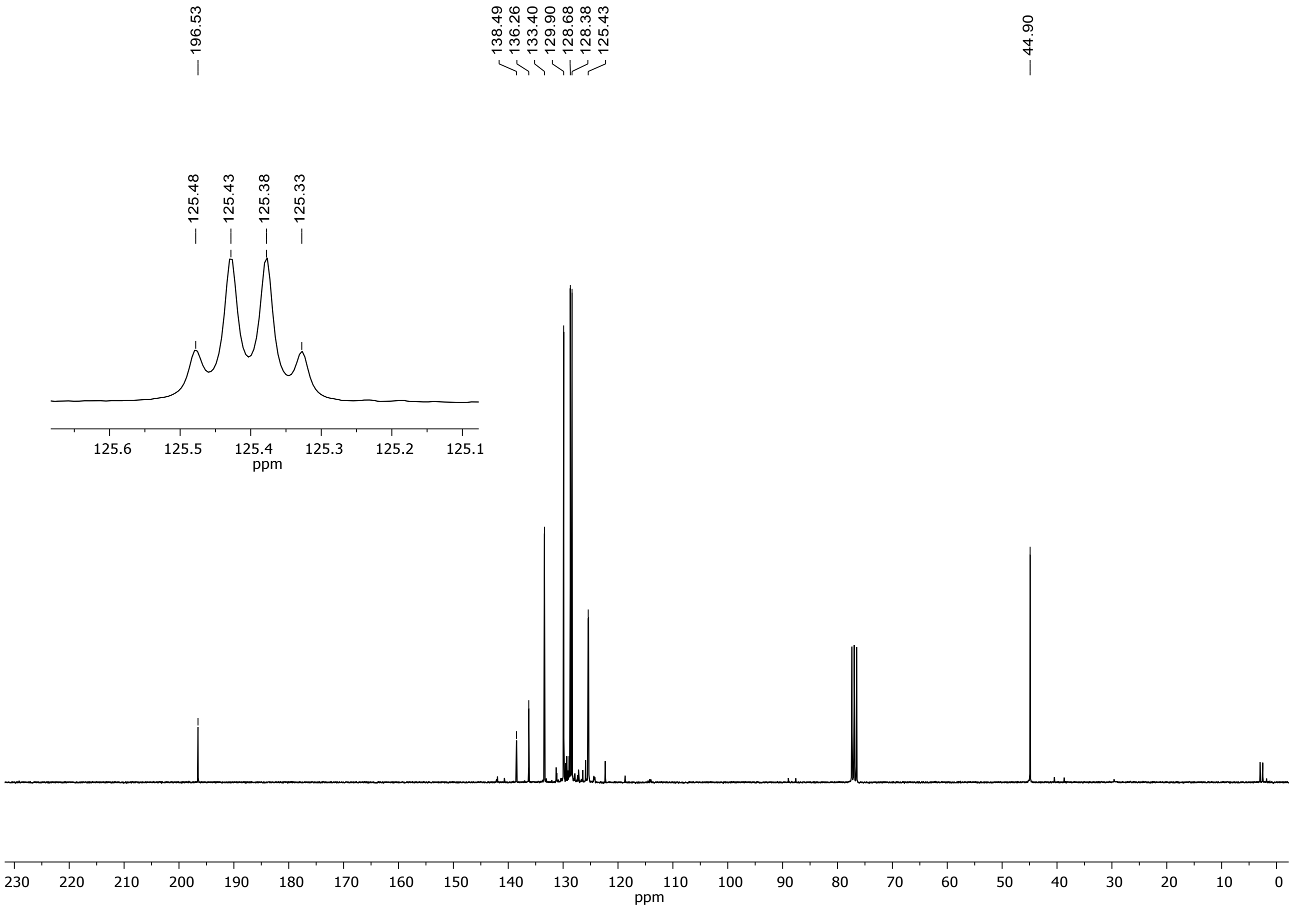

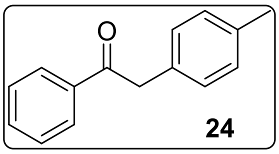

24

^{1}H NMR (300 MHz, $CDCl_3$)

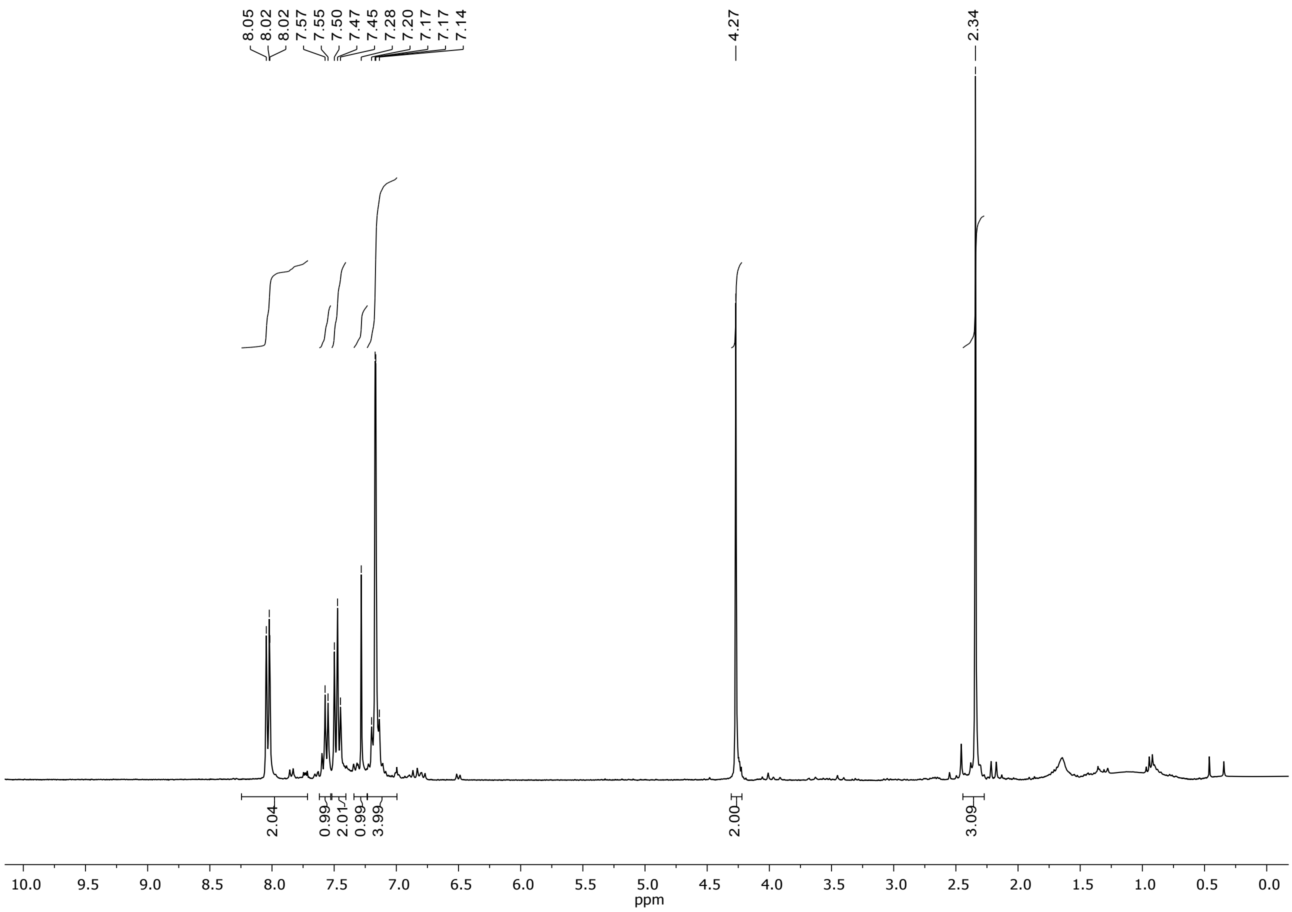

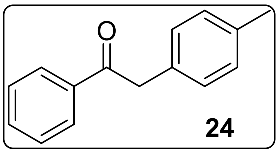

^{13}C NMR (75 MHz, $CDCl_3$)

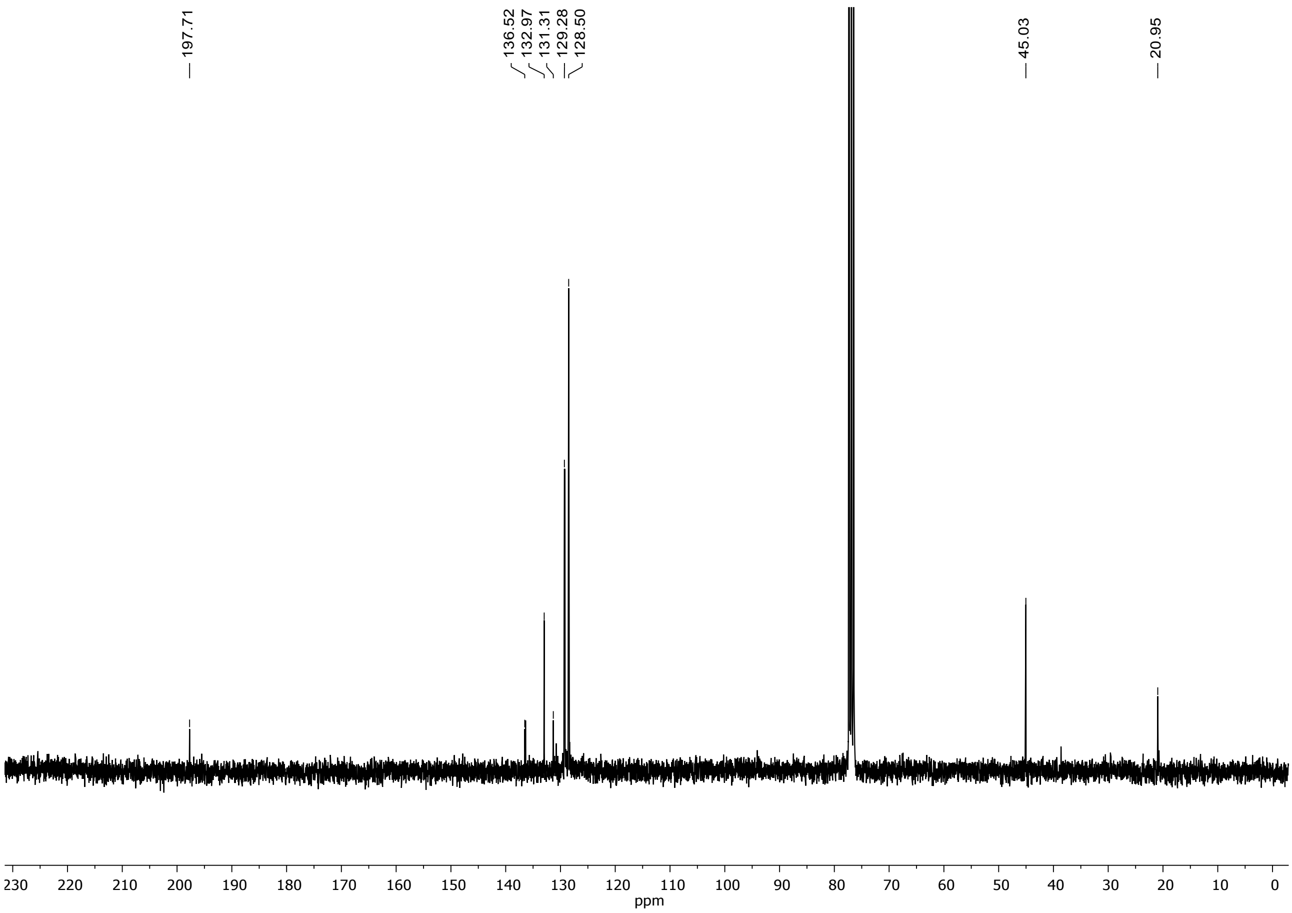

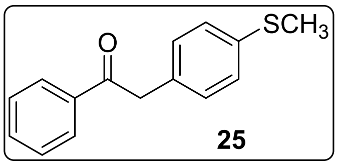

25

^{1}H NMR (300 MHz, $CDCl_3$)

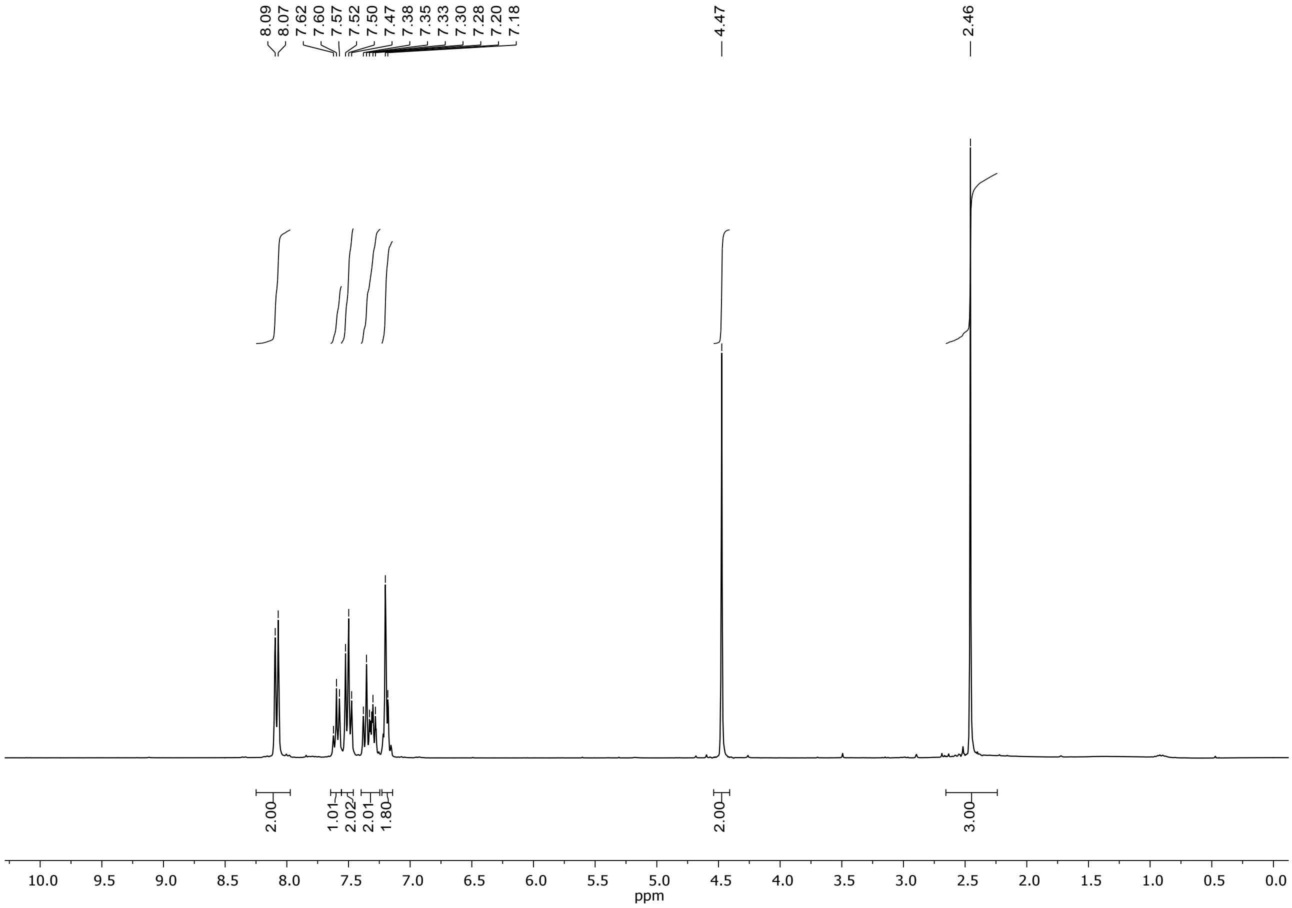

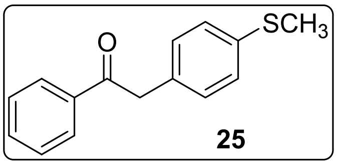

25

^{13}C NMR (75 MHz, $CDCl_3$)

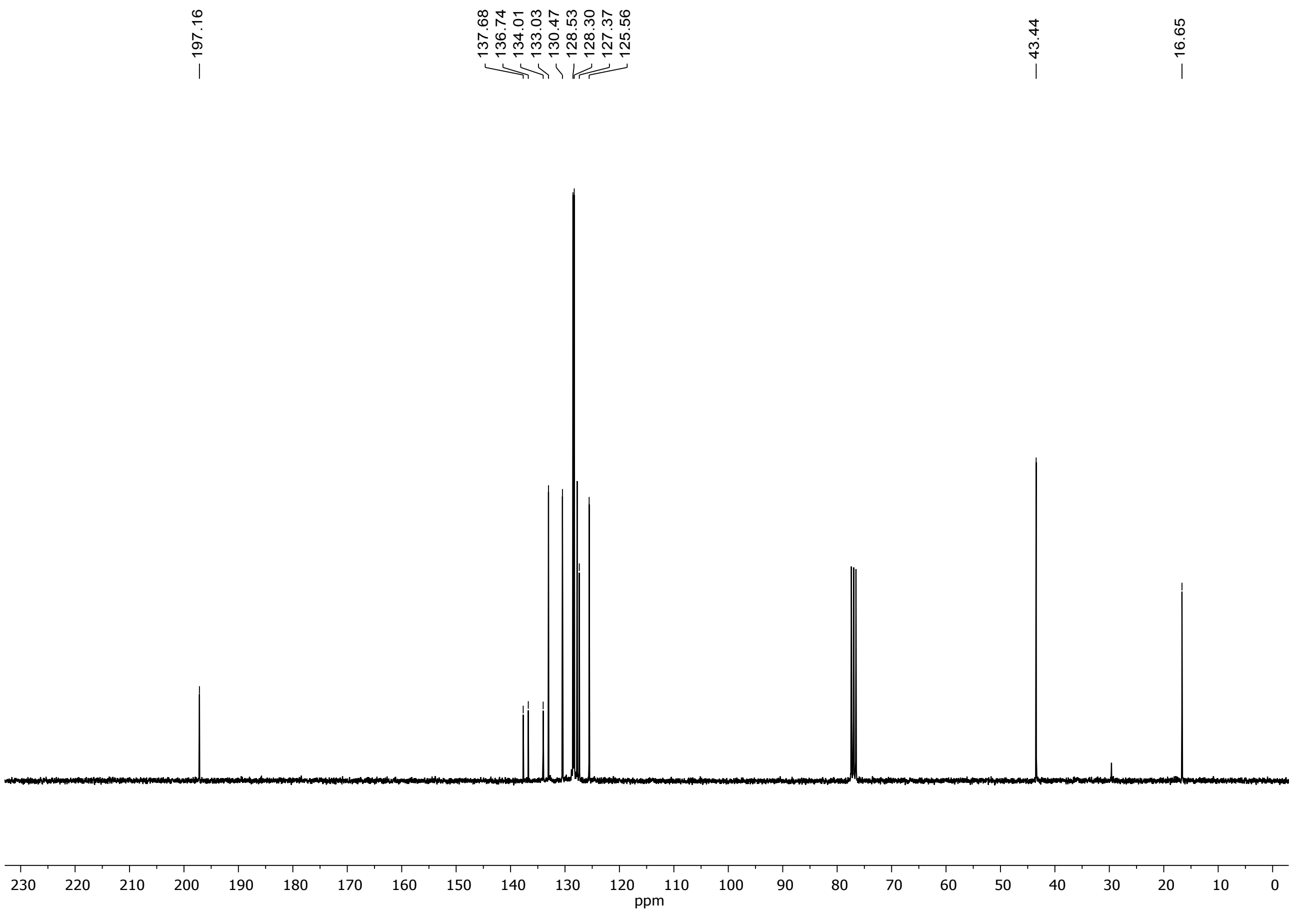

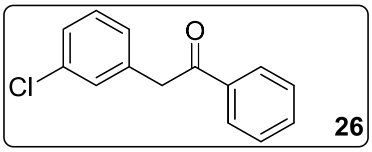

26 ^1H NMR (300 MHz, $CDCl_3$)

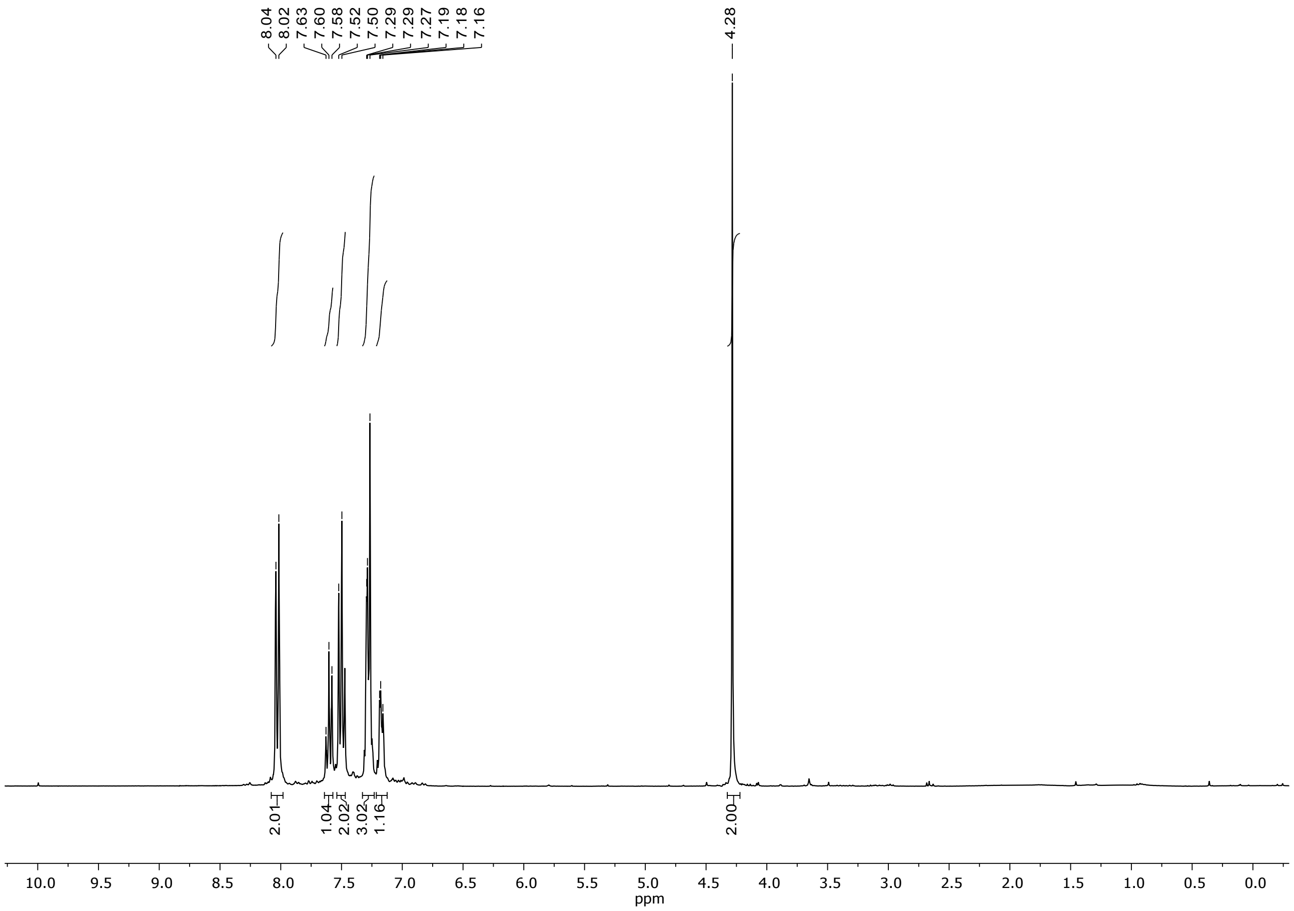

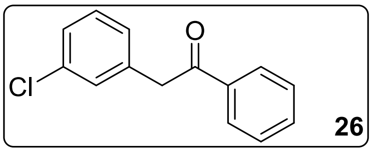

26 ^{13}C NMR (75 MHz, $CDCl_3$)

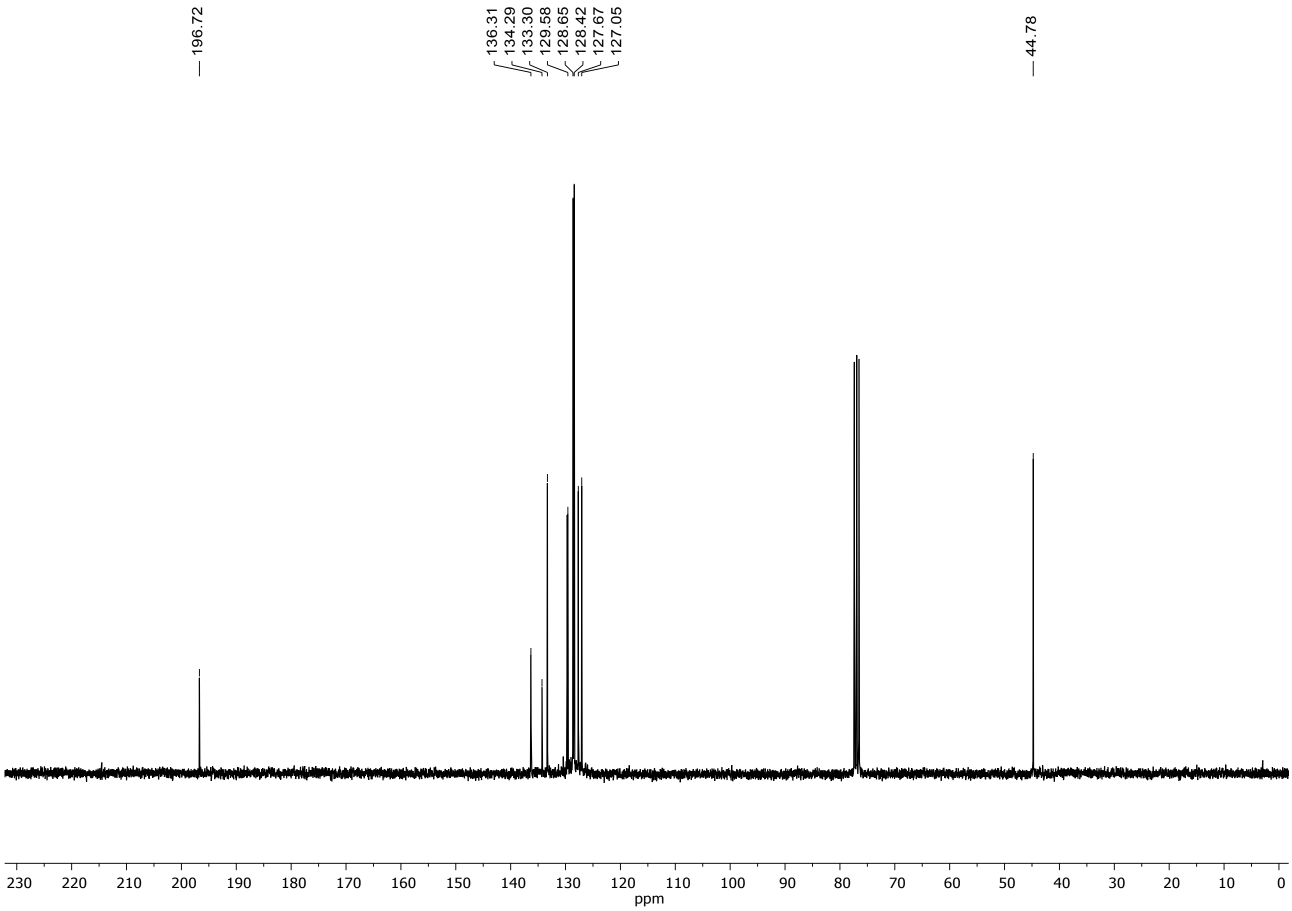

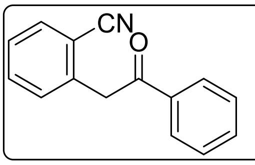

27

^{1}H NMR (300 MHz, $CDCl_3$)

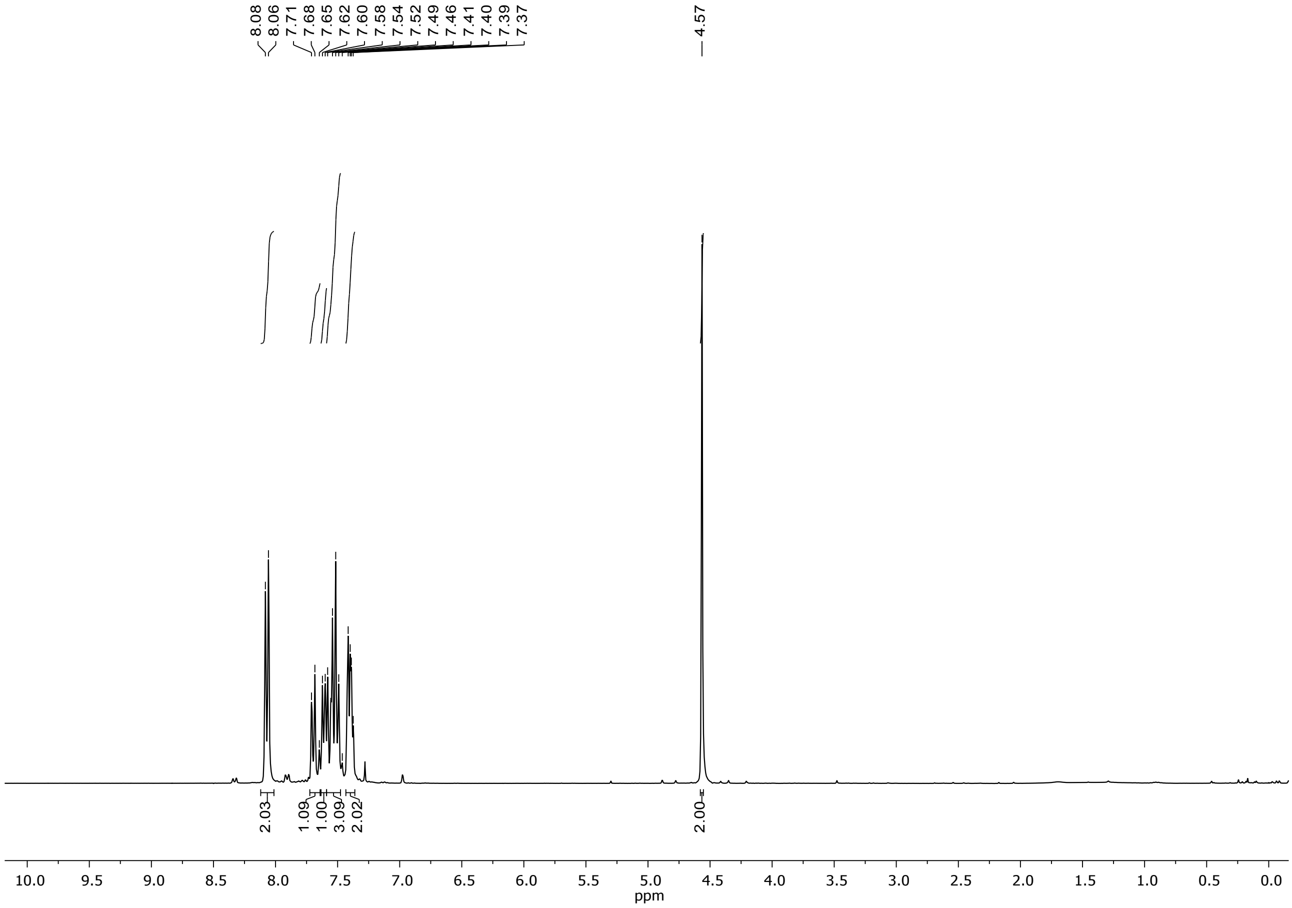

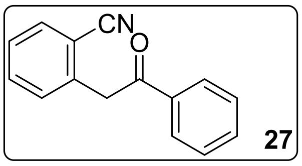

27 ^{13}C NMR (75 MHz, $CDCl_3$)

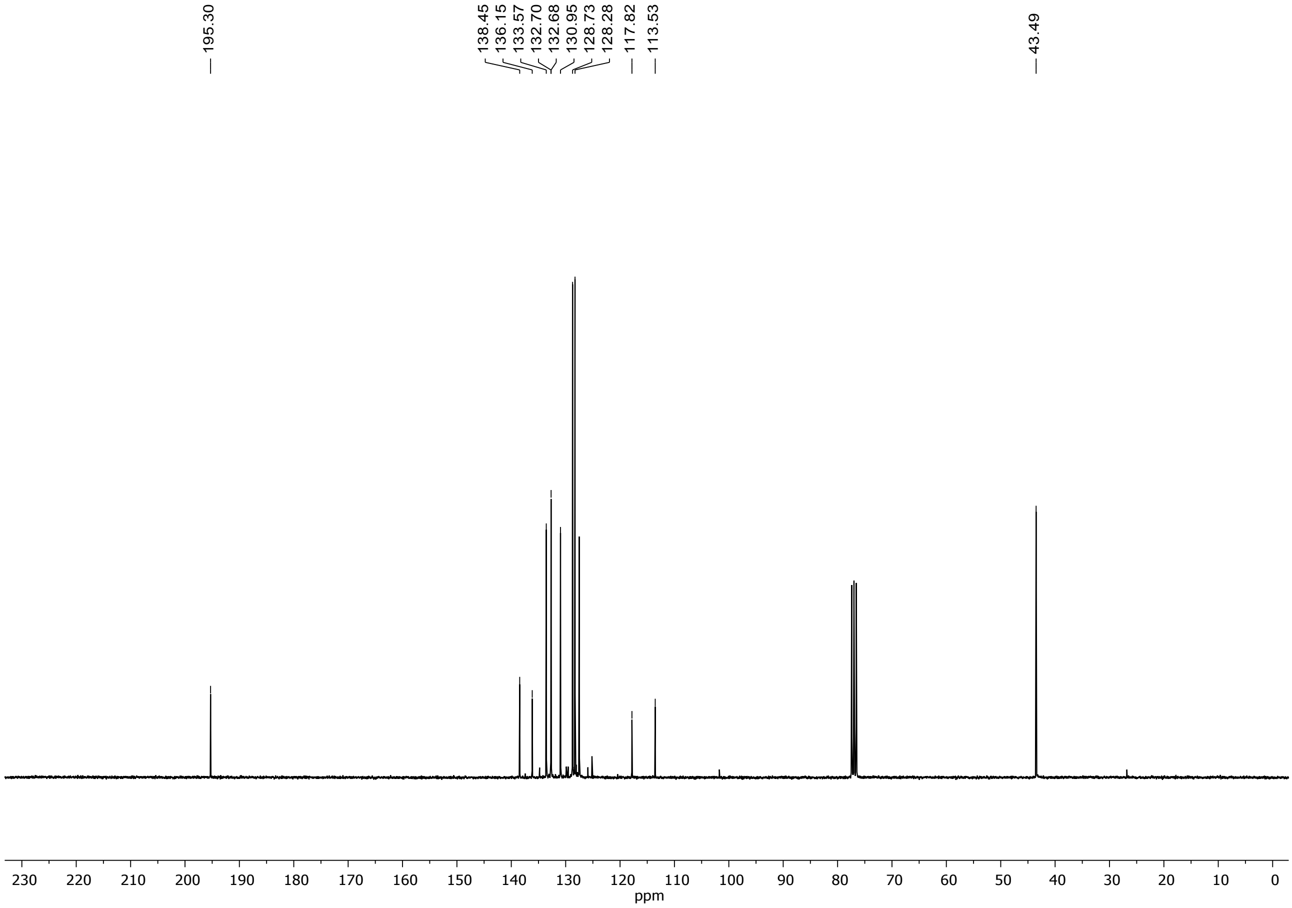

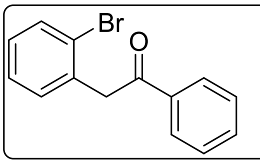

28 ^{1}H NMR (300 MHz, $CDCl_3$)

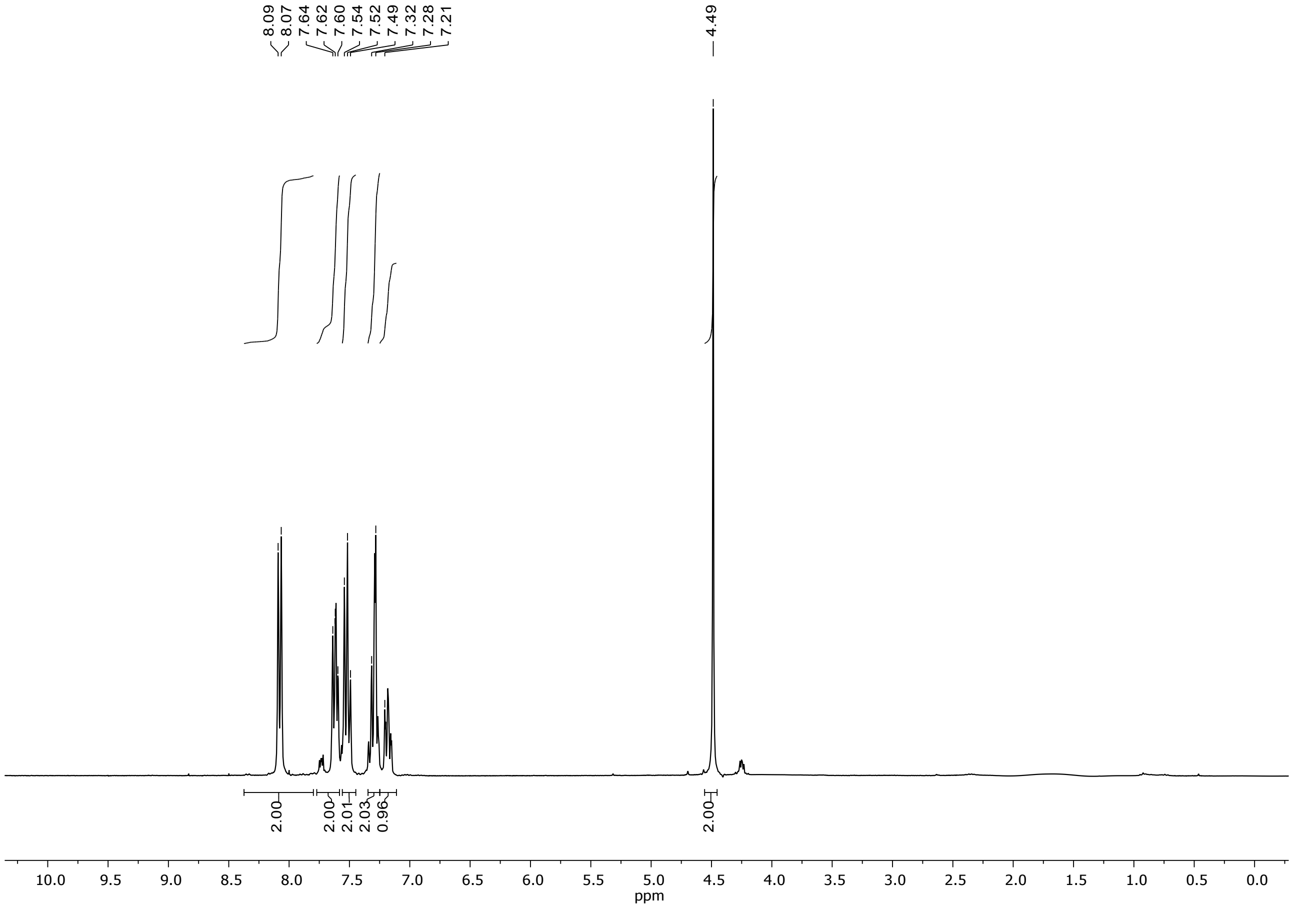

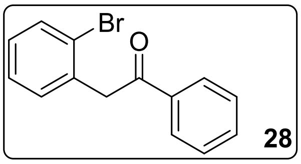

^{13}C NMR (75 MHz, $CDCl_3$)

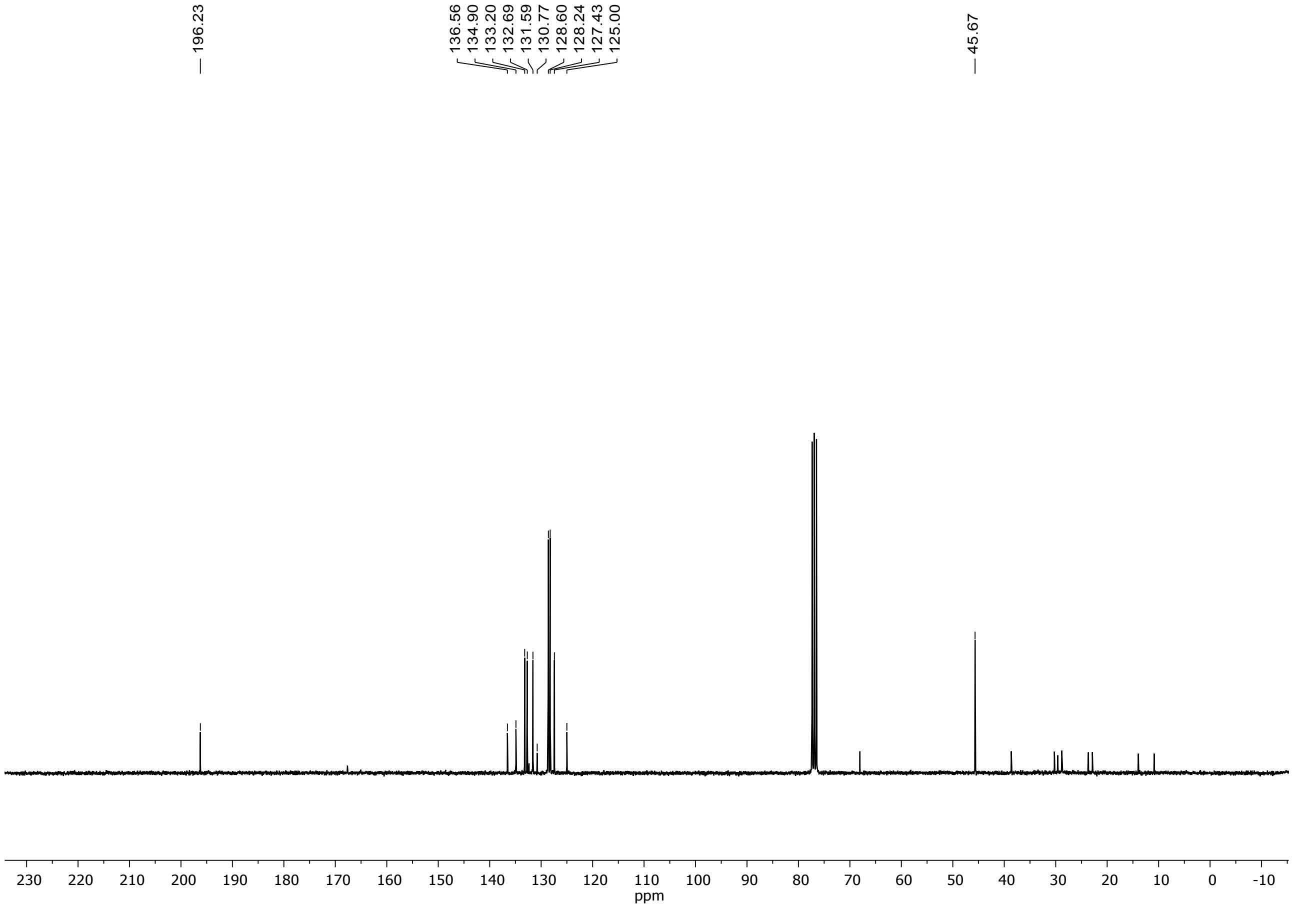

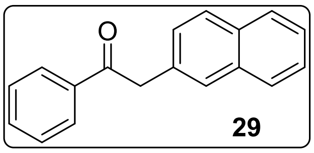

29

^{1}H NMR (300 MHz, $CDCl_3$)

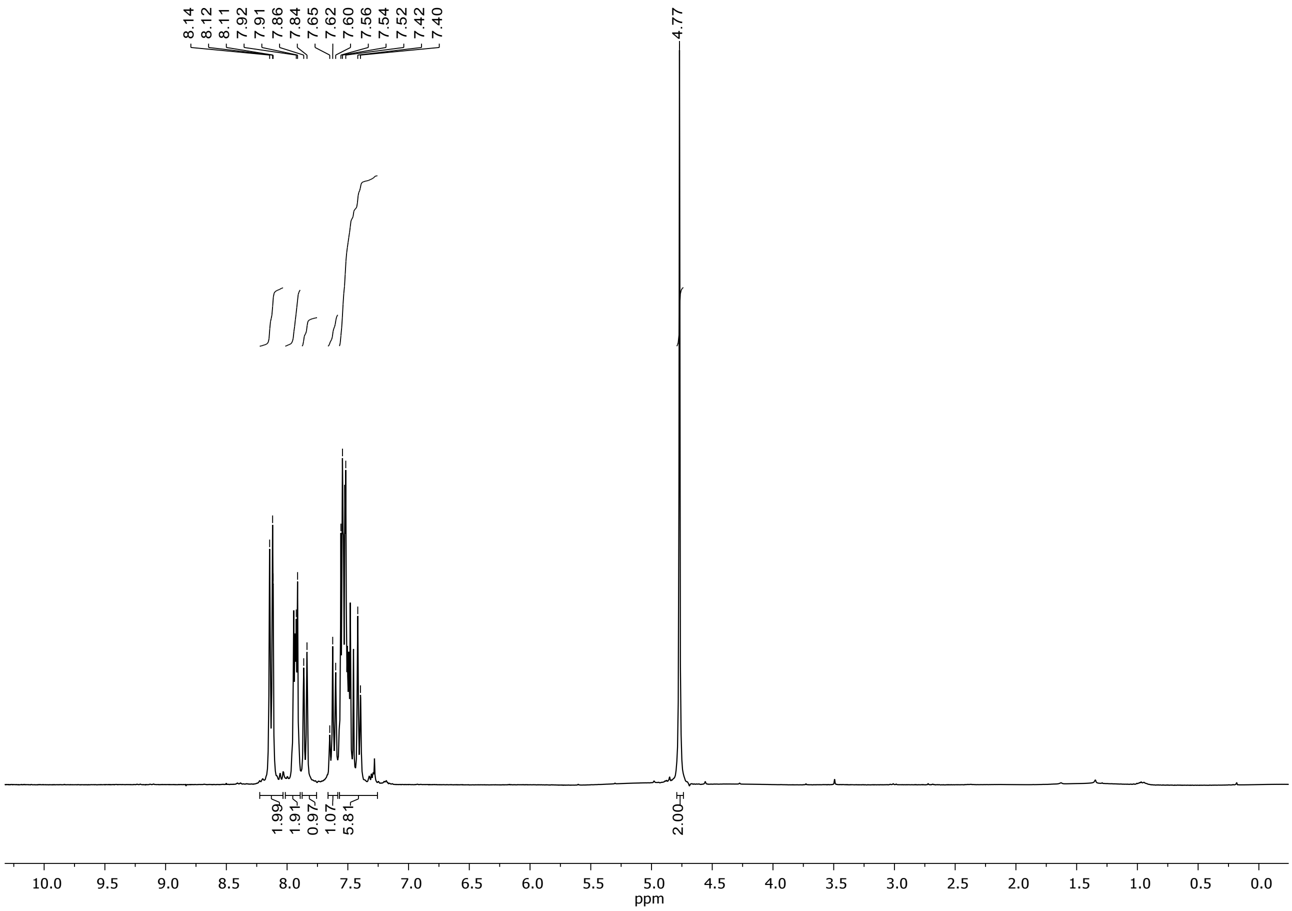

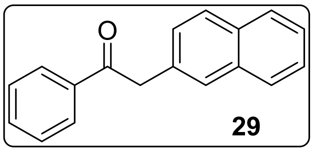
29

^{13}C NMR (75 MHz, $CDCl_3$)

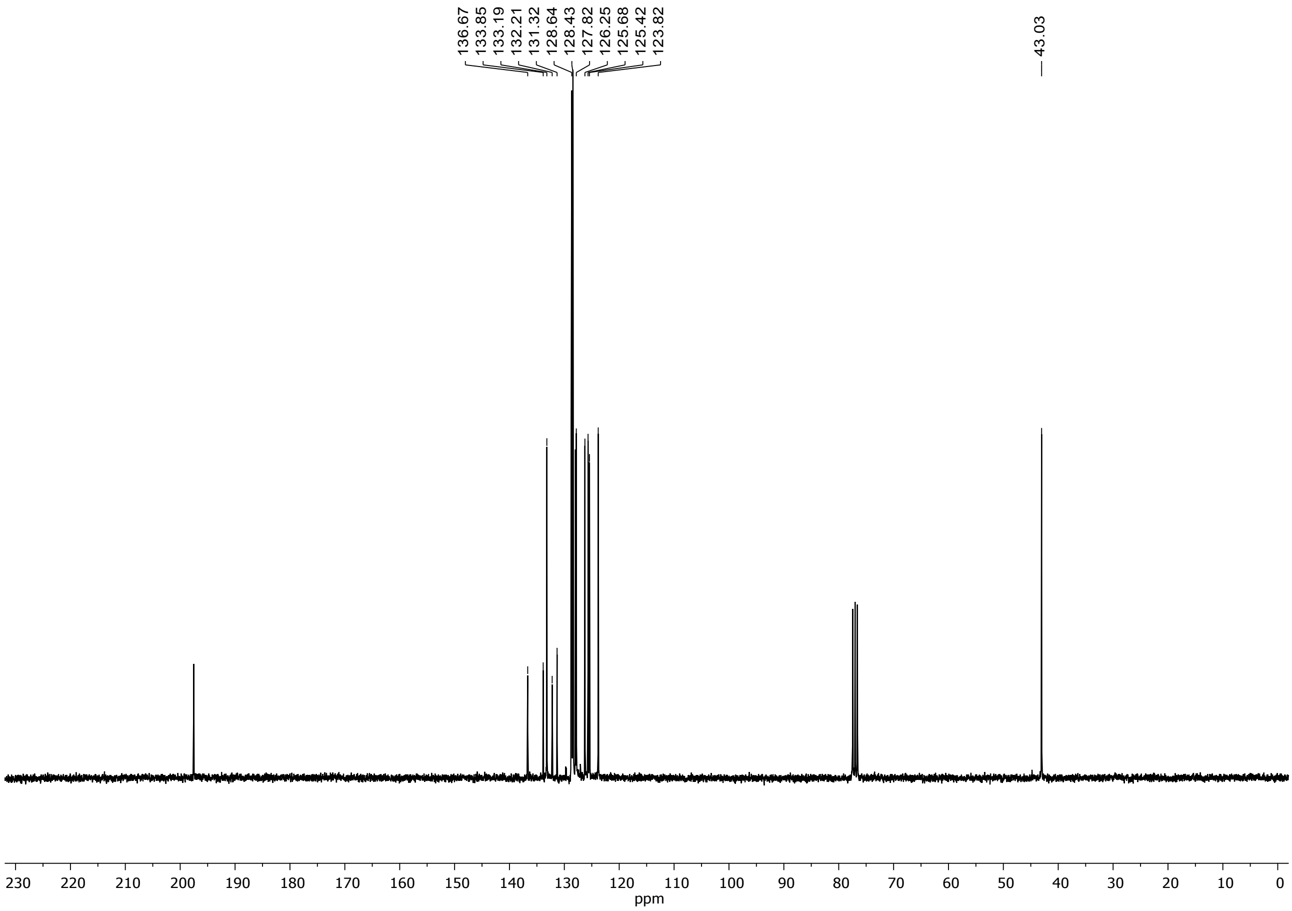

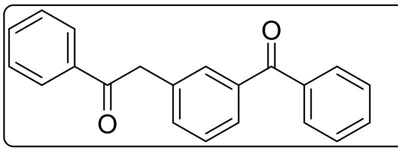

30

^{1}H NMR (300 MHz, $CDCl_3$)

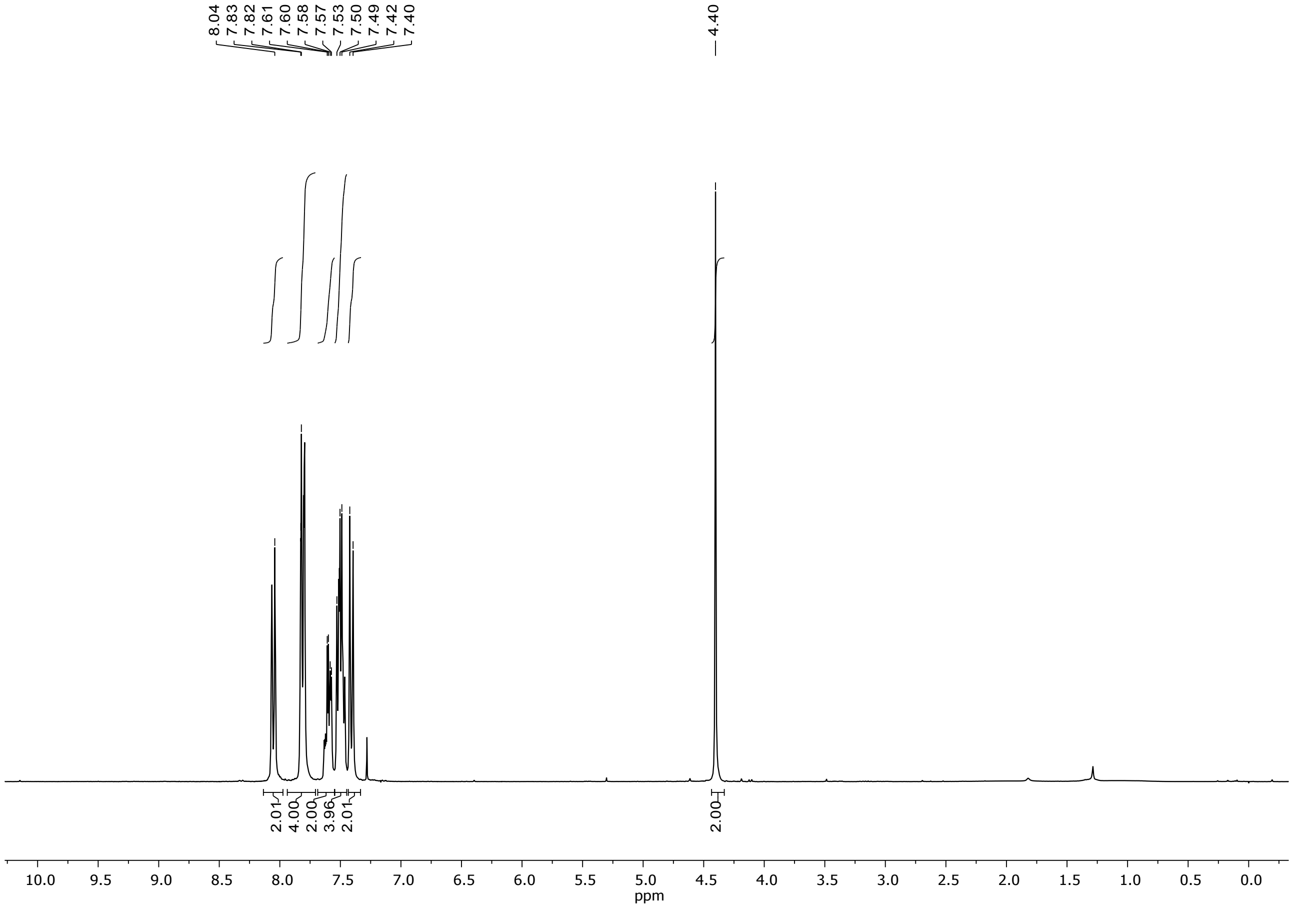

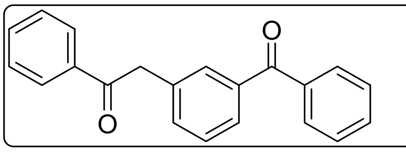

30 ^{13}C NMR (75 MHz, $CDCl_3$)

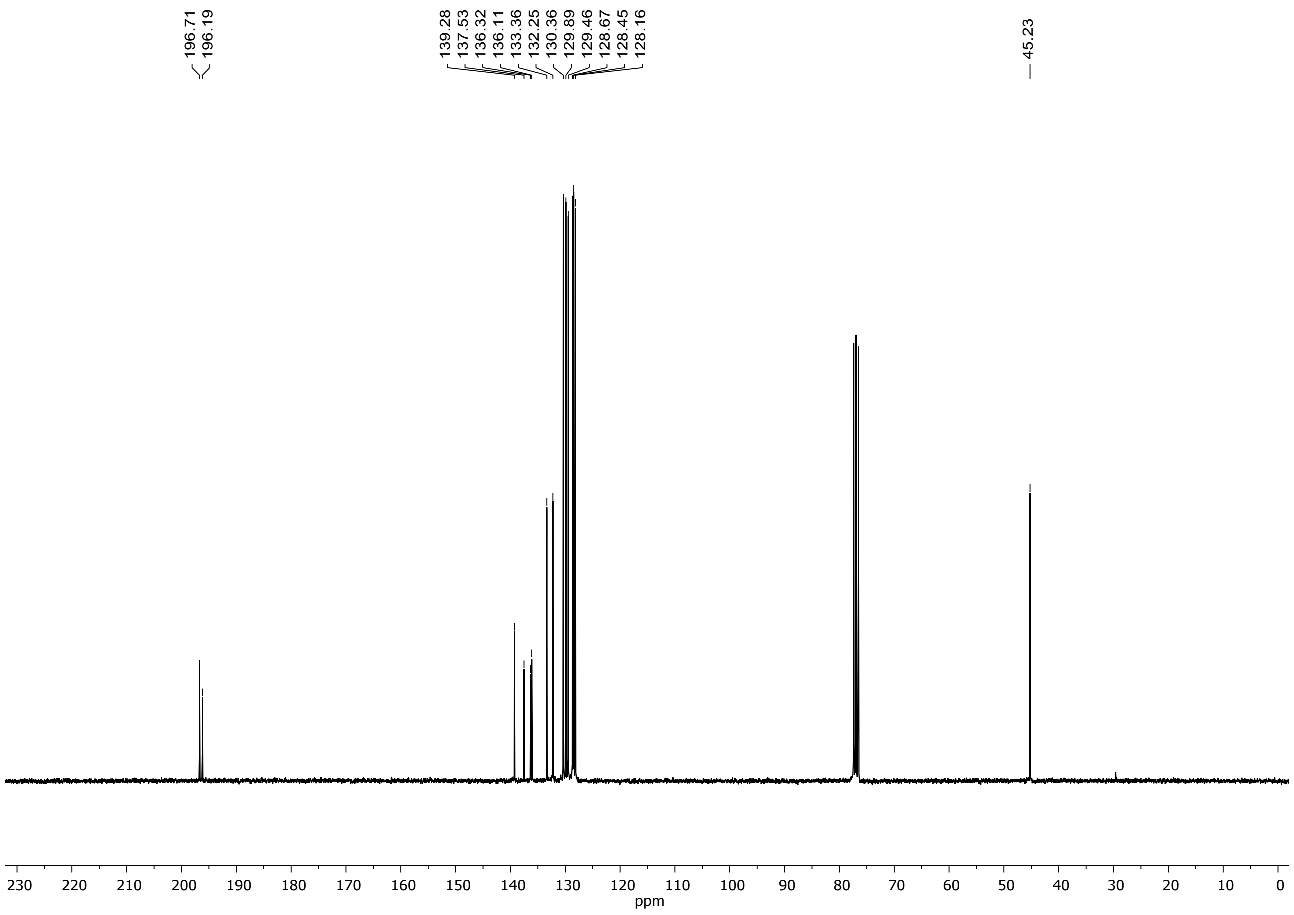

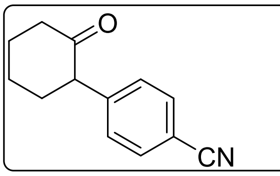

31 ^{1}H NMR (300 MHz, $CDCl_3$)

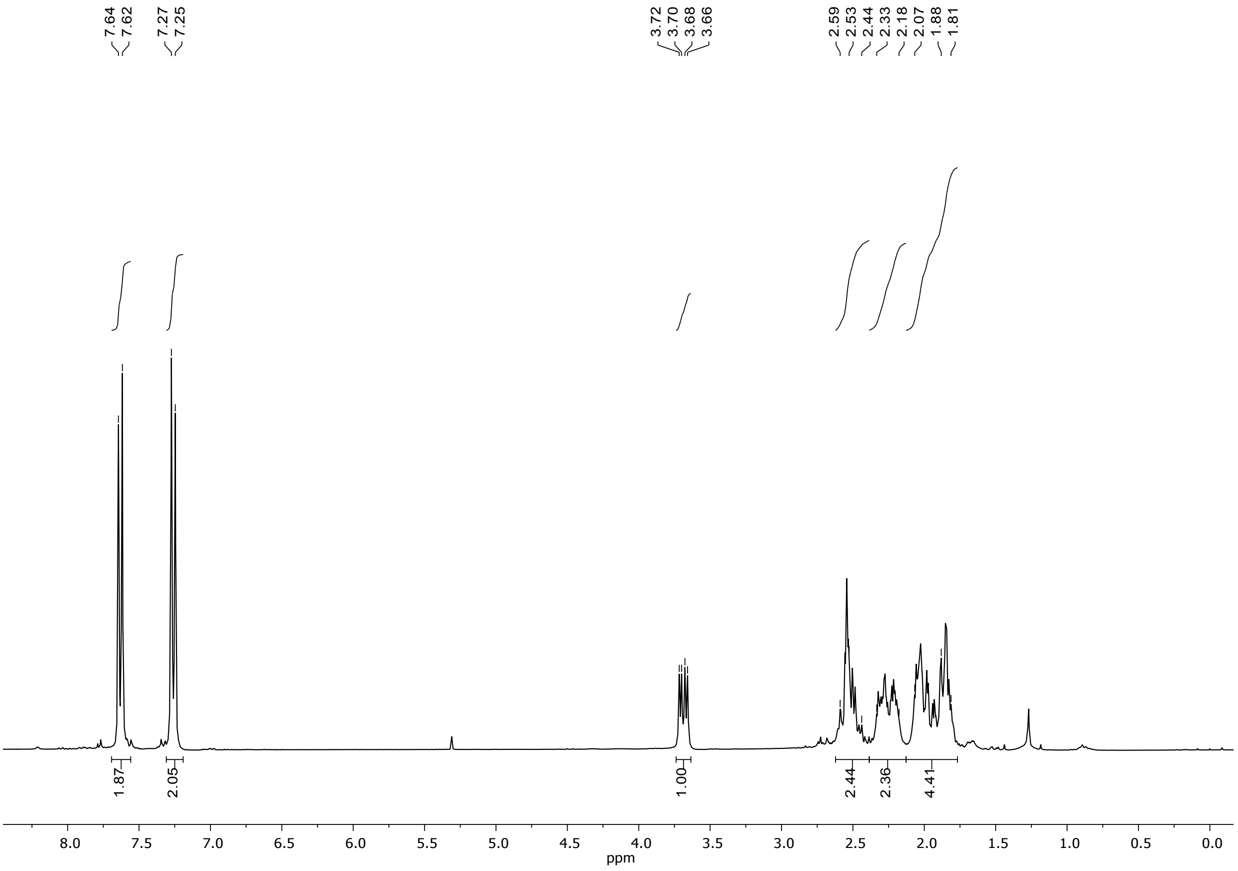

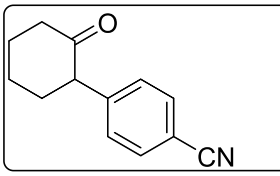

31

^{13}C NMR (75 MHz, $CDCl_3$)

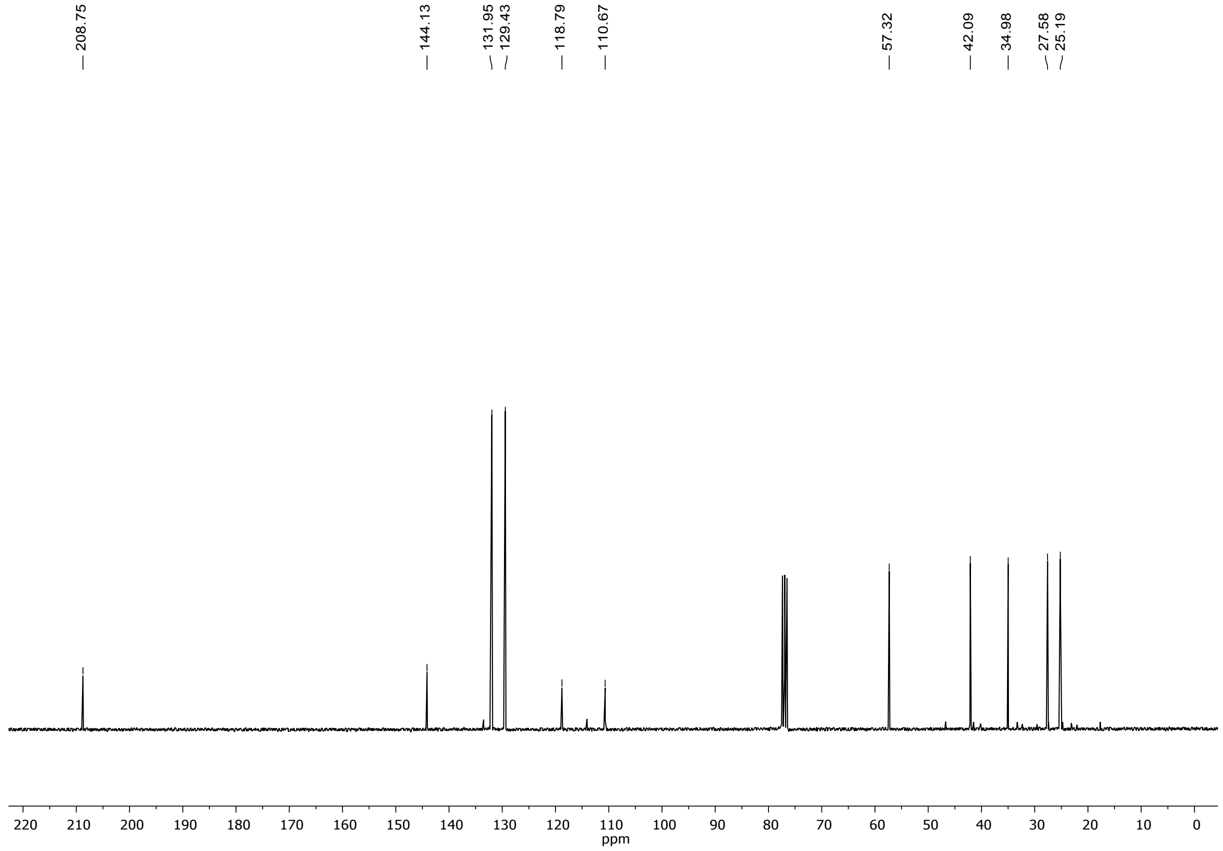

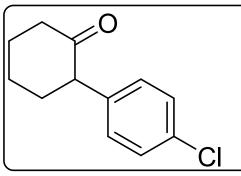

32

^{1}H NMR (300 MHz, $CDCl_3$)

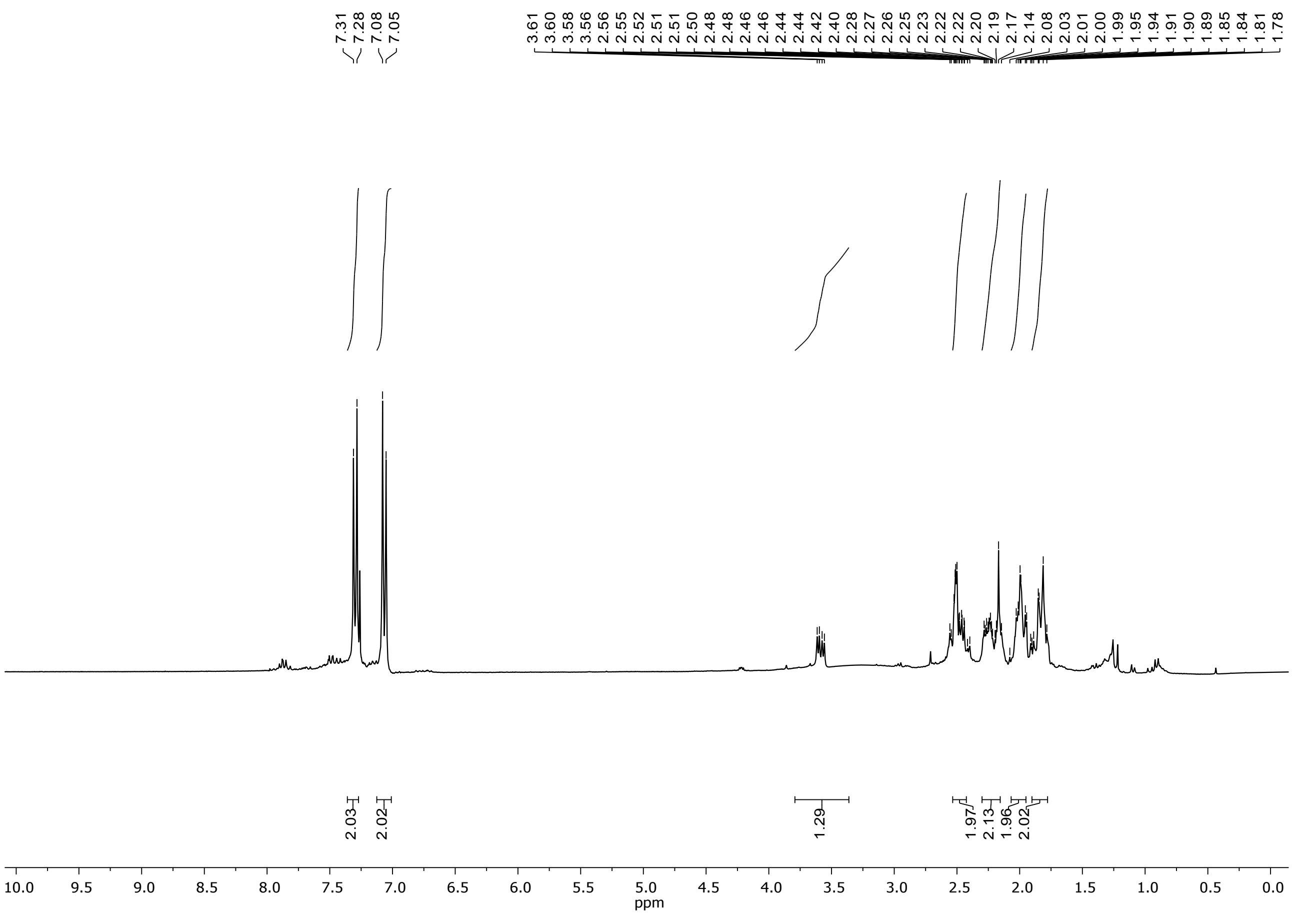

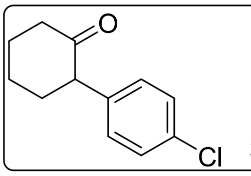

32

^{13}C NMR (75 MHz, $CDCl_3$)

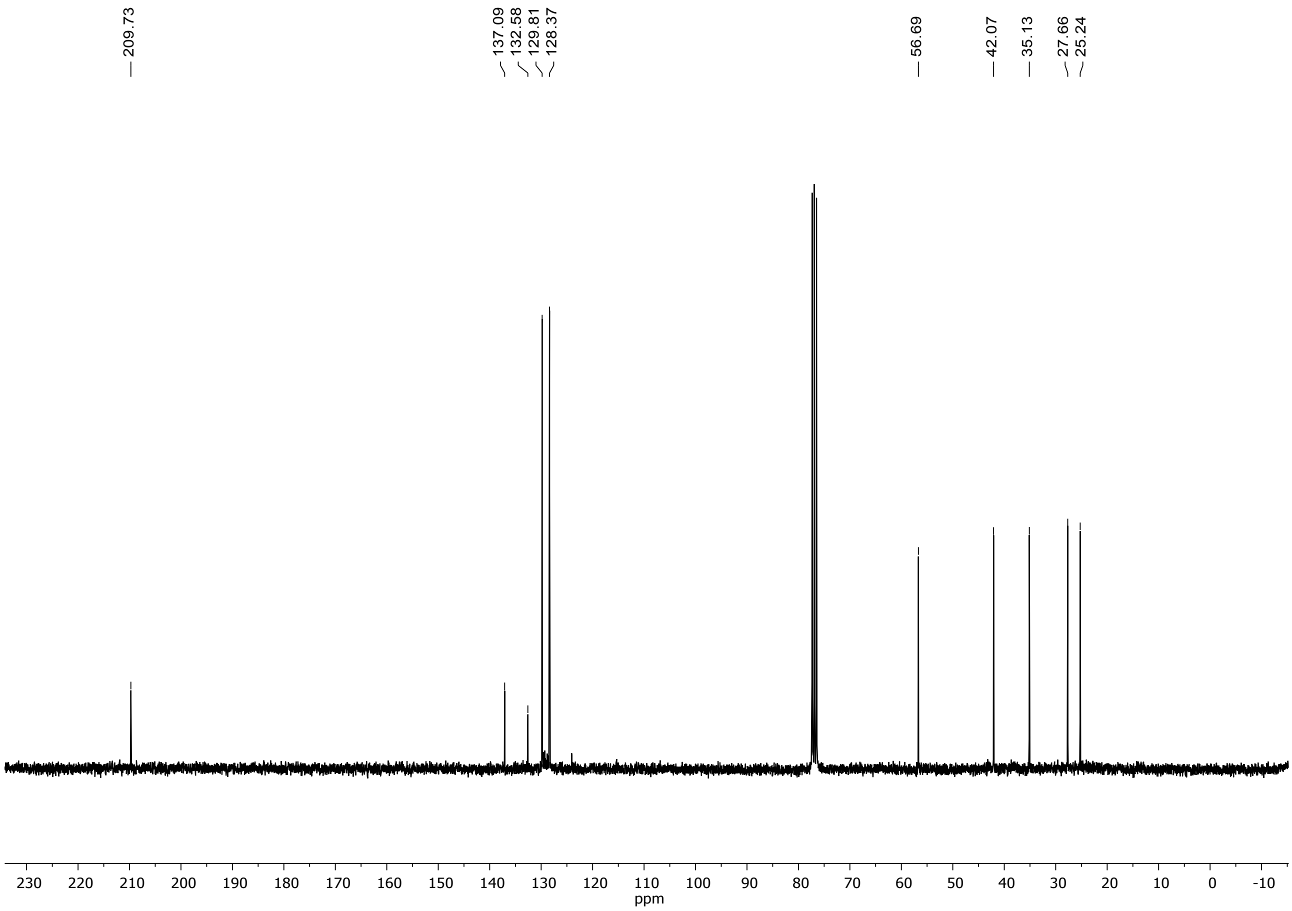

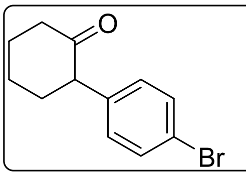

33 ^{1}H NMR (300 MHz, $CDCl_3$)

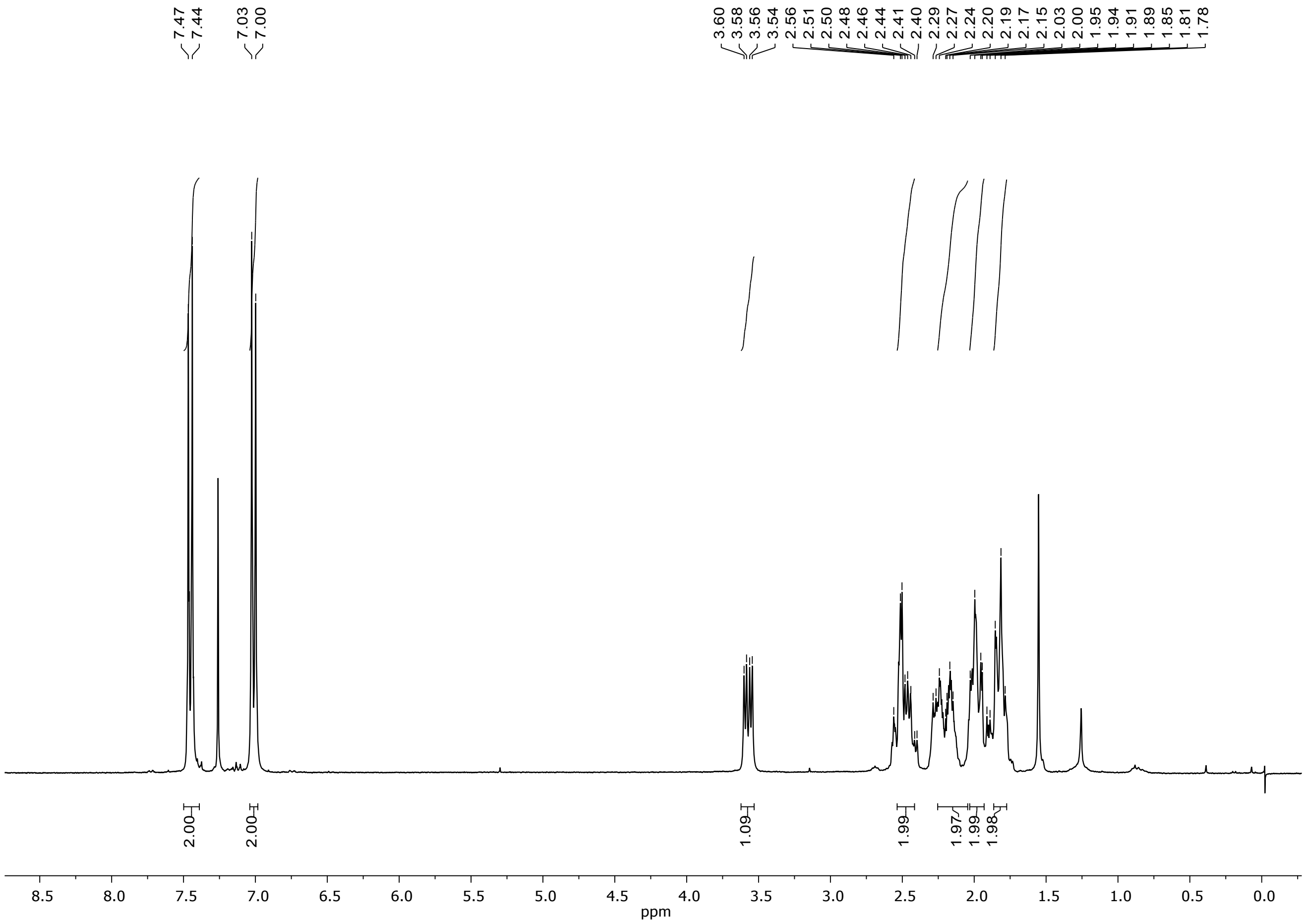

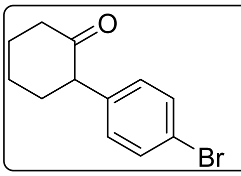

33

^{13}C NMR (75 MHz, $CDCl_3$)

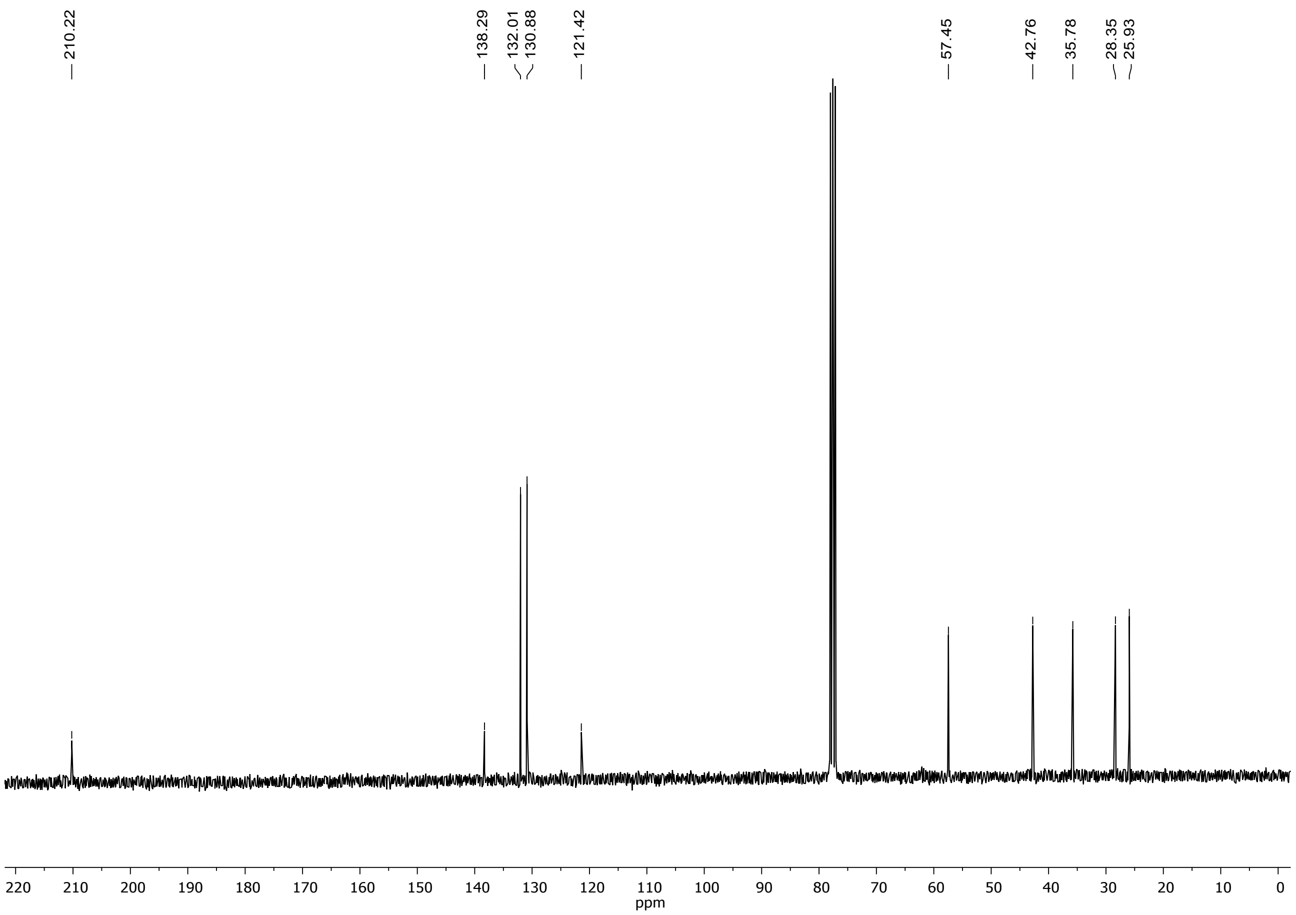

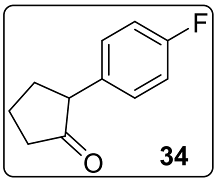

^{1}H NMR (300 MHz, $CDCl_3$)

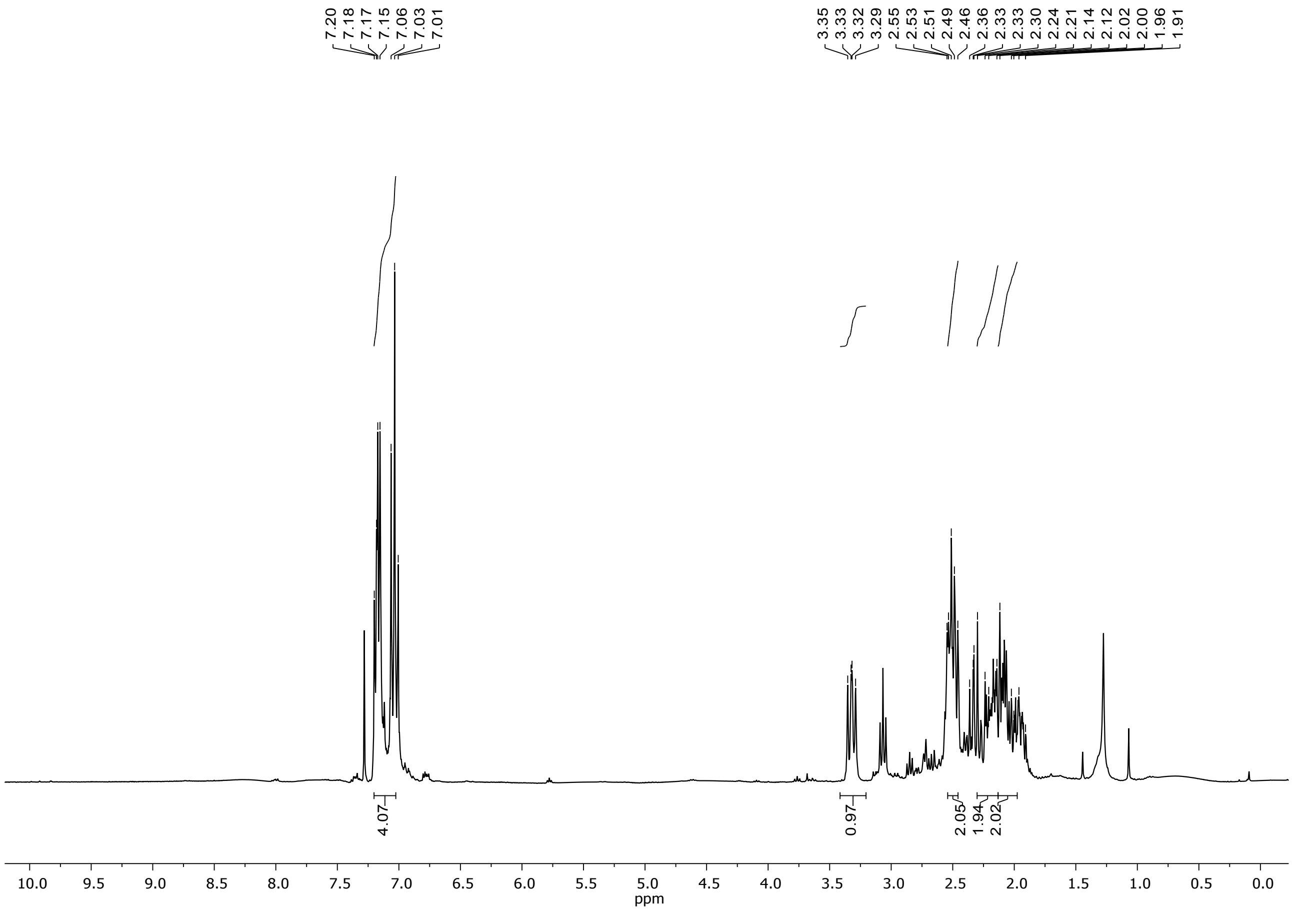

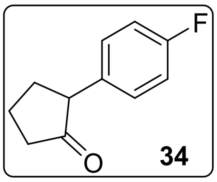

34

^{13}C NMR (75 MHz, $CDCl_3$)

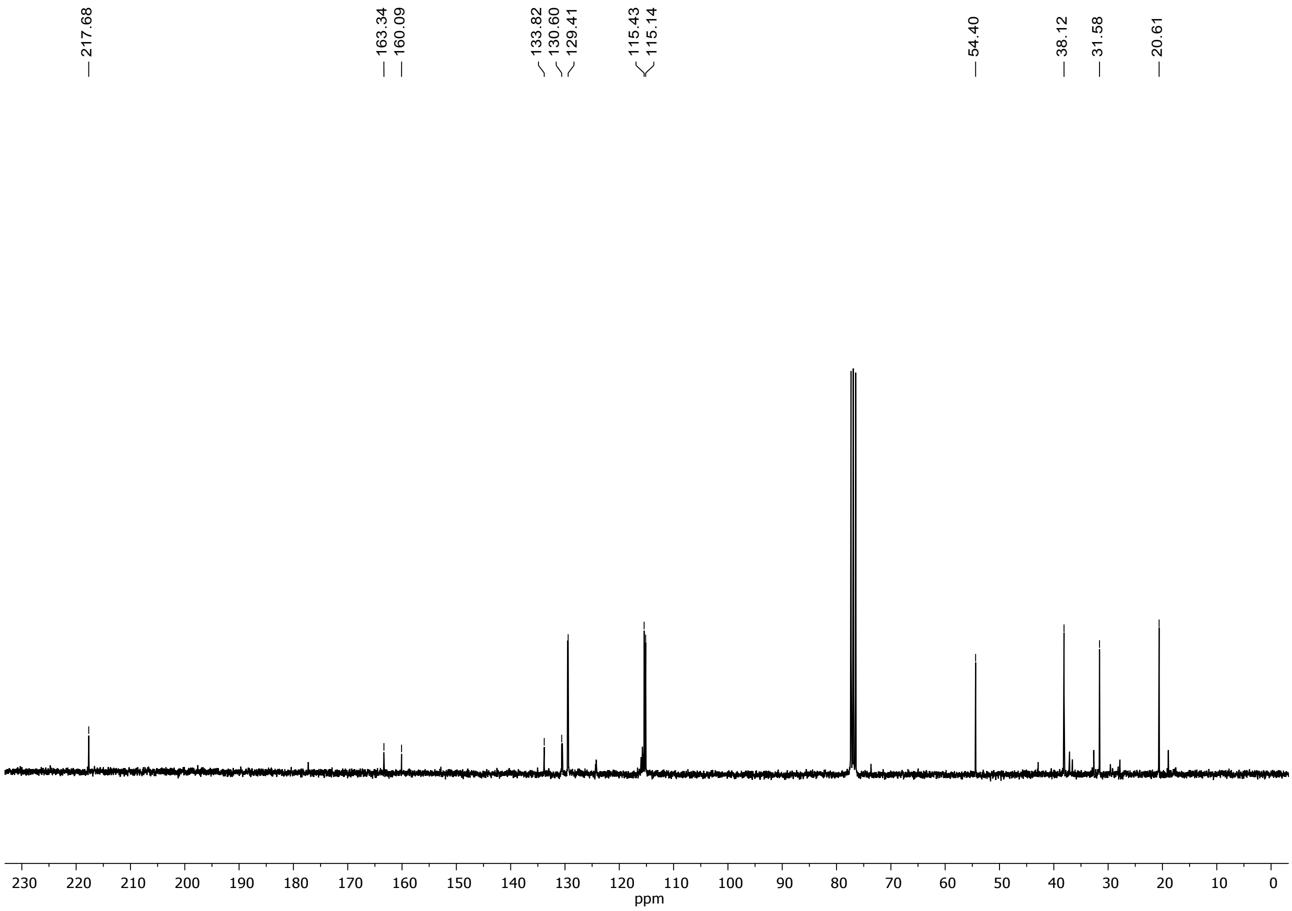

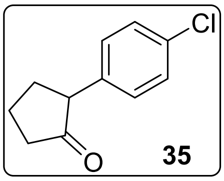

35

^{1}H NMR (300 MHz, $CDCl_3$)

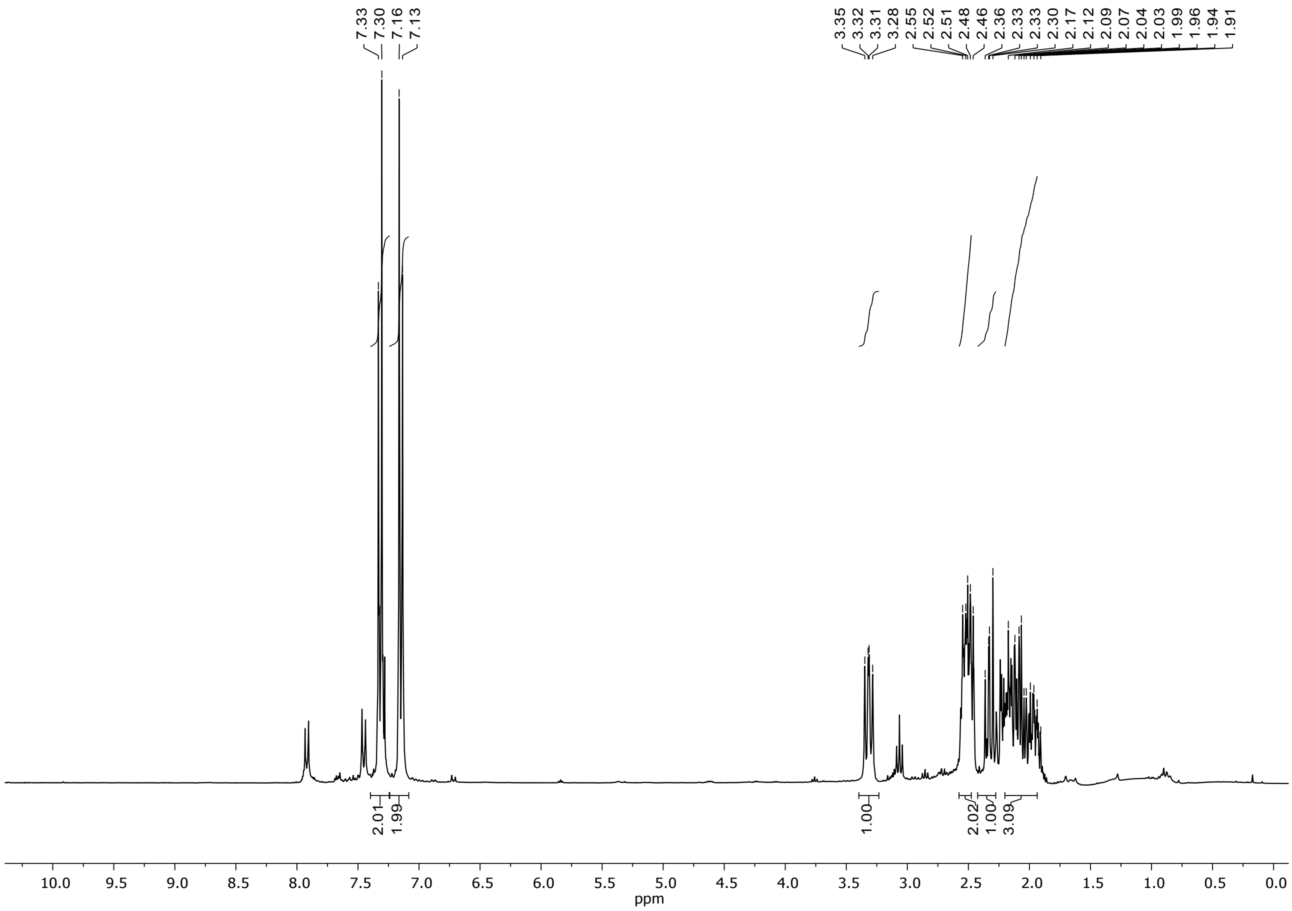

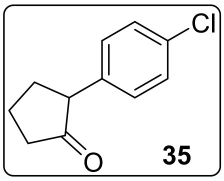

^{13}C NMR (75 MHz, $CDCl_3$)

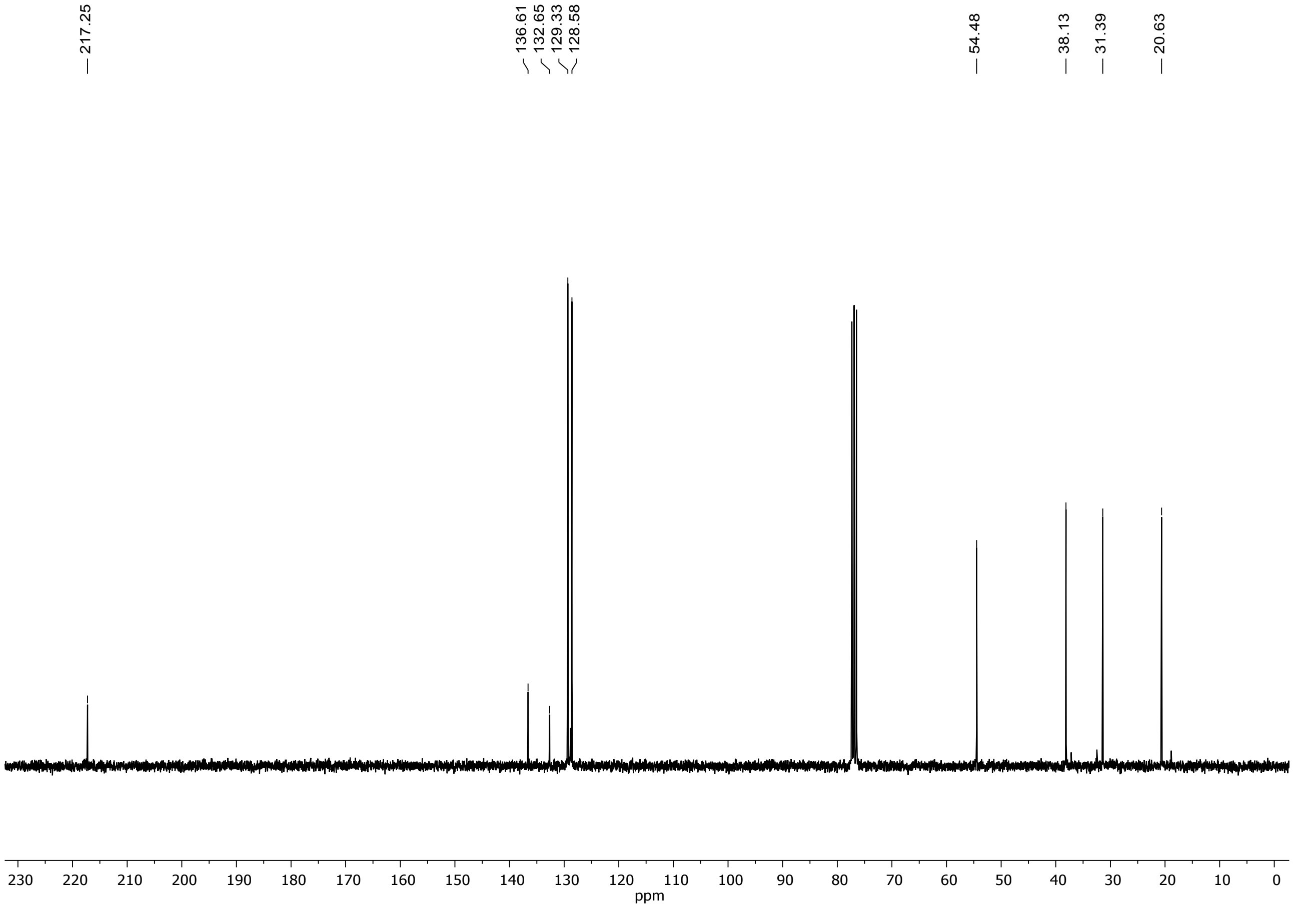

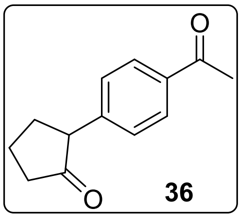

36

^{1}H NMR (300 MHz, $CDCl_3$)

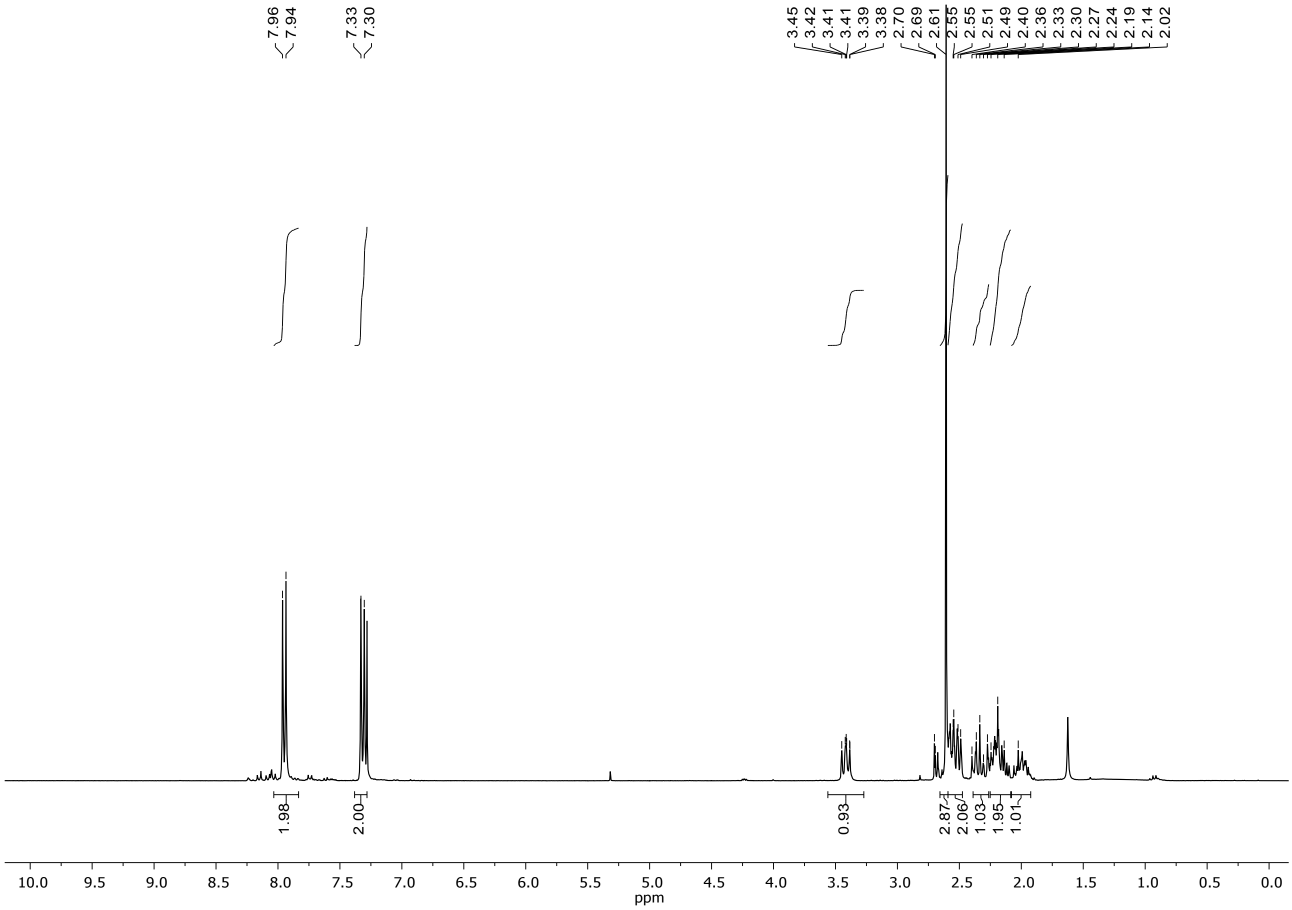

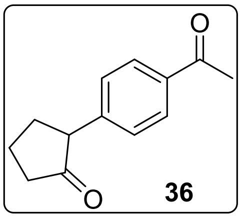

36

^{13}C NMR (75 MHz, $CDCl_3$)

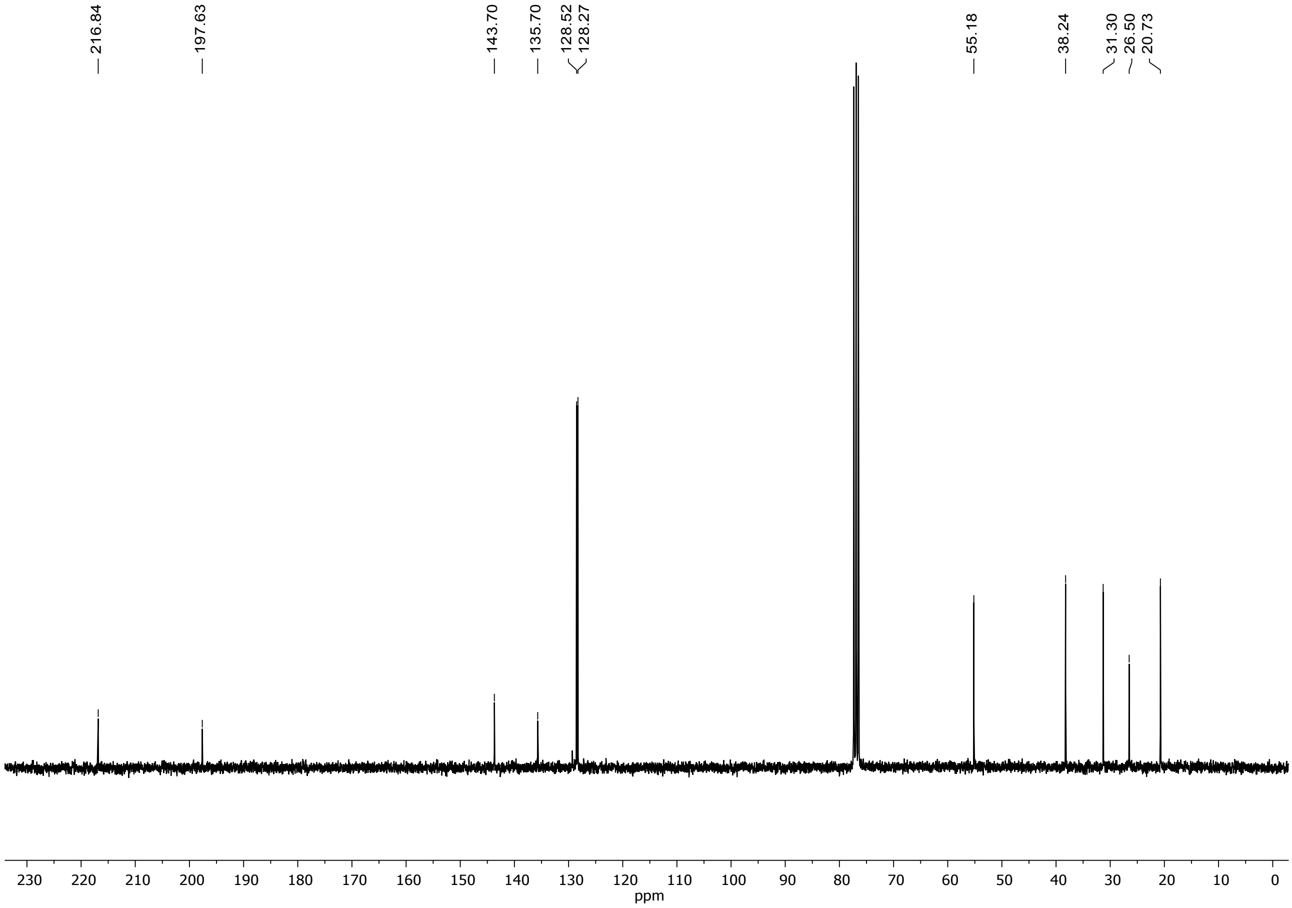

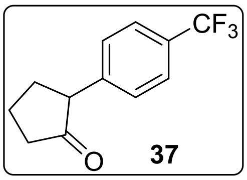

^{1}H NMR (300 MHz, $CDCl_3$)

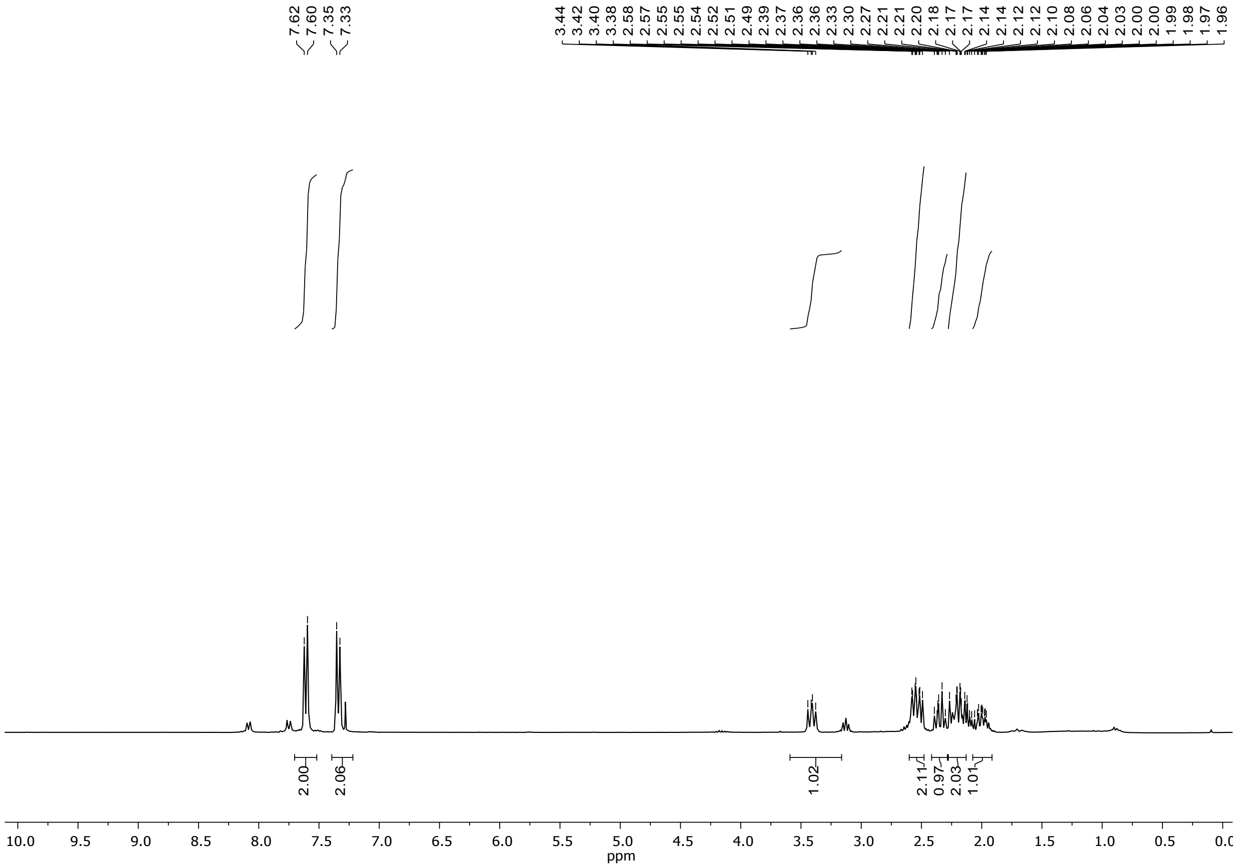

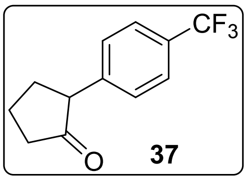

37

^{13}C NMR (300 MHz, $CDCl_3$)

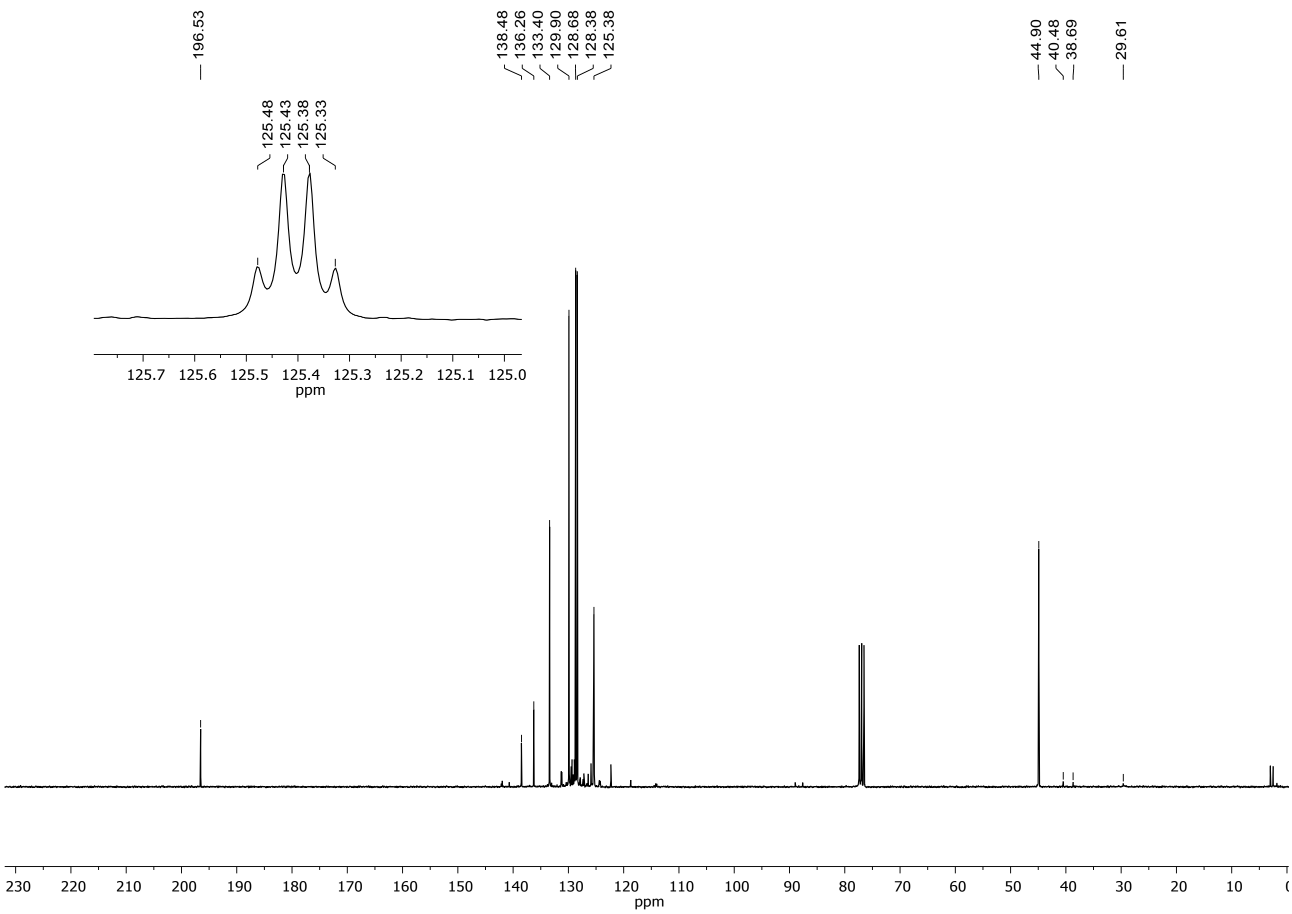

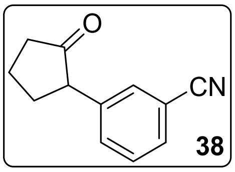

^{1}H NMR (300 MHz, $CDCl_3$)

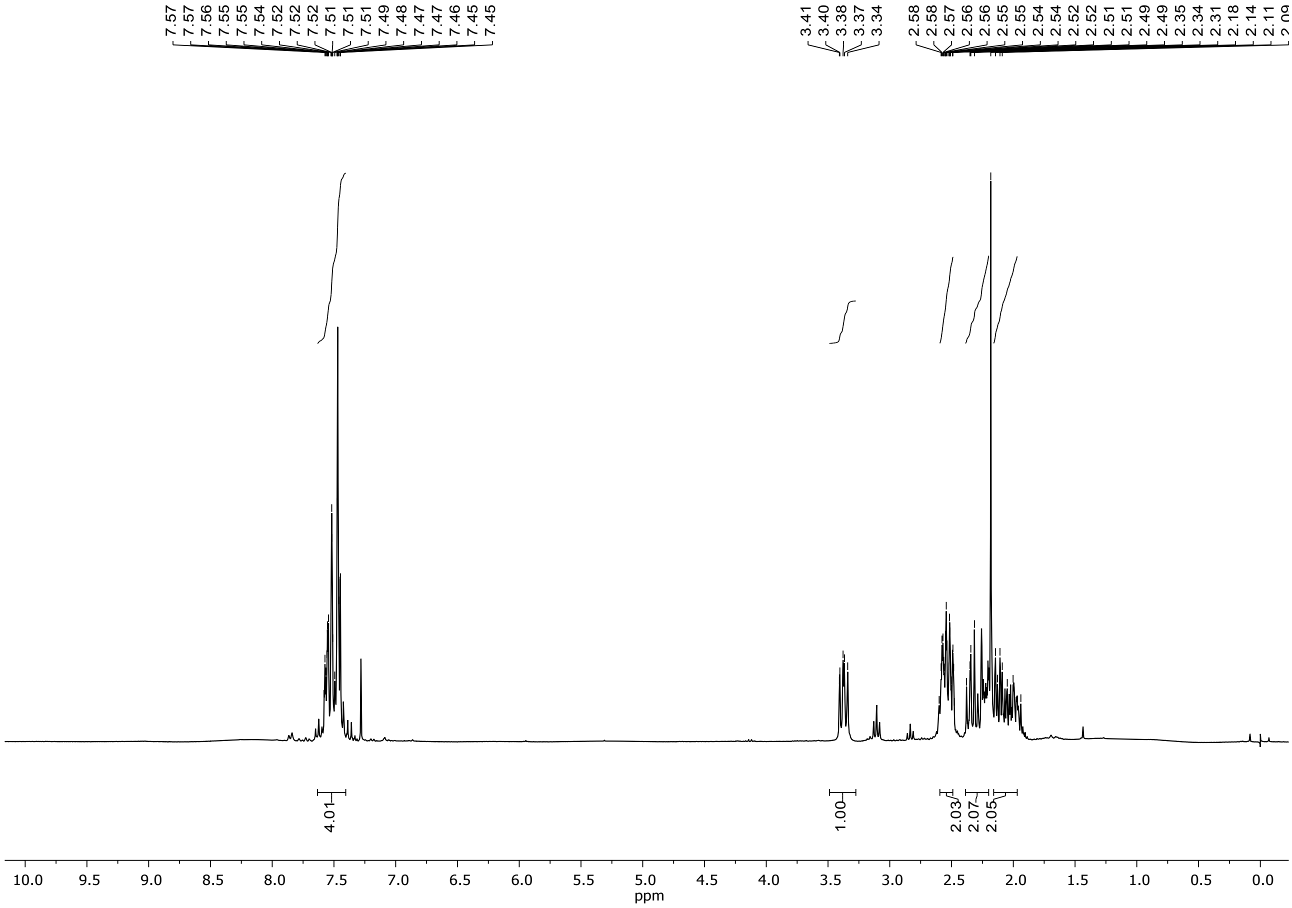

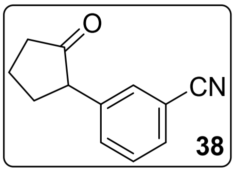

^{13}C NMR (75 MHz, $CDCl_3$)

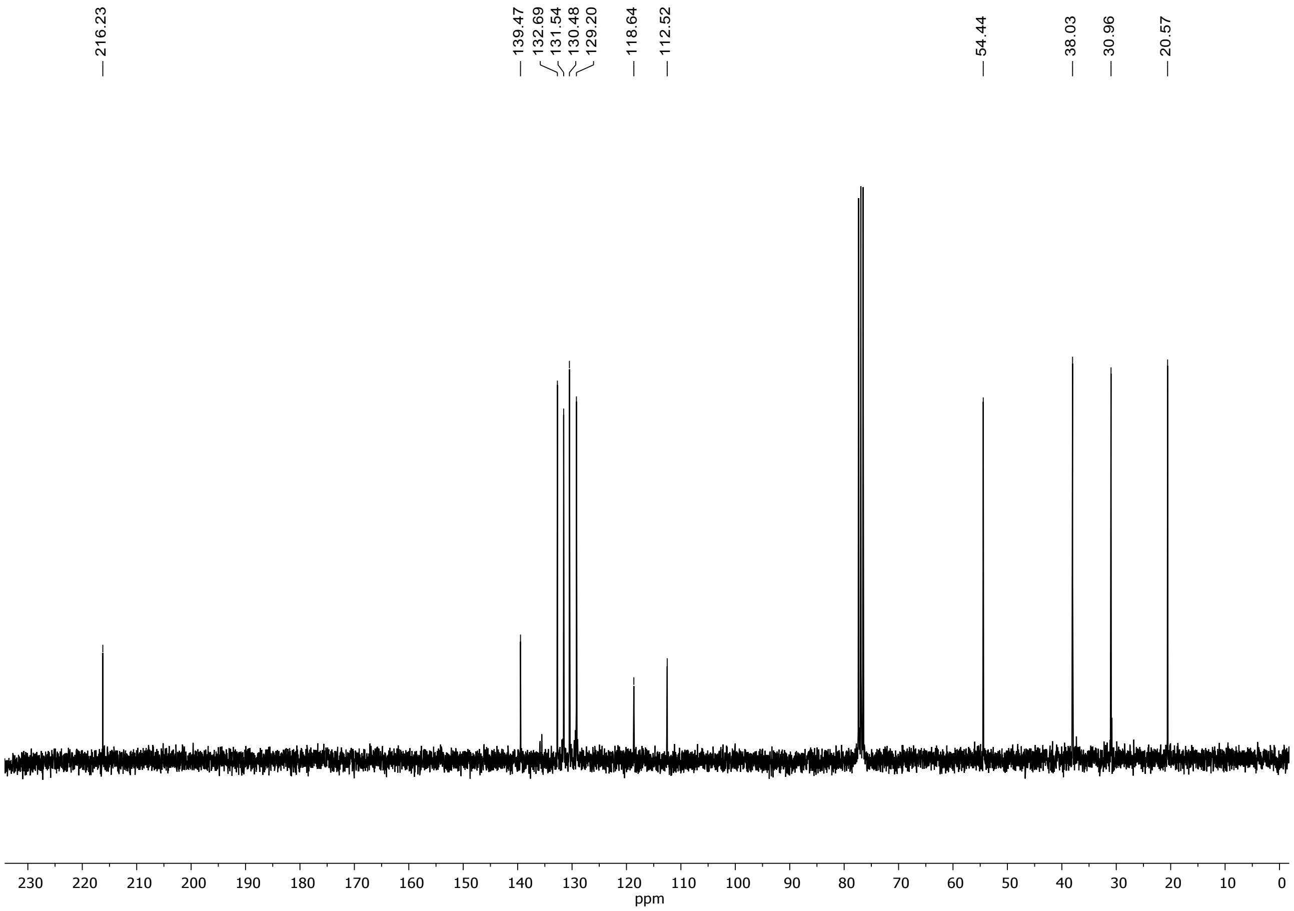

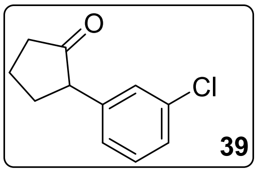

39

^{1}H NMR (300 MHz, $CDCl_3$)

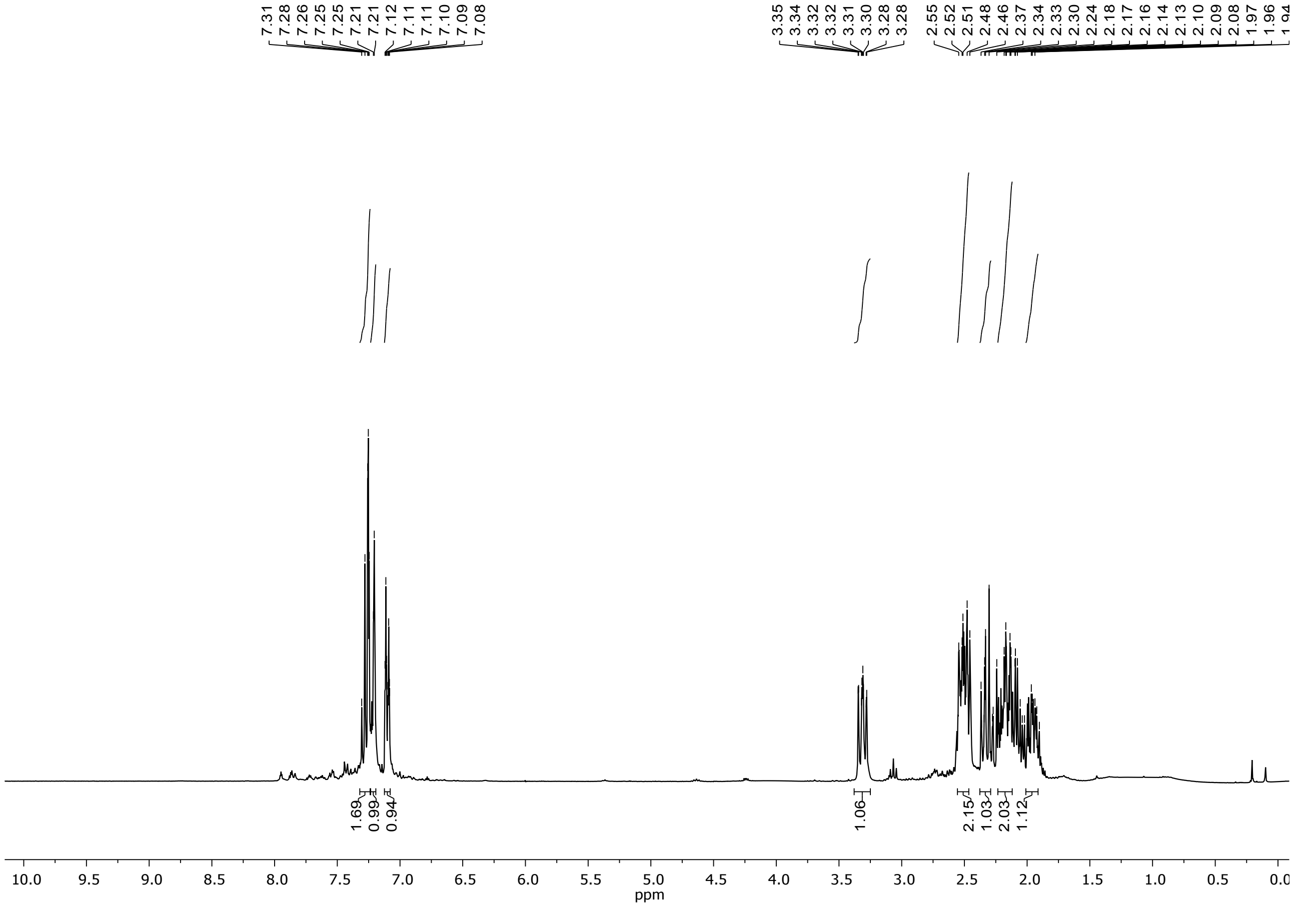

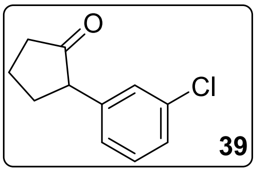

39 ^{13}C NMR (75 MHz, $CDCl_3$)

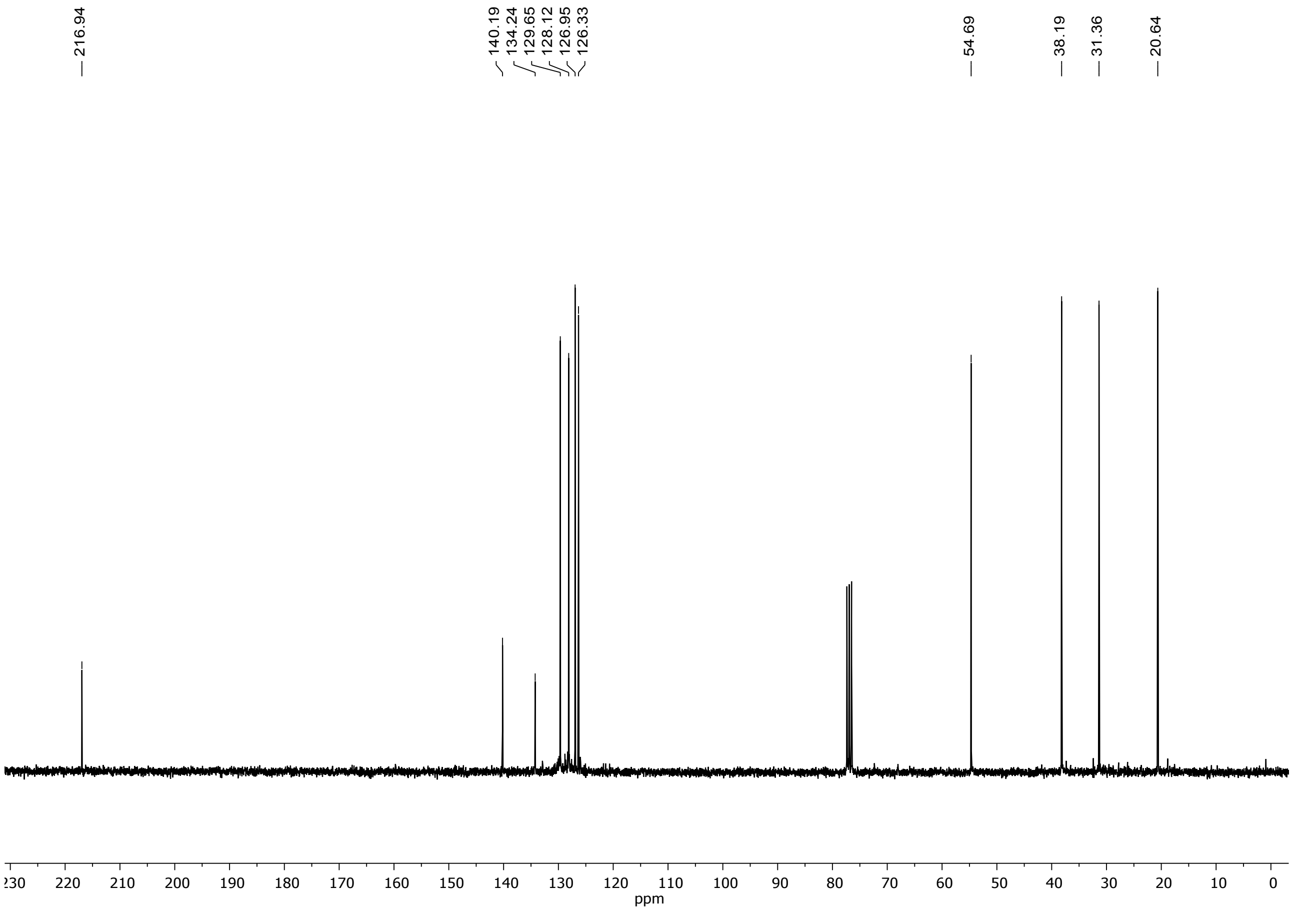

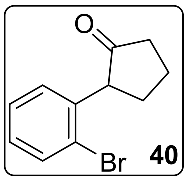

40

^1H NMR (300 MHz, $CDCl_3$)

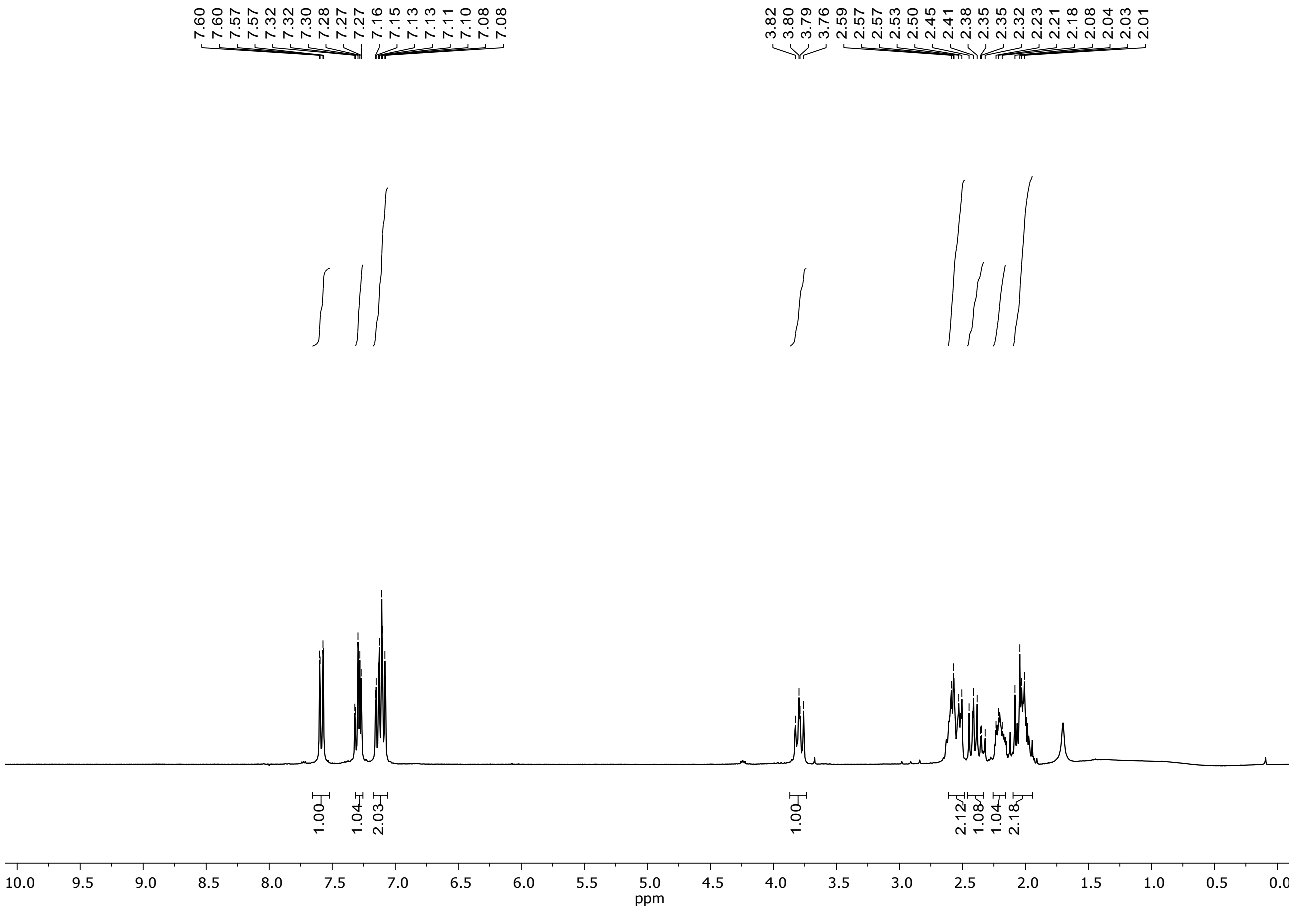

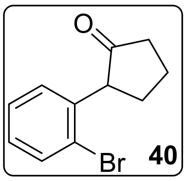

^{13}C NMR (75 MHz, $CDCl_3$)

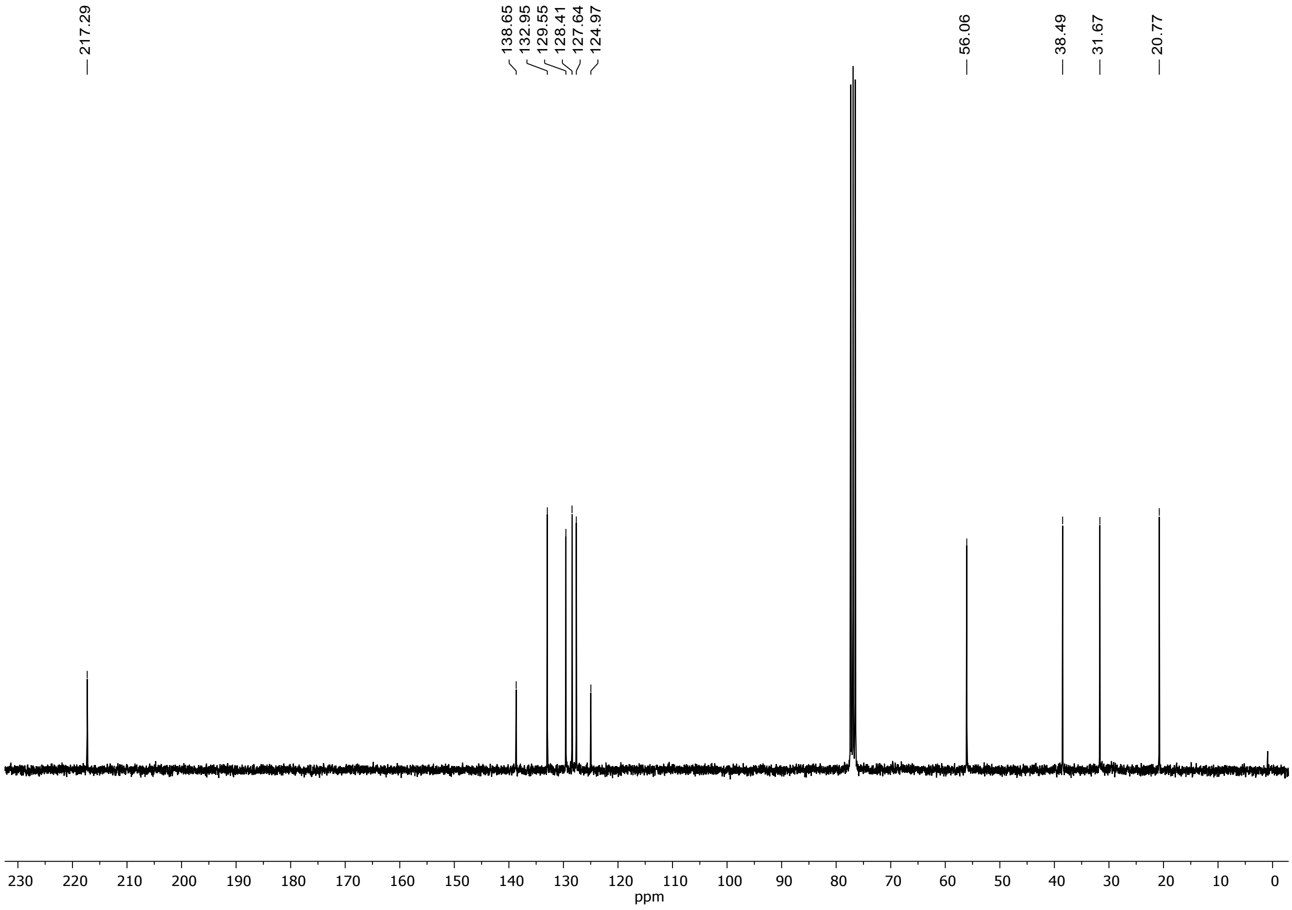

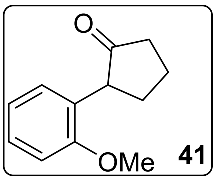

41

^{1}H NMR (300 MHz, $CDCl_3$)

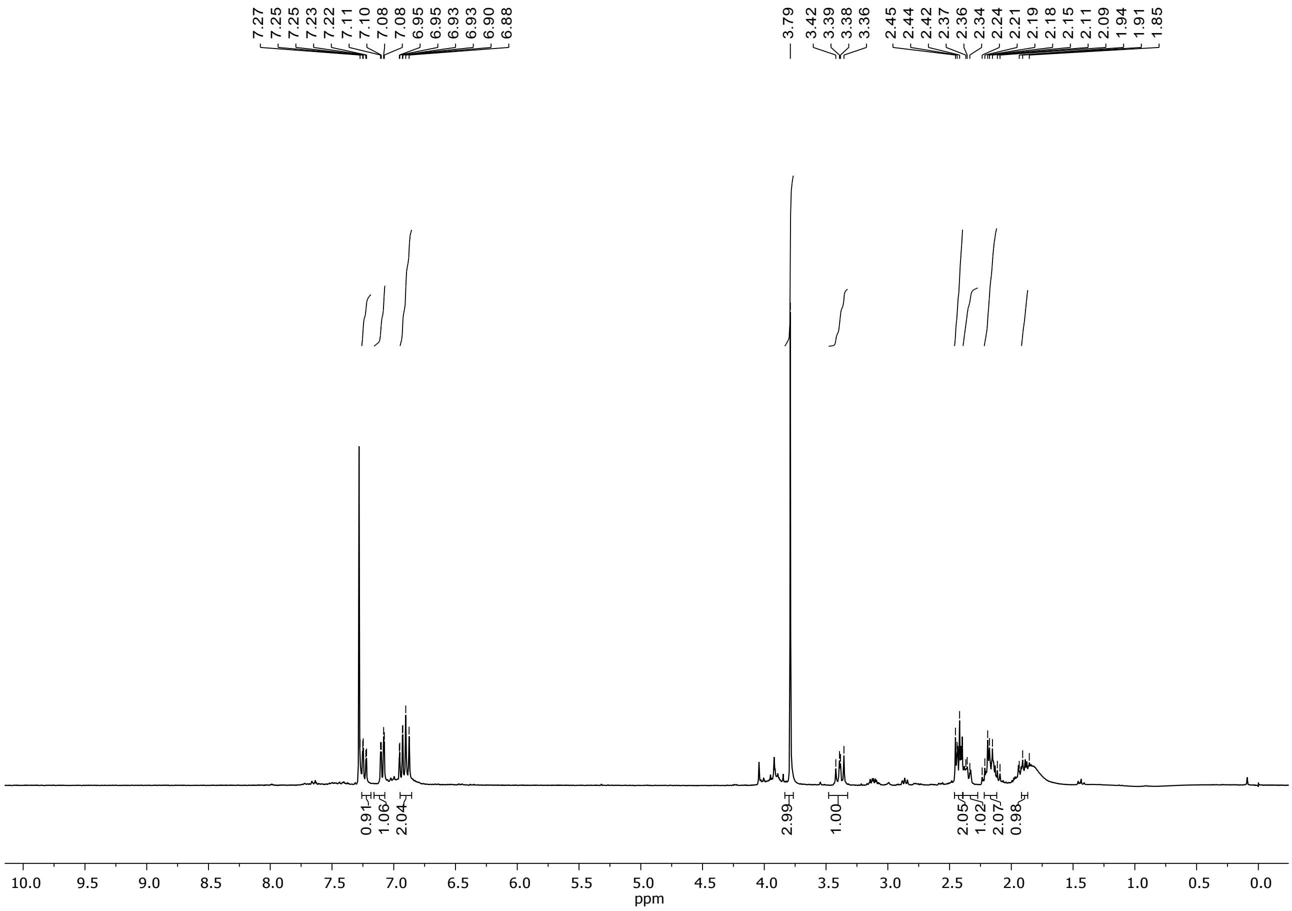

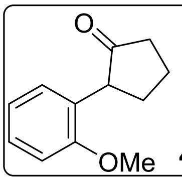

41

^{13}C NMR (75 MHz, $CDCl_3$)

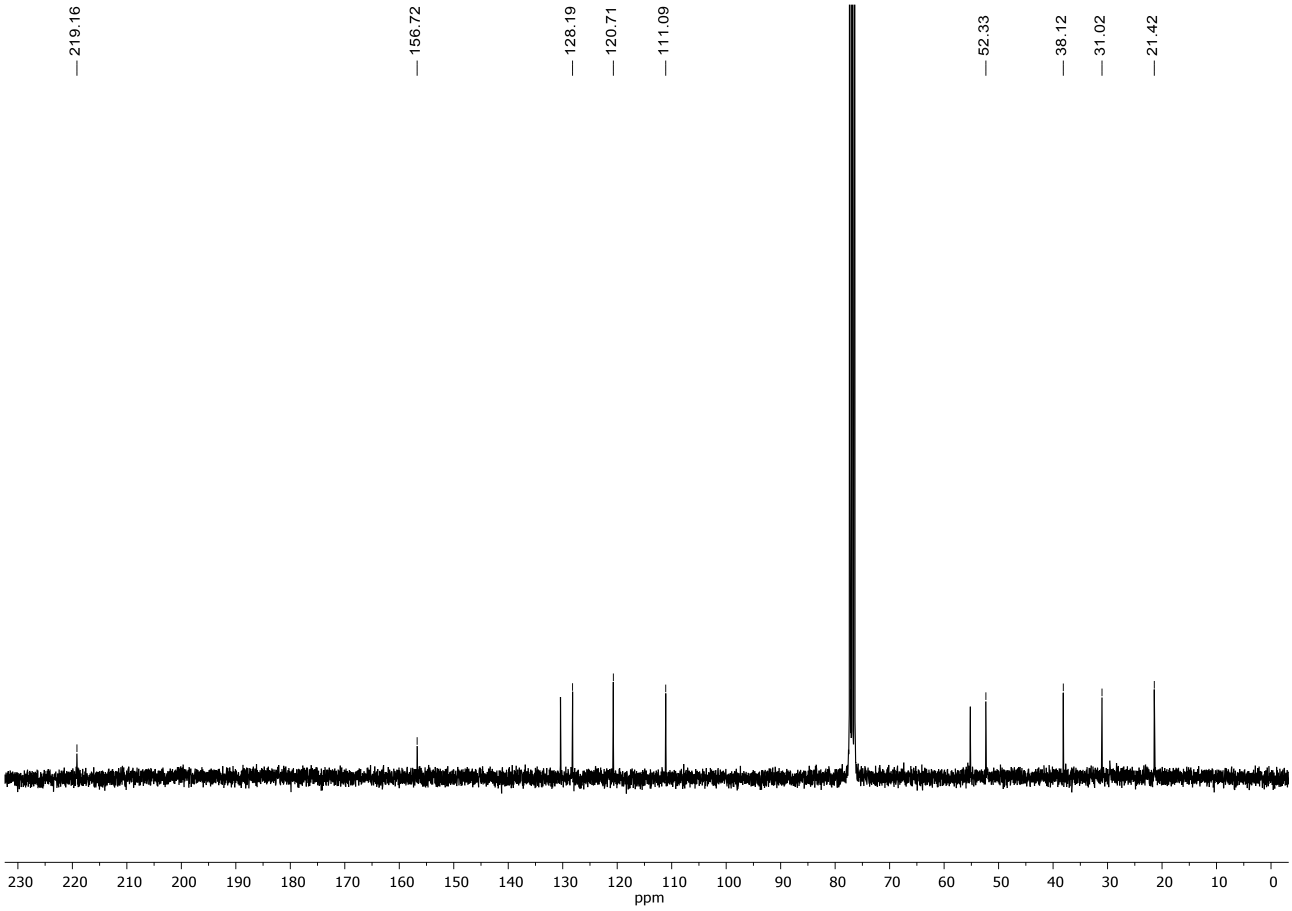

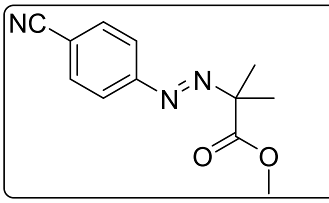

42

^{1}H NMR (300 MHz, $CDCl_3$)

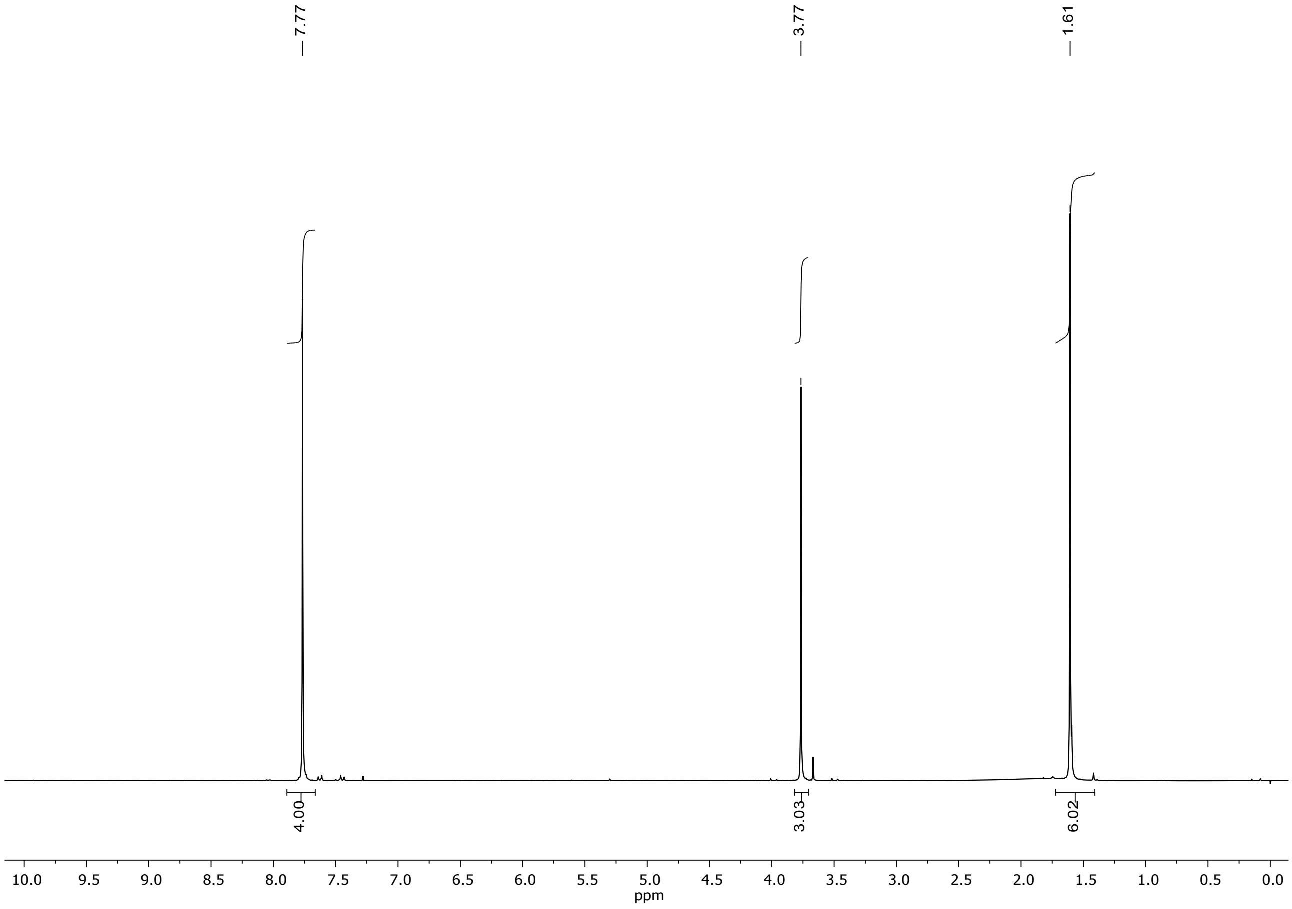

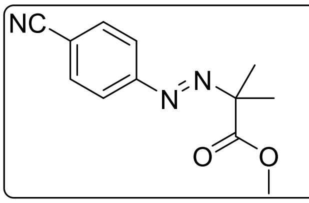

42

^{13}C NMR (75 MHz, $CDCl_3$)

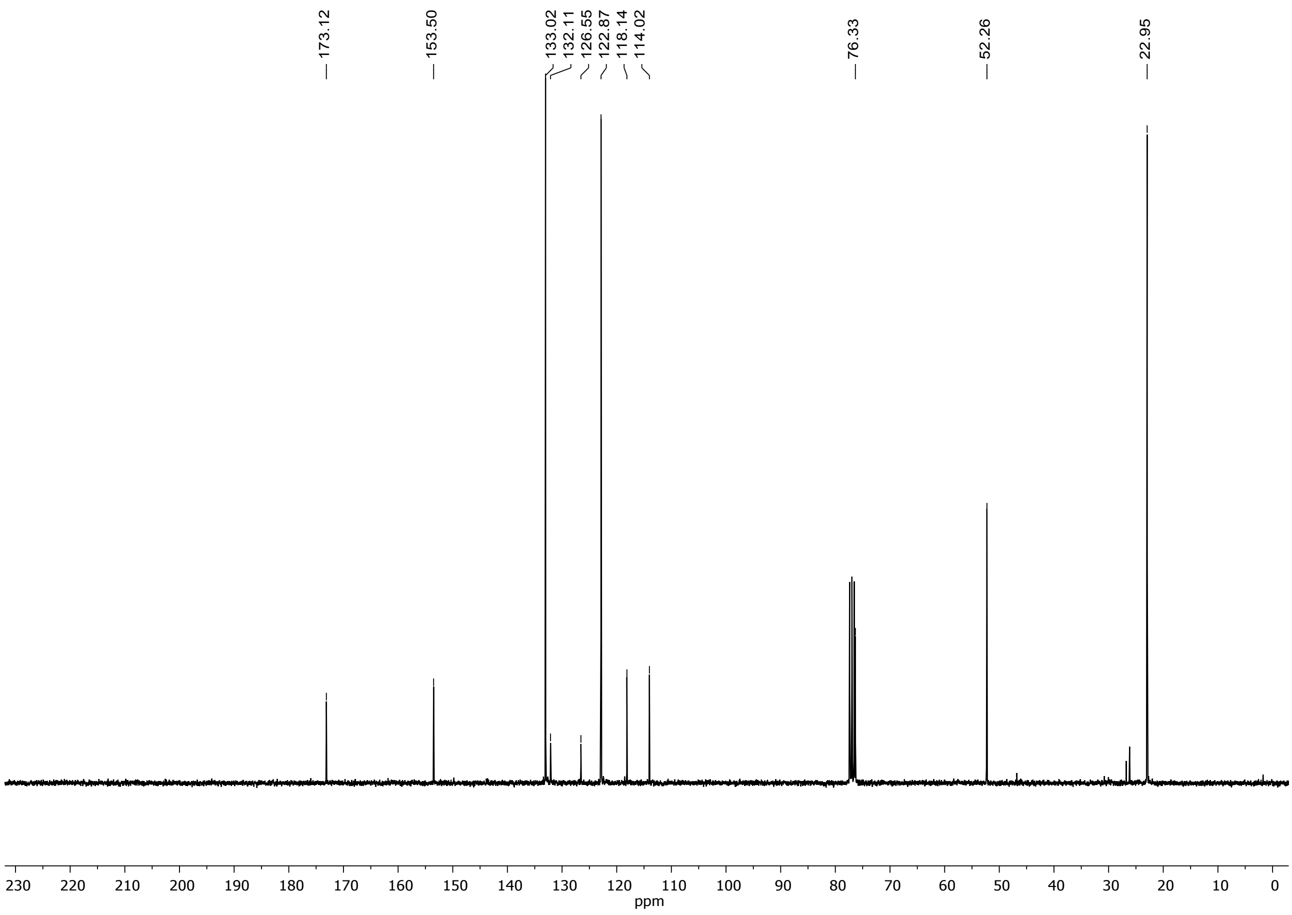

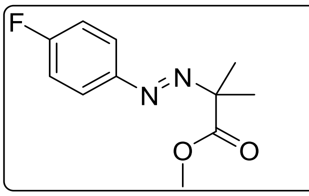

43

^{1}H NMR (300 MHz, $CDCl_3$)

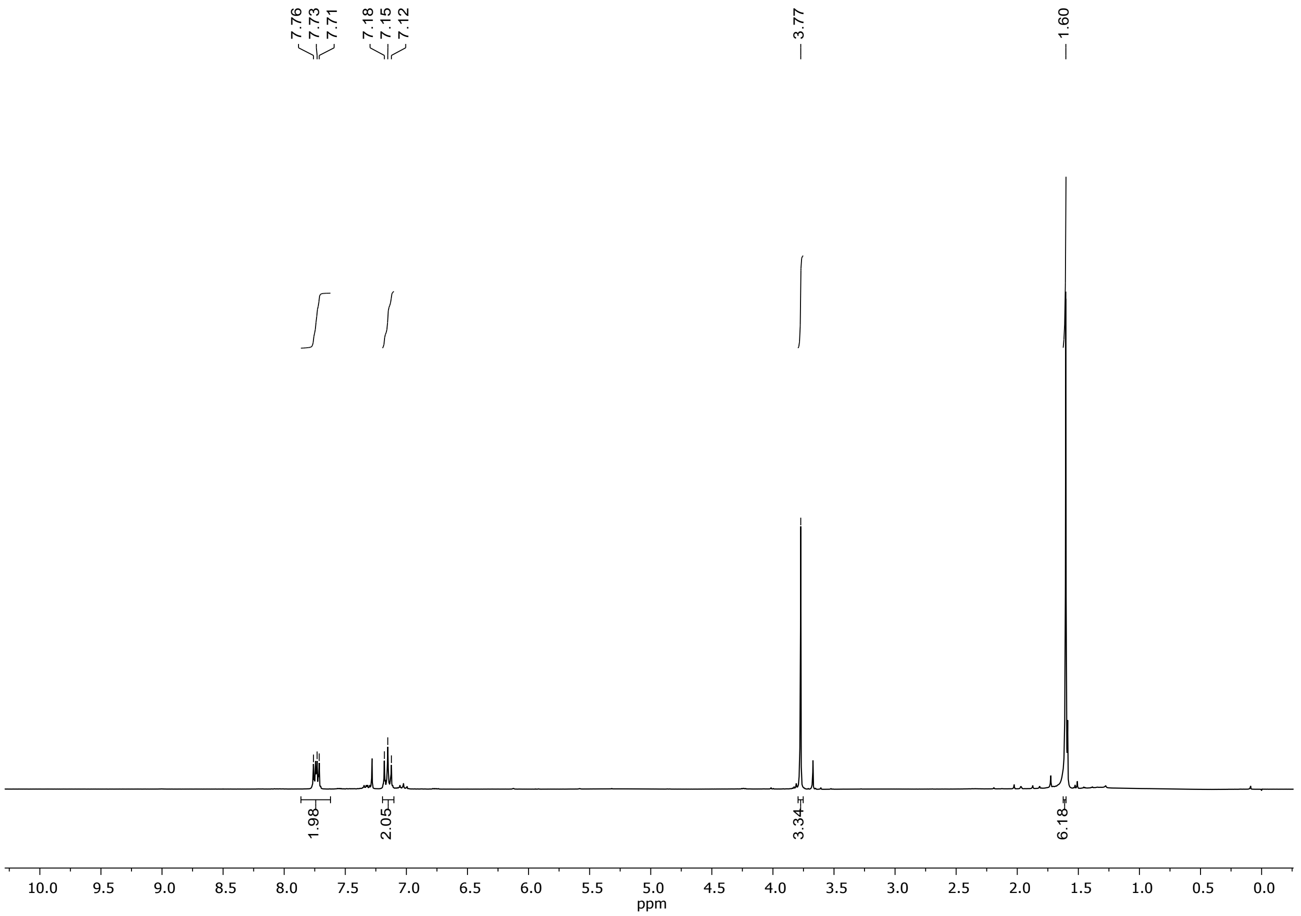

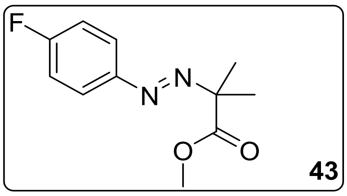

43

^{13}C NMR (75 MHz, $CDCl_3$)

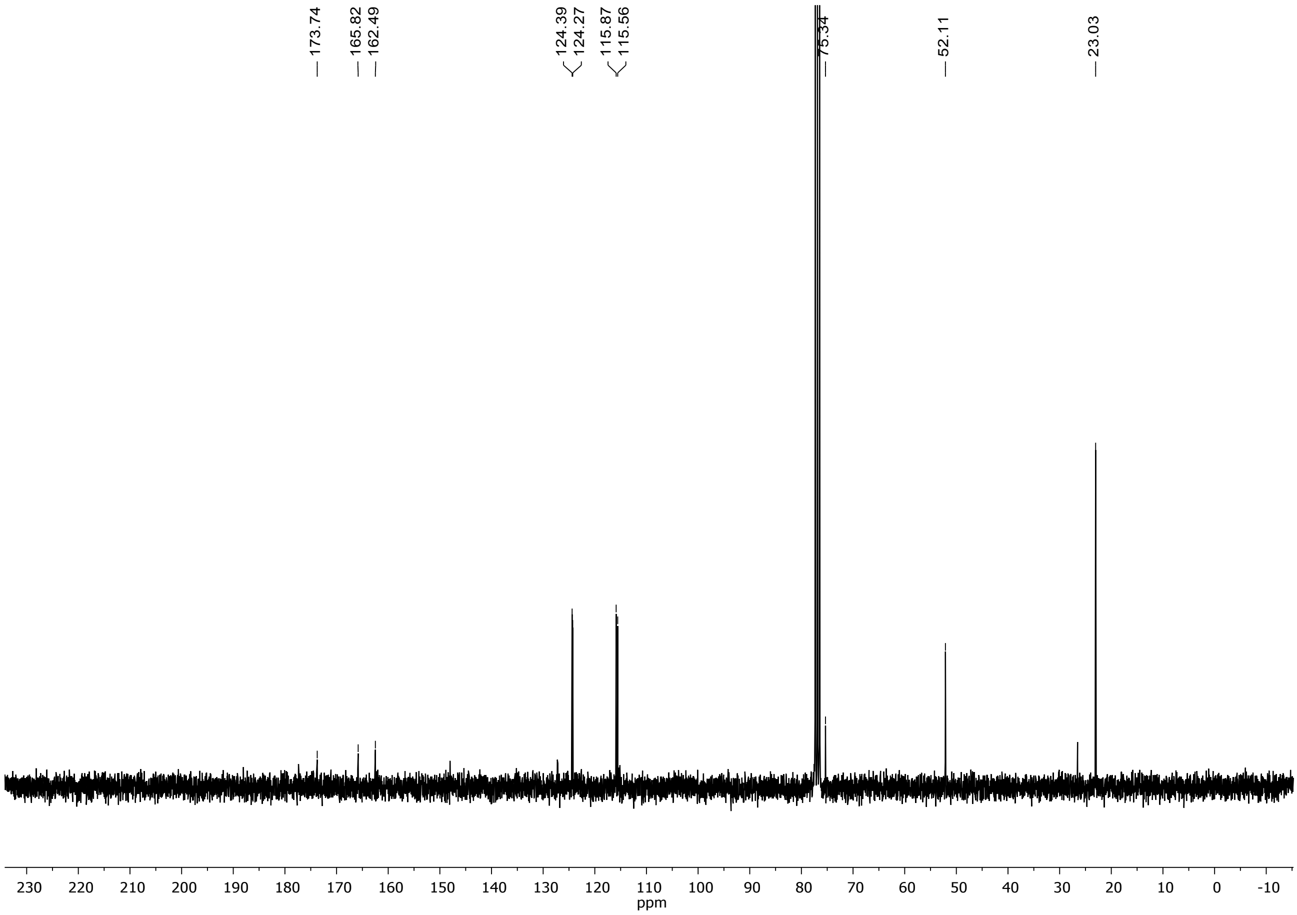

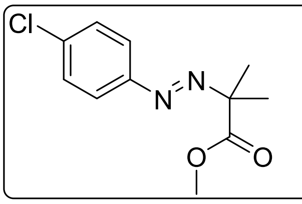

44

^1H NMR (300 MHz, $CDCl_3$)

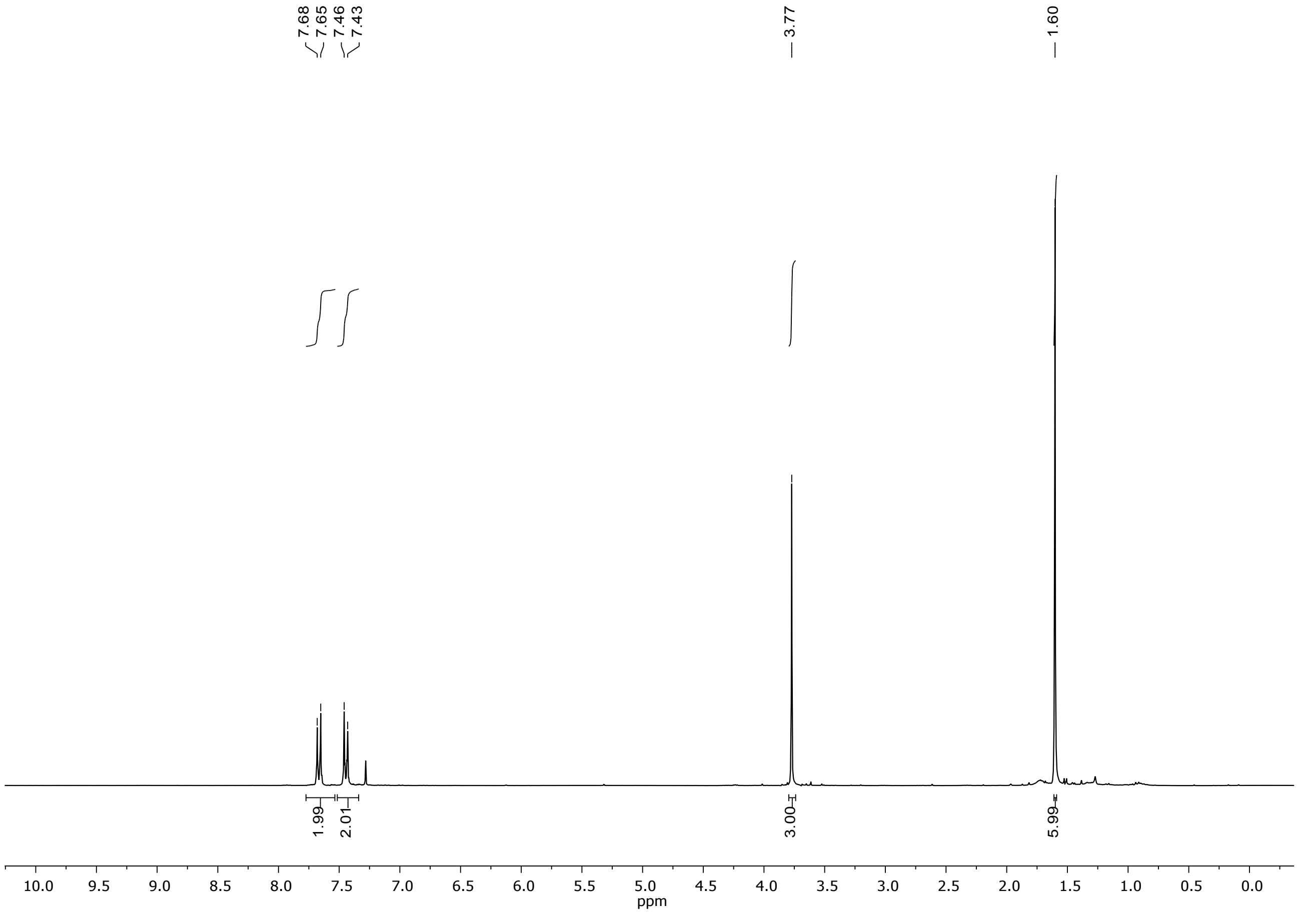

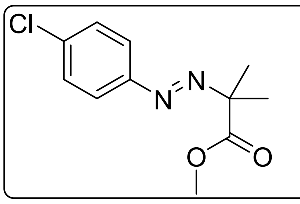

44

^{13}C NMR (75 MHz, $CDCl_3$)

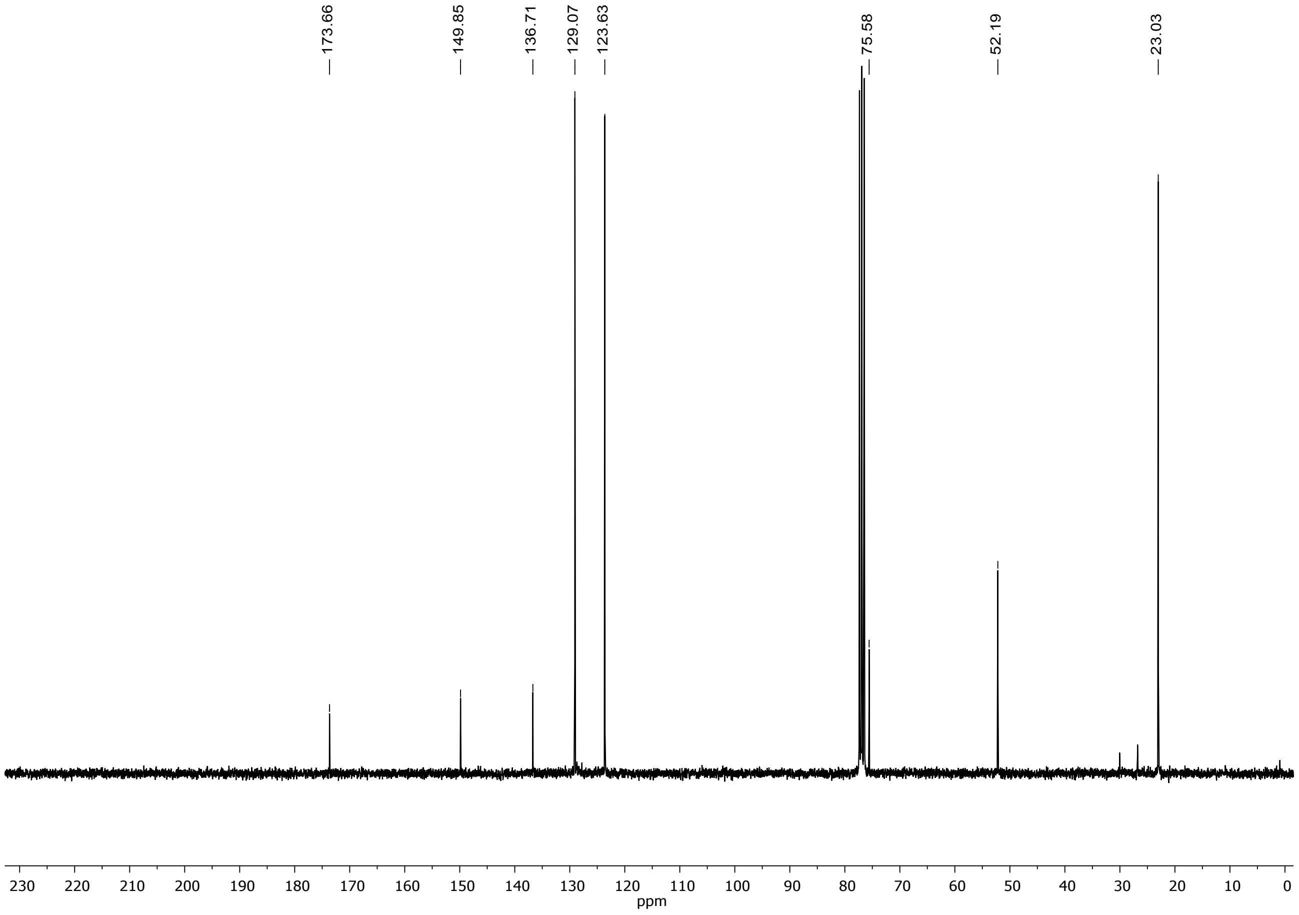

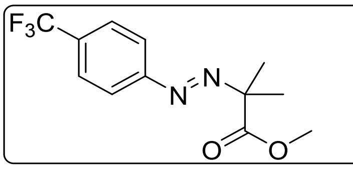

45

^{1}H NMR (300 MHz, $CDCl_3$)

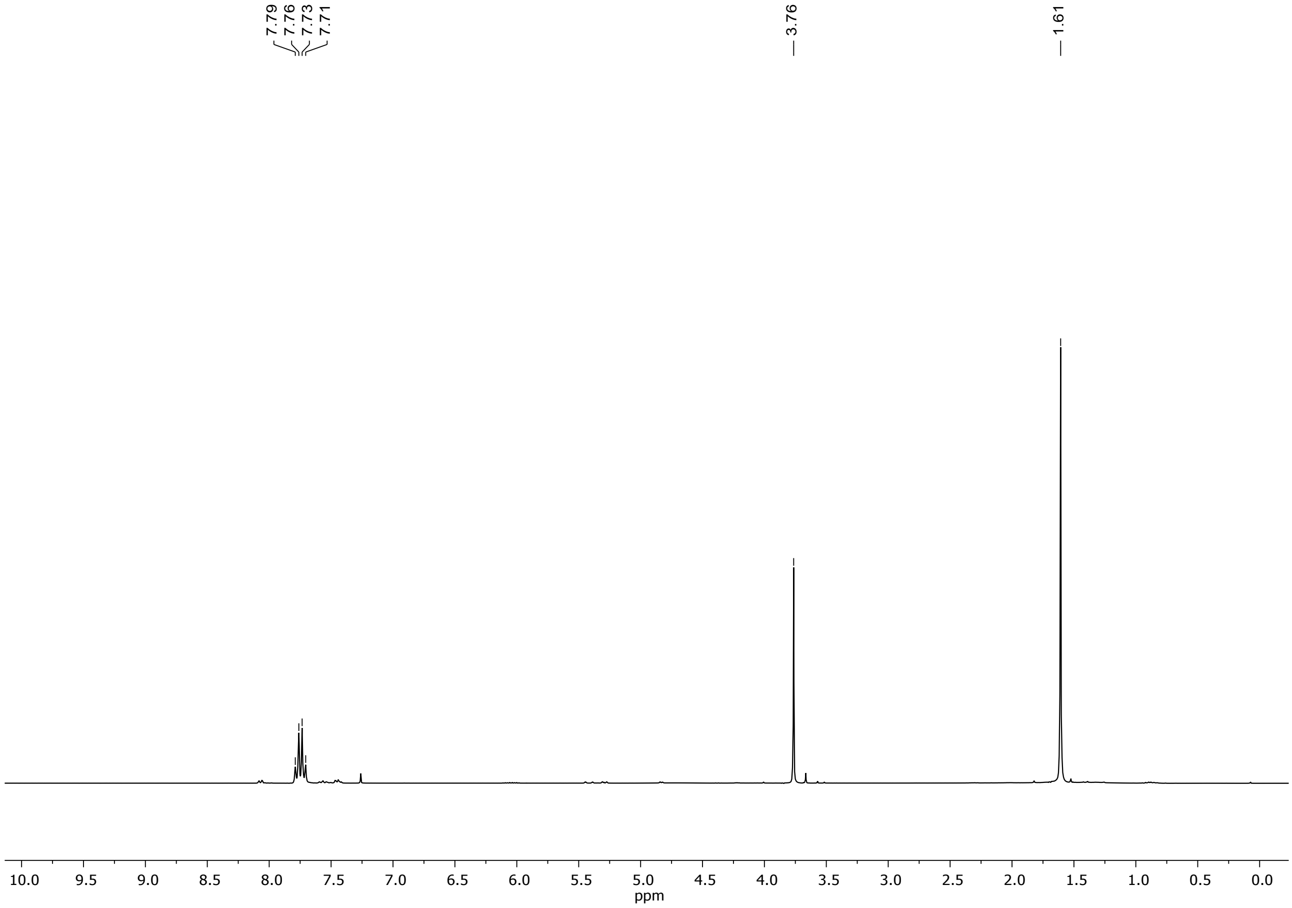

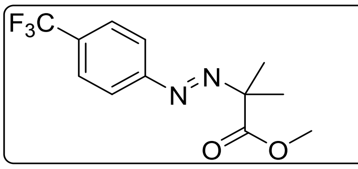

45

^{13}C NMR (75 MHz, $CDCl_3$)

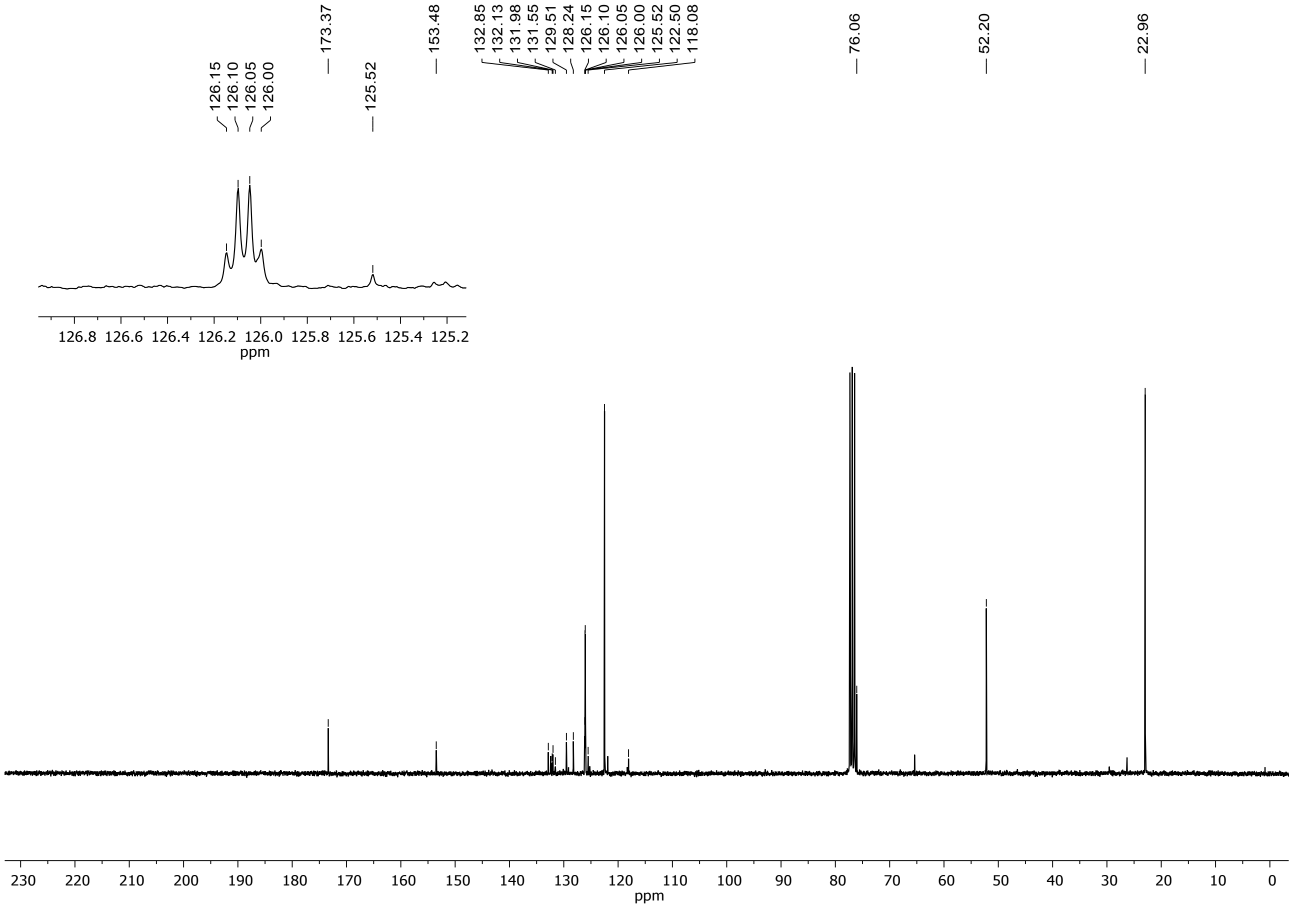

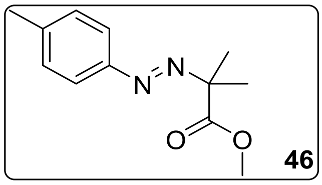

46

^{1}H NMR (300 MHz, $CDCl_3$)

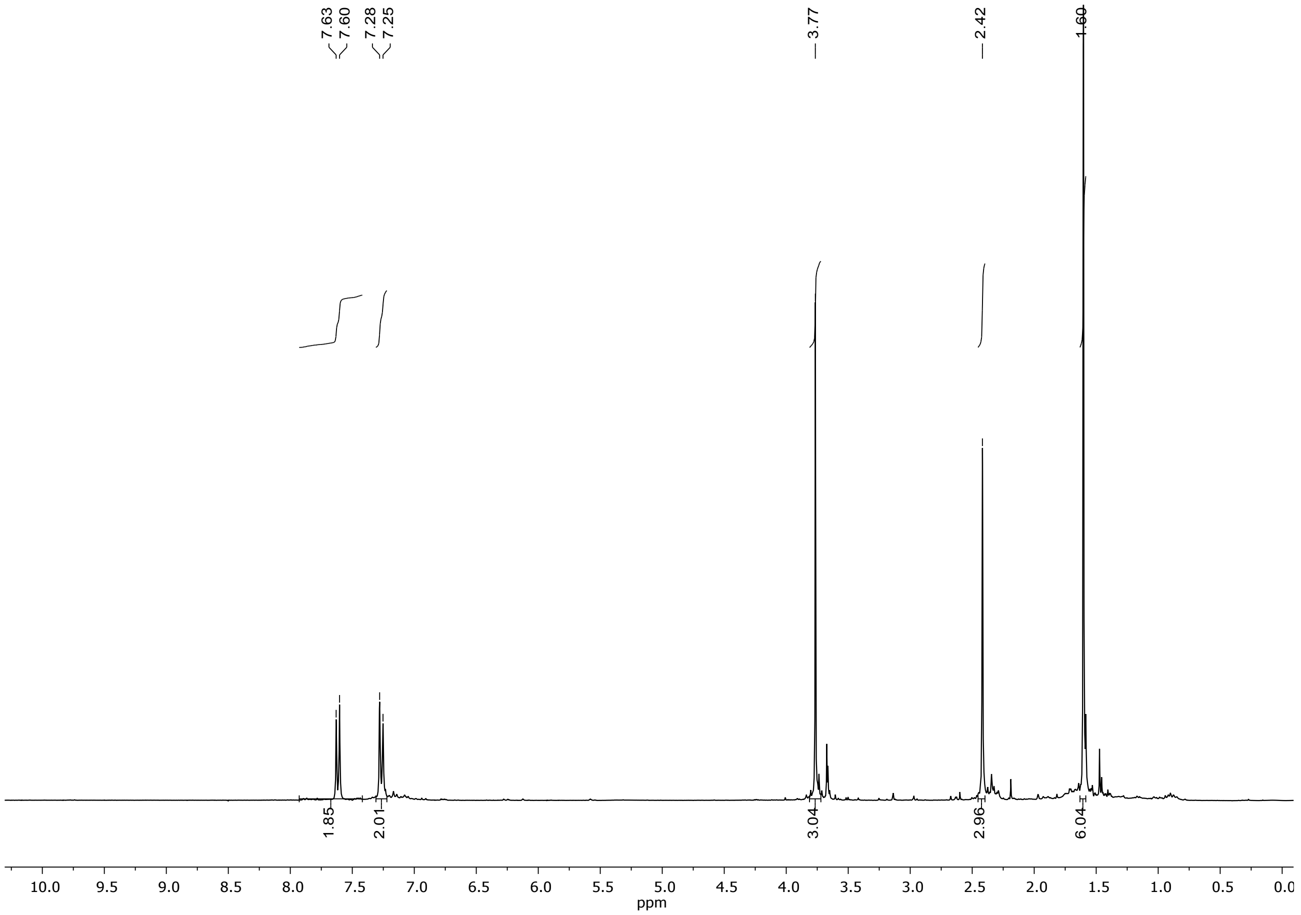

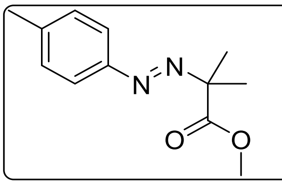

46

^{13}C NMR (75 MHz, $CDCl_3$)

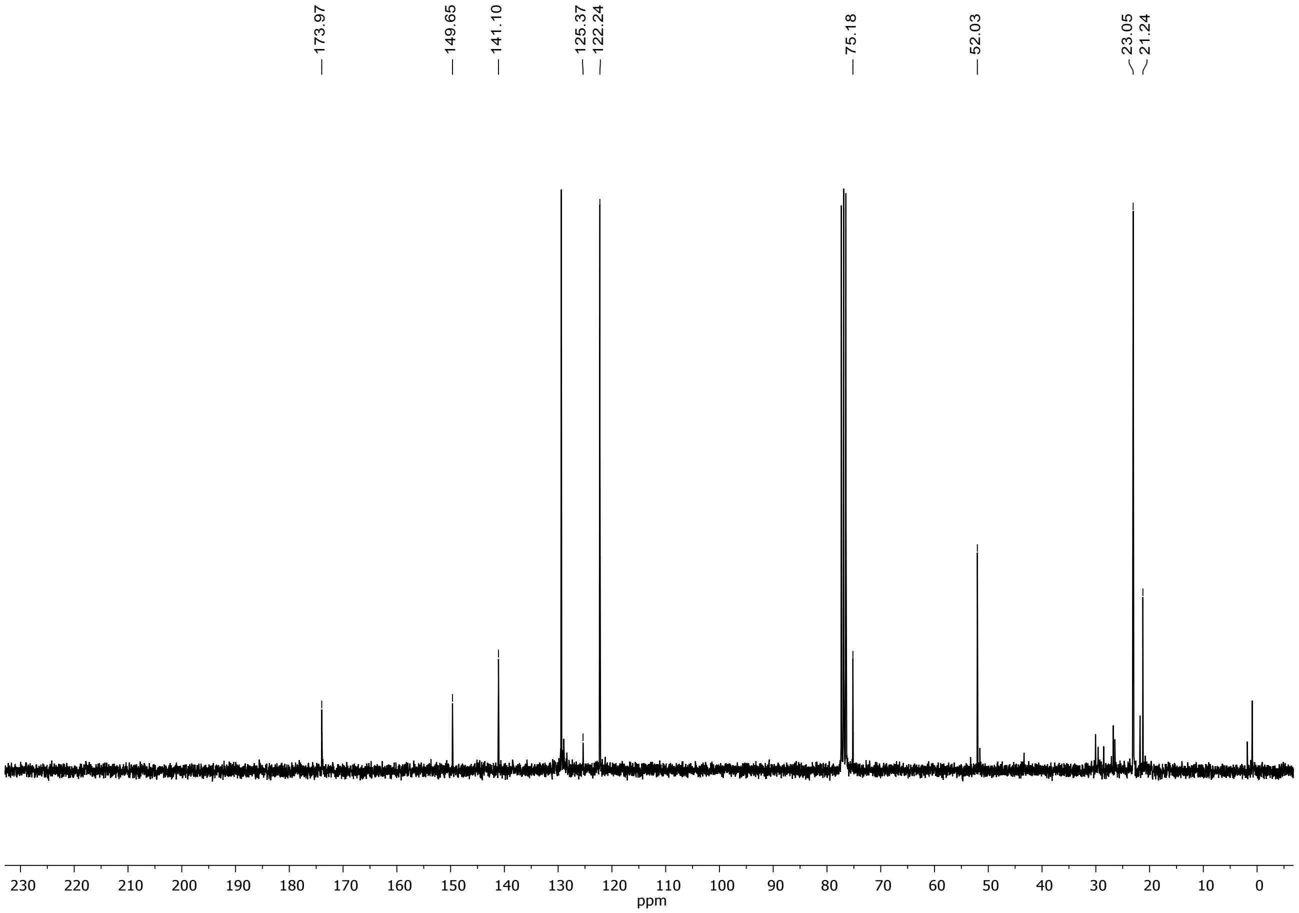

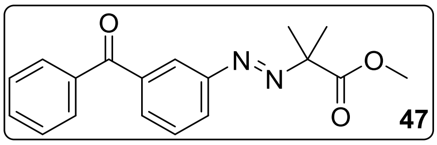

47

^{1}H NMR (300 MHz, $CDCl_3$)

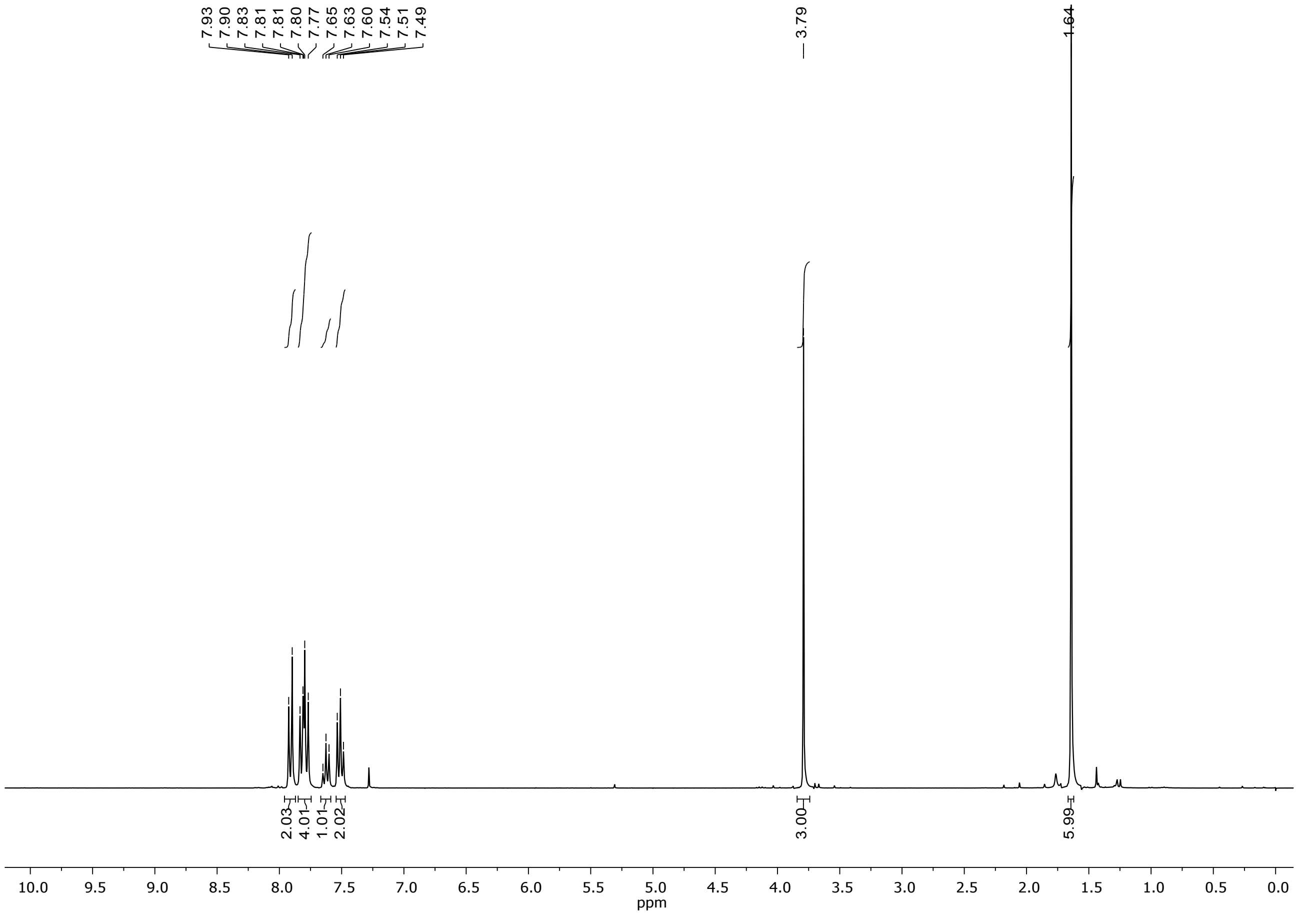

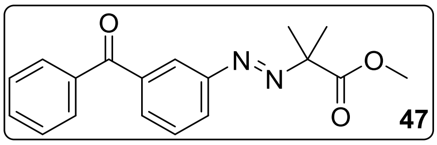

47

^{13}C NMR (75 MHz, $CDCl_3$)

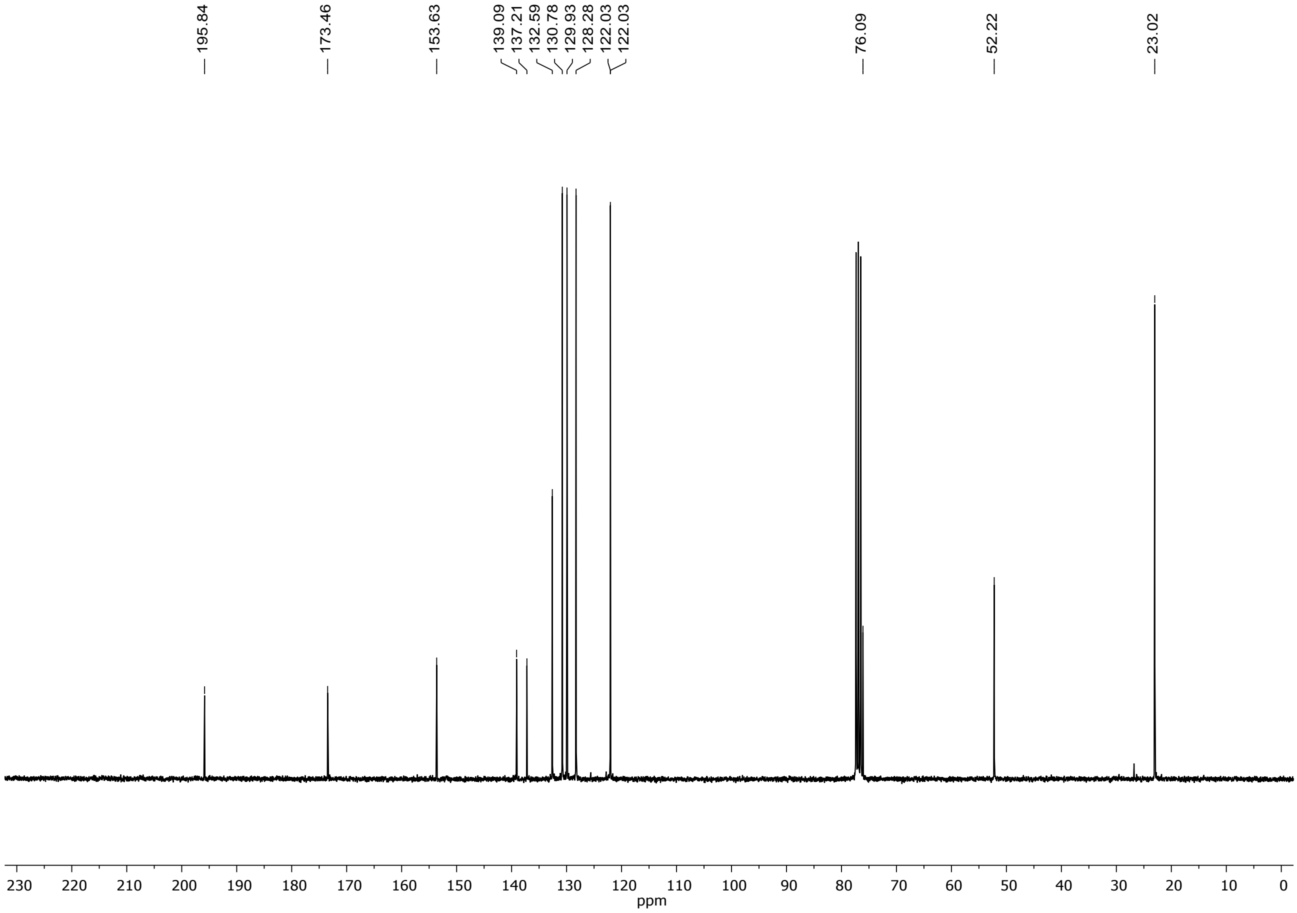

3. References:

S1 Malacarne, M.; Protti, S.; Fagnoni, M. *Adv. Synth. Catal.* **2017**, *359*, 3826-3830.

S2 (a) Crespi, S.; Protti, S.; Fagnoni, M. *J. Org. Chem.* **2016**, *81*, 9612–9619; (b) Lian, C.; Yue, G.; Mao, J.; Liu, D.; Ding, Y.; Liu, Z.; Qiu, D.; Zhao, X.; Lu, K.; Fagnoni, M.; Protti, S. *Org. Lett.* **2019**, *21*, 5187-5191; (c) Li, A.; Li, Y.; Liu, J.; Chen, J.; Lu, K.; Qiu, D.; Fagnoni, M.; Protti,S.; Zhao, X. *J. Org. Chem*. **2021**, *86*, 1292–1299.

S3 Scamehorn, R. G.; Bunnett, J. F. *J. Org. Chem.* **1979**, *44*, 2604–2608.

S4 Prashad, M.; Liu, Y.; Repic, O. *Adv. Synth. Catal*. **2003**, *345*, 533-536.

S5 Salamanca, V.; Albéniz, A. C. *Org. Chem. Front.*, **2021**, *8*, 1941-1951.

S6 Crawford, S. M.; Alsabeh, P. G.; Stradiotto, M. *Eur. J. Org. Chem.* **2012**, 6042–6050.

S7 Metzger, A.; Schade, M. A.; Knochel, P. *Org. Lett.* **2008**, *10*, 1107–1110.

S8 Moine, E.; Denevault-Sabourin, C.; Debierre-Grockiego, F.; Silpa, L.; Gorgette, O.; Barale, J.; Jacquiet, P.; Brossier, F.; Gueiffier, A.; Dimier-Poisson, I.; Enguehard-Gueiffier, C. *Eur. J. Med. Chem.* **2015**, *89*, 386–400.

S9 Nguyen, H. N.; Huang, X; Buchwald, S. L. *J. Am. Chem. Soc.* **2003**, *125*, 11818–11819.

S10 (*a*) Norris, R. K. *ARKIVOC*, **2003**, *10*, 139–155; (*b*) Takise, R.; Muto, K.; Yamaguchi, J.; Itami, K. *Angew. Chem. Int. Ed.* **2014**, 53, 6791–6794.

S11 (*a*) Khan, R. A. *J. Pharm. Pharmacol.* **1949**, *V1*, 230–234; (*b*) Lin, H.; Ibrahim, N.; Provot, O.; Alami, M.; Hamze, A. *RSC Adv*., **2018**, *8*, 11536–11542.

S12 Ando T.; Yuki, *G.; Kagaku Kyokaishi* **1959**, *V17*, 777–782.

S13 Xie, S.; He, Z.; Zhang, Z.; Huang, B.; Chen, X.; Zhan, Z.; Zhang, F. *Chem. Commun.* **2021**, *57*, 2069–2072.

S14 Ramajayam, R.; Giridhar, R.; Yadav, M. R. *Chem. Het. Compounds*, **2006**, 42, 901–906.

S15 Shen, Z.; Xu , X.; Ji, S. *J. Org. Chem.*, **2010**, *75*, 1162–1167.

S16 Nie, X.; Huang, Y.; Wang, P. *Org. Lett.* **2020**, *22*, 7716–7720.

S17 Ye, C.; Twamley, B.; Shreeve, J. M. *Org. Lett.*, **2005**, *7*, 3961–3964.

S18 Wang, H.; Mueller, D. S.; Sachwani, R. M.; Kapadia, R.; Londino, H. N.; Anderson, L. L. *J. Org. Chem*. **2011**, *76*, 3203–3221.

S19 Inaba, S.; *J. Org. Chem*. **1985**, *50*, 1373–1381.

S20 Yuan, L. Renko, D.; Khelifi, I.; Provot, O.; Brion, J.; Hamze, A.; Alami, M. *Org. Lett.* **2016**, *18*, 3238–3241.

S21 Jenkins, S. S. *J. Am. Chem. Soc.* **1933**, *55*, 2896–2899

S22 Bradsher, C. K. *J. Org. Chem*. **1978**, *43*, 3817–3820.

S23 Yang, H.; Wan, D. *Org. Lett.* **2021**, *23*, 1049–1053.

S24 Mahecha-Mahecha, C.; Lecornué, F.; Akinari, S.; Charote, T.; Gamba-Sánchez, D.; Ohwada, T.; Thibaudeau, S. *Org. Lett.* **2020**, *22*, 6267–6271.

S25 Farago, J.; Kotschy, A. *Synthesis* **2009**, 85–90

S26 Hu, R.; Tao, R.; Zhang, X.; Su, W. *Angew. Chem. Int. Ed.* **2021**, *60*, 8425–8430.

S27 Hori, M.; Kataoka, T.; Shimizu, H.; Imai, E.; Iwamura, T.; Maeda, K. *Chem Pharm. Bull.* **1986**, *34*, 3599–3605.

S28 Huang, W.; Huang, G.; Zhu, W.; Weng, J.; Lu, G. *Org. Chem. Front.*, **2020**, 7, 2480–2485.

S29 Huitric, A. C.; Kumler, W. D. *J. Am. Chem. Soc.* **1956**, *78*, 614–622.

S30 Wang, T.; Wang, Y.; Wang, R.; Zhang, B.; Yang, C.; Li, Y.; Wang, X. *Nat Comm.* **2019**, *10*, 5373.

S31 Liu, Z.; Babu, K. R.; Wang, F.; Yang, Y.; Bi, X. *Org. Chem. Front.*, **2019**, *6*, 121–124.

S32 Xu, Y.; Su, T.; Huang, Z.; Dong, G. *Angew. Chem. Int. Ed.* **2016**, *55*, 559–563.

S33 Dabrowski, J. A.; Moebius, D. C.; Wommack, A. J.; Kornahrens, A. F.; Kingsbury, J. S. *Org. Lett.* **2010**, *12*, 3598–3601.

S34 Fusco, R. *Farmaco* **1965**, *20*, 393–407.

S35 Tomoyuki, S.; Eun-Cheol, S.; Kohei, T. *Bull. Chem. Soc. Jpn.* **2005**, *78*, 1654–1658.
